# Supplementary figures and images for: A mouse model of human mitofusin-2-related lipodystrophy exhibits adipose-specific mitochondrial stress and reduced leptin secretion (part 1 of 2)
Source: eLife. 2023 Feb 1;12:e82283. doi: 10.7554/eLife.82283 (PMC9937658; doi:10.7554/eLife.82283)

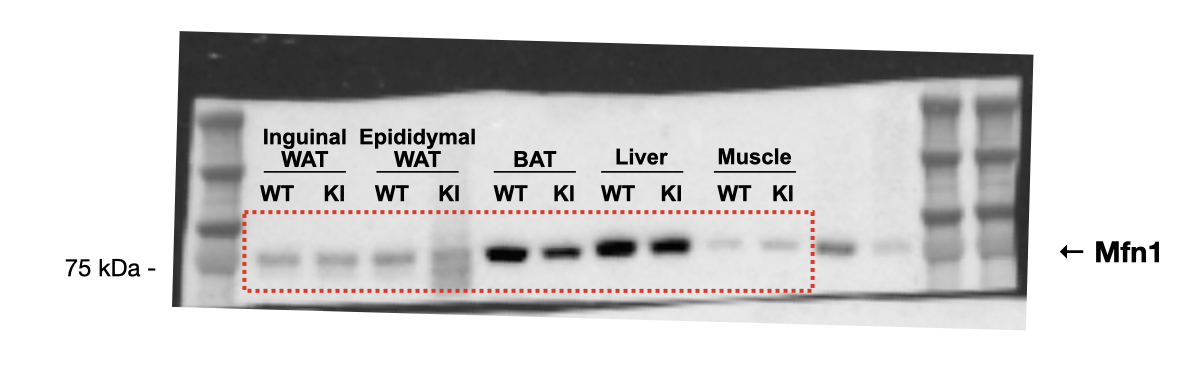

Supplement: Figure 1—source data 1. [file elife-82283-fig1-data1.zip › Fig1-Source data/Mfn1_annotated.jpeg]

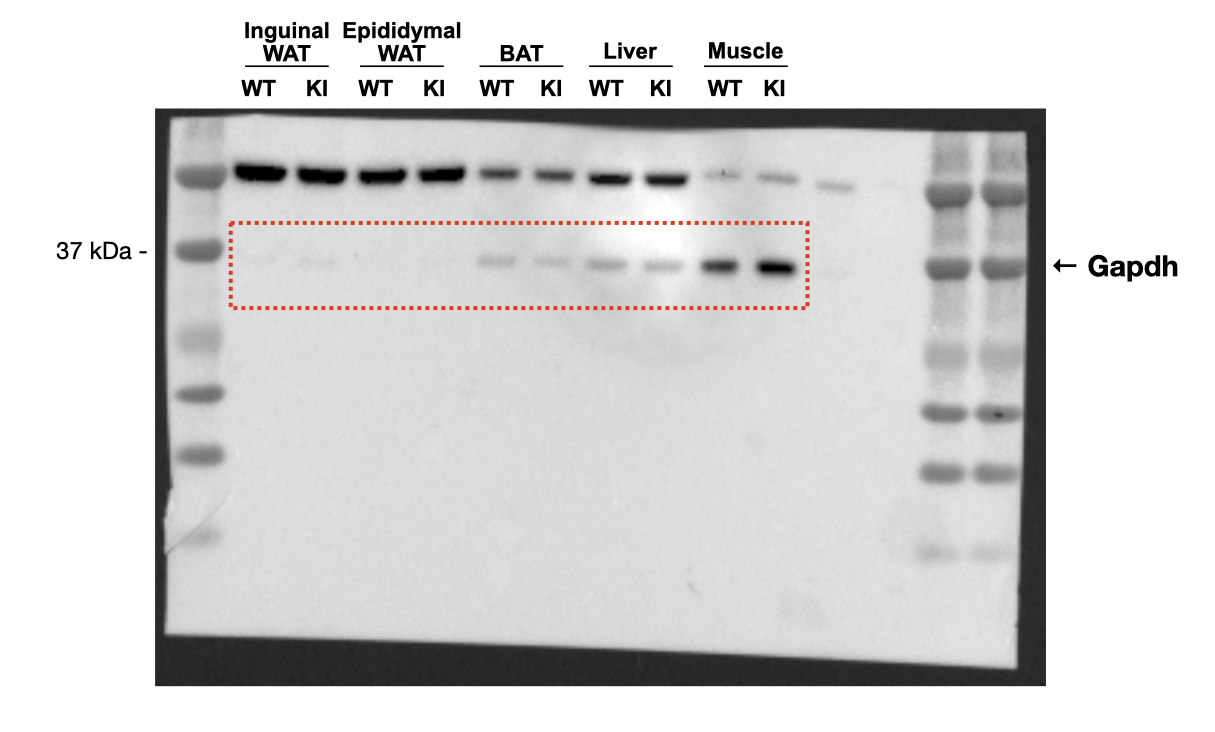

Supplement: Figure 1—source data 1. [file elife-82283-fig1-data1.zip › Fig1-Source data/Gapdh_annotated.jpeg]

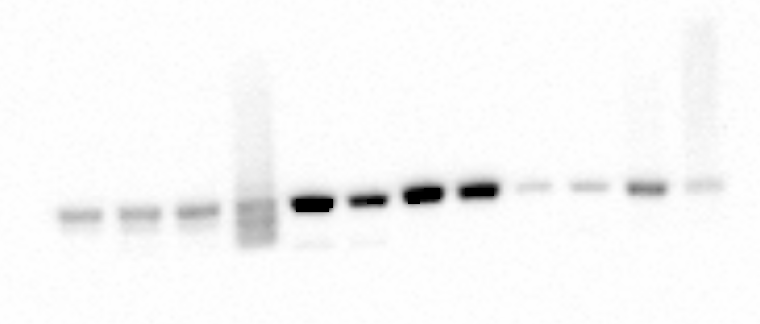

Supplement: Figure 1—source data 1. [file elife-82283-fig1-data1.zip › Fig1-Source data/Mfn1_raw.tif]

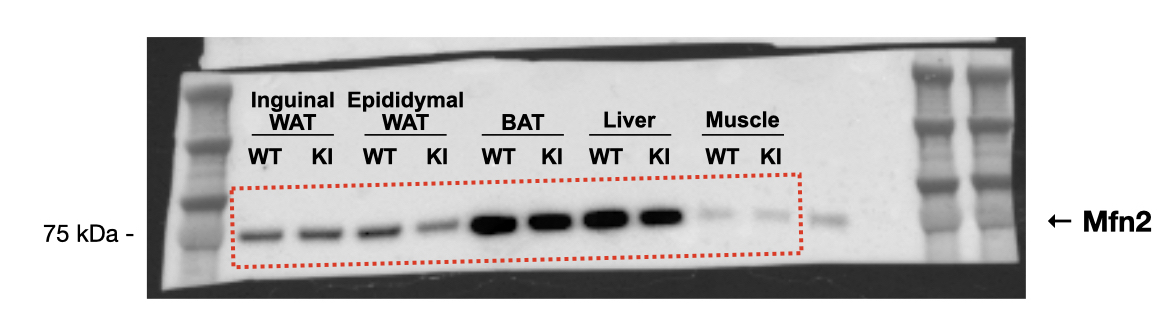

Supplement: Figure 1—source data 1. [file elife-82283-fig1-data1.zip › Fig1-Source data/Mfn2_annotated.jpeg]

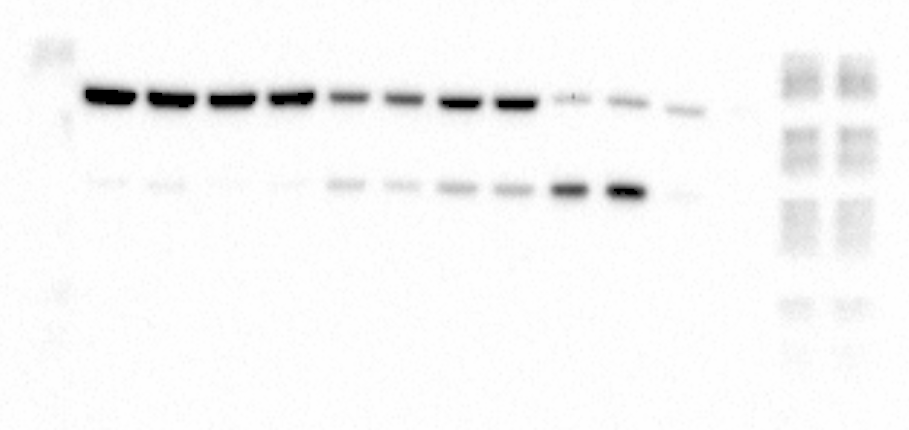

Supplement: Figure 1—source data 1. [file elife-82283-fig1-data1.zip › Fig1-Source data/Gapdh_raw.tif]

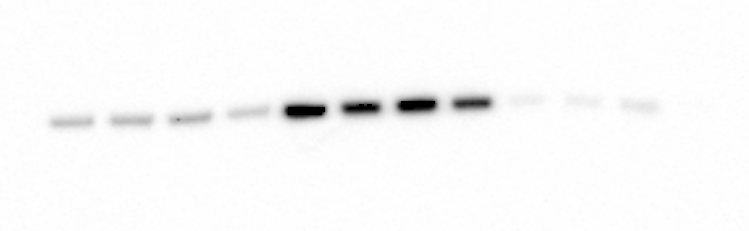

Supplement: Figure 1—source data 1. [file elife-82283-fig1-data1.zip › Fig1-Source data/Mfn2_raw.tif]

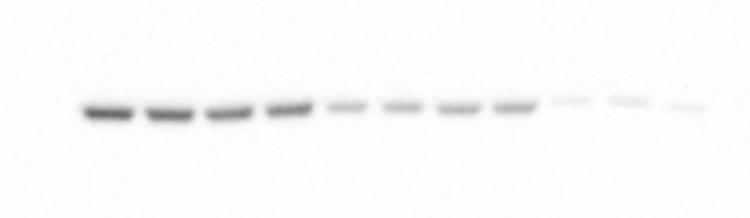

Supplement: Figure 1—source data 1. [file elife-82283-fig1-data1.zip › Fig1-Source data/Tubulin_raw.tif]

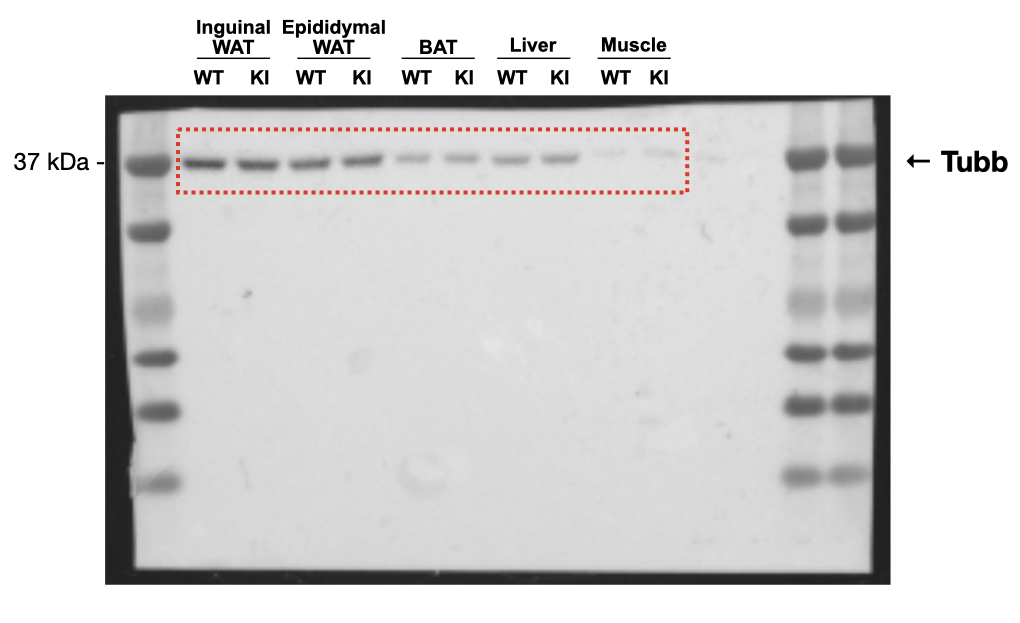

Supplement: Figure 1—source data 1. [file elife-82283-fig1-data1.zip › Fig1-Source data/Tubb_annotated.jpeg]

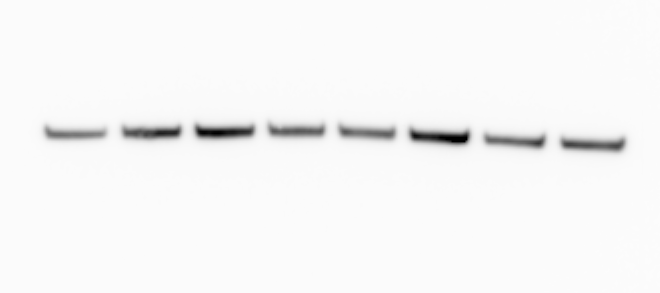

Supplement: Figure 1—figure supplement 1—source data 1. [file elife-82283-fig1-figsupp1-data1.zip › Fig1-SupFig1-Source data/EpiWAT_Mfn2_raw.tif]

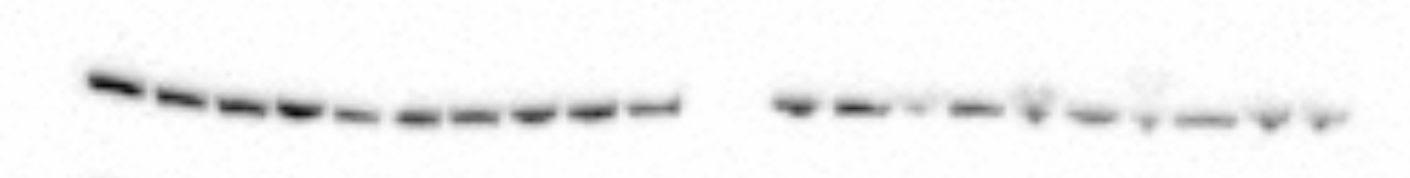

Supplement: Figure 1—figure supplement 1—source data 1. [file elife-82283-fig1-figsupp1-data1.zip › Fig1-SupFig1-Source data/Liver_Mfn2_raw.tif]

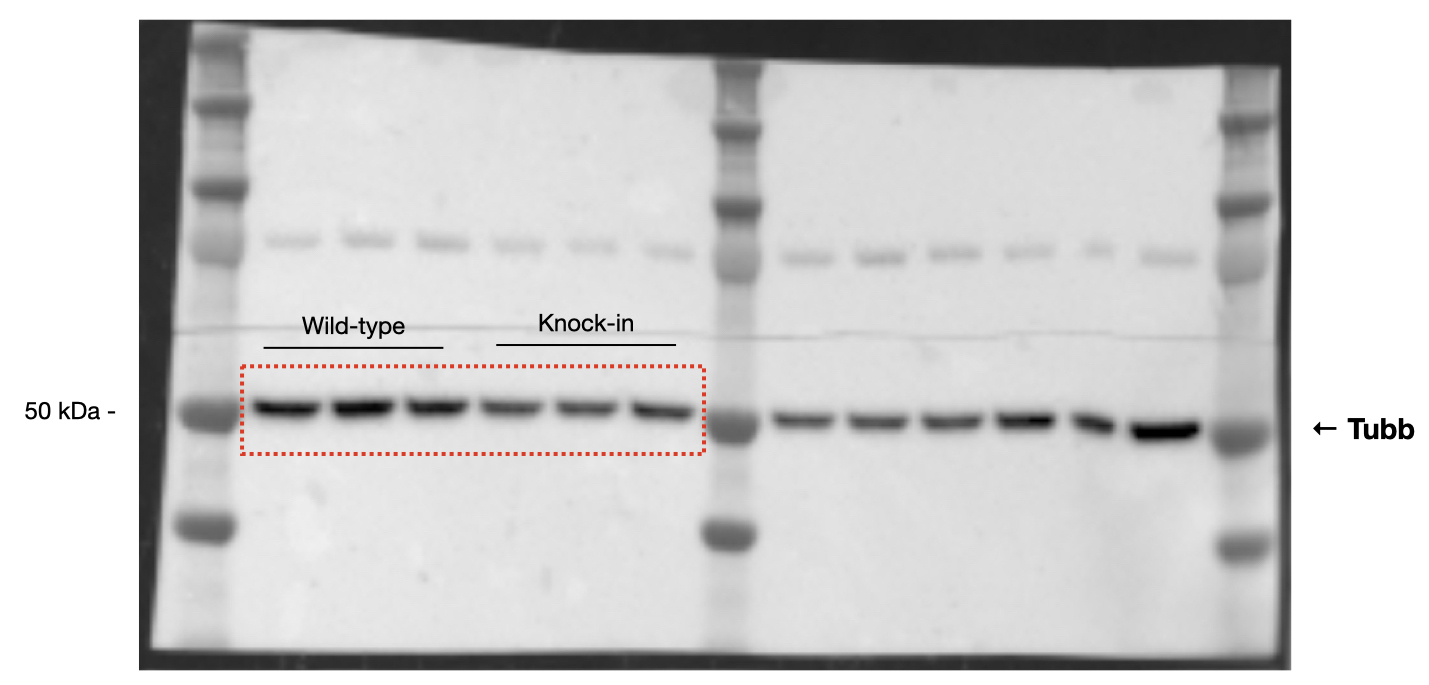

Supplement: Figure 1—figure supplement 1—source data 1. [file elife-82283-fig1-figsupp1-data1.zip › Fig1-SupFig1-Source data/SkelMusc_Tubb_annotated.jpeg]

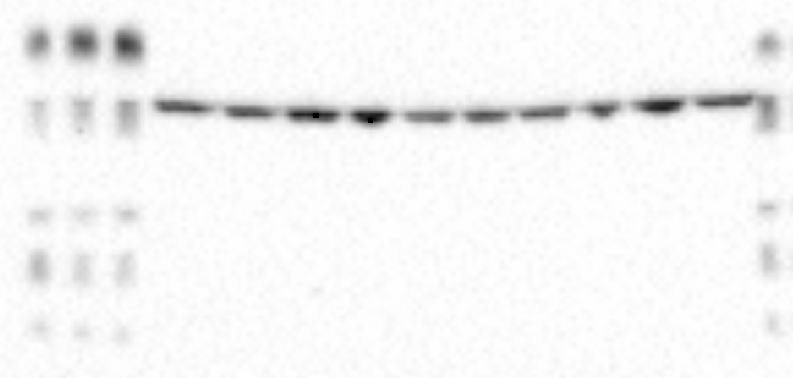

Supplement: Figure 1—figure supplement 1—source data 1. [file elife-82283-fig1-figsupp1-data1.zip › Fig1-SupFig1-Source data/Liver_Gapdh_raw.tif]

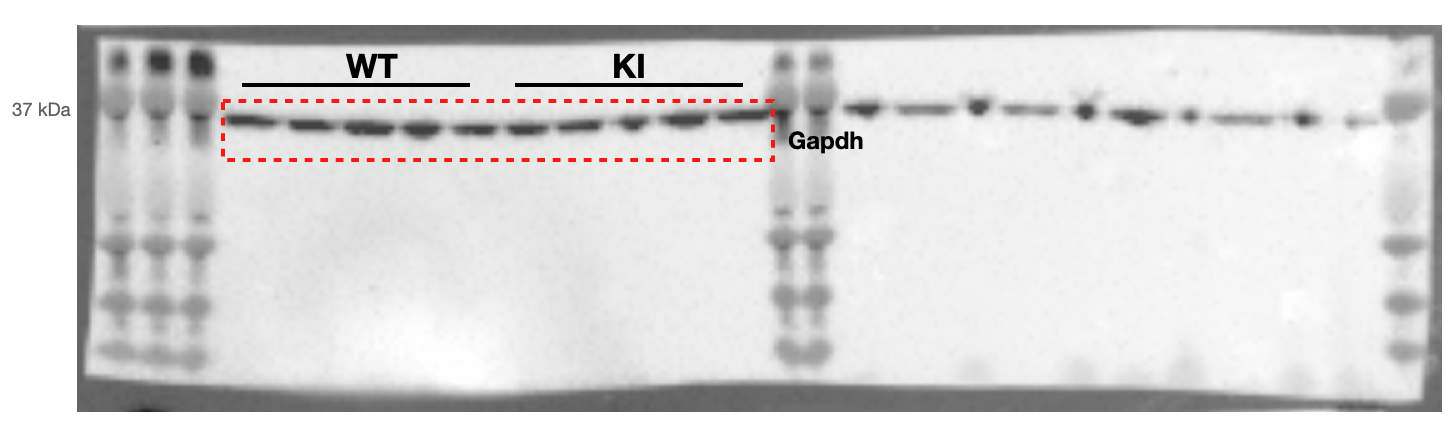

Supplement: Figure 1—figure supplement 1—source data 1. [file elife-82283-fig1-figsupp1-data1.zip › Fig1-SupFig1-Source data/Liver_Gapdh_annotated.png]

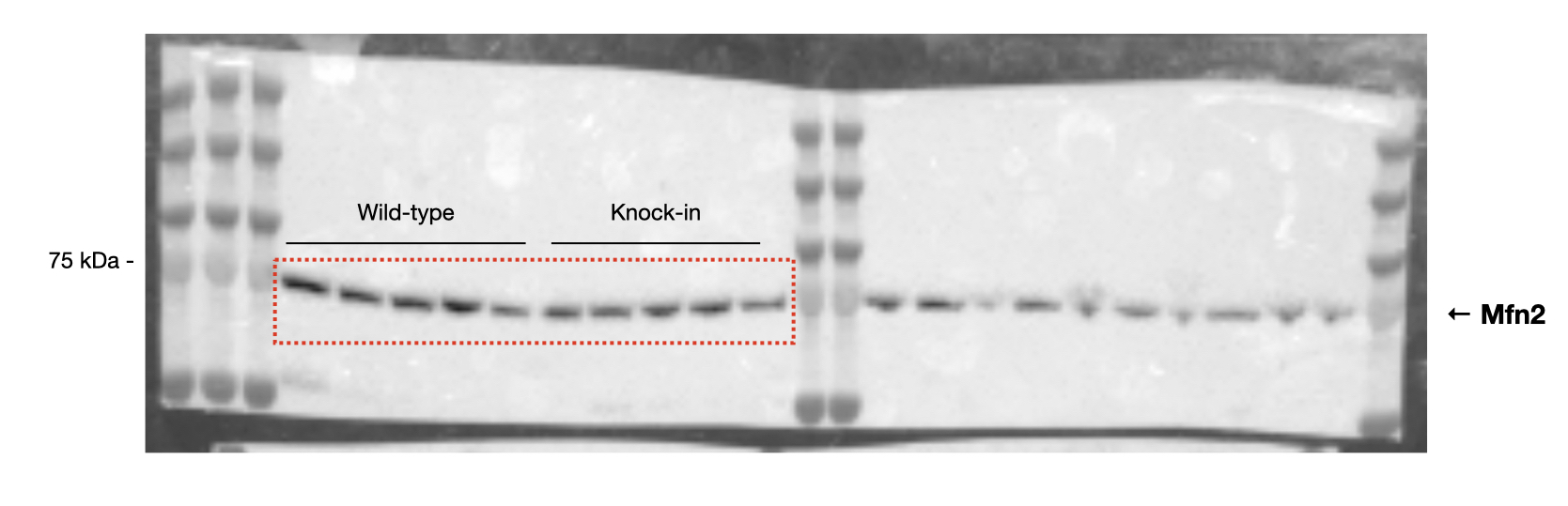

Supplement: Figure 1—figure supplement 1—source data 1. [file elife-82283-fig1-figsupp1-data1.zip › Fig1-SupFig1-Source data/Liver_Mfn2_annotated.jpeg]

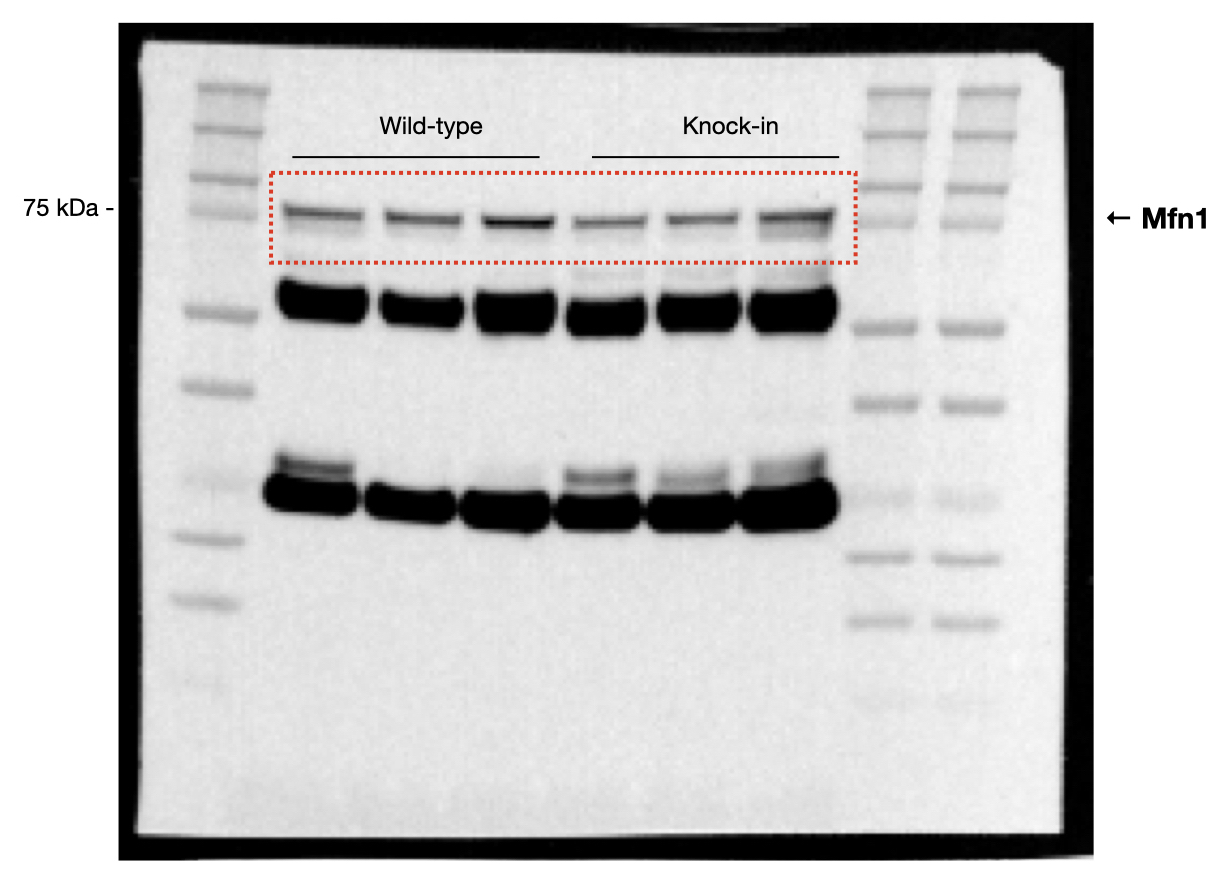

Supplement: Figure 1—figure supplement 1—source data 1. [file elife-82283-fig1-figsupp1-data1.zip › Fig1-SupFig1-Source data/IngWAT_Mfn1.jpeg]

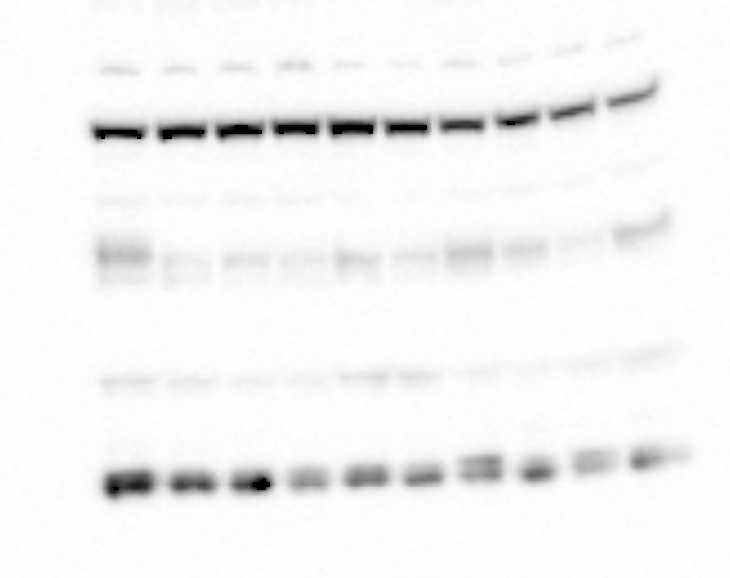

Supplement: Figure 1—figure supplement 1—source data 1. [file elife-82283-fig1-figsupp1-data1.zip › Fig1-SupFig1-Source data/Liver_Mfn1_raw.tif]

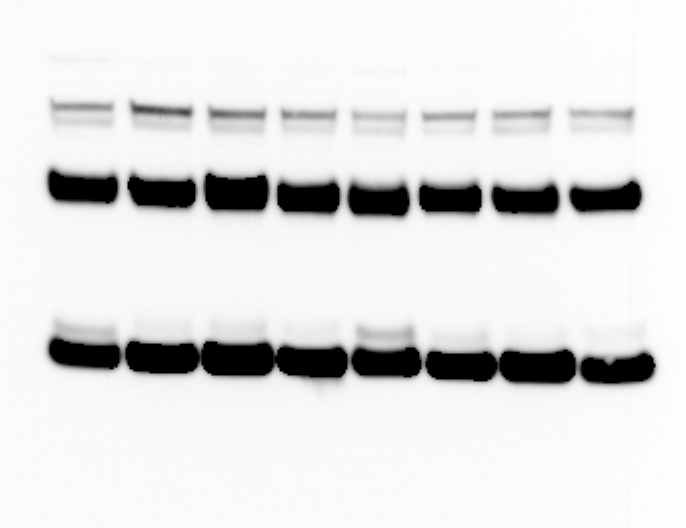

Supplement: Figure 1—figure supplement 1—source data 1. [file elife-82283-fig1-figsupp1-data1.zip › Fig1-SupFig1-Source data/EpiWAT_Mfn1_raw.tif]

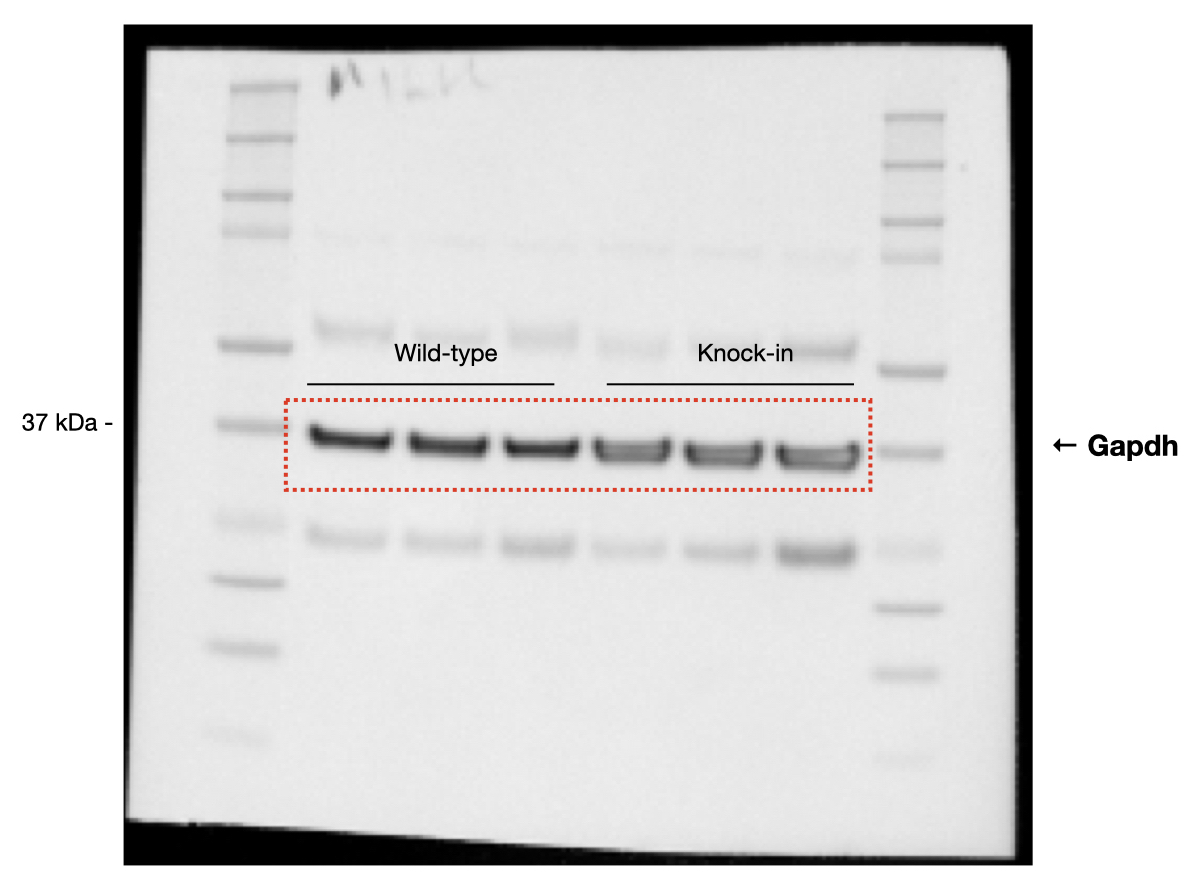

Supplement: Figure 1—figure supplement 1—source data 1. [file elife-82283-fig1-figsupp1-data1.zip › Fig1-SupFig1-Source data/IngWAT_Gapdh.jpeg]

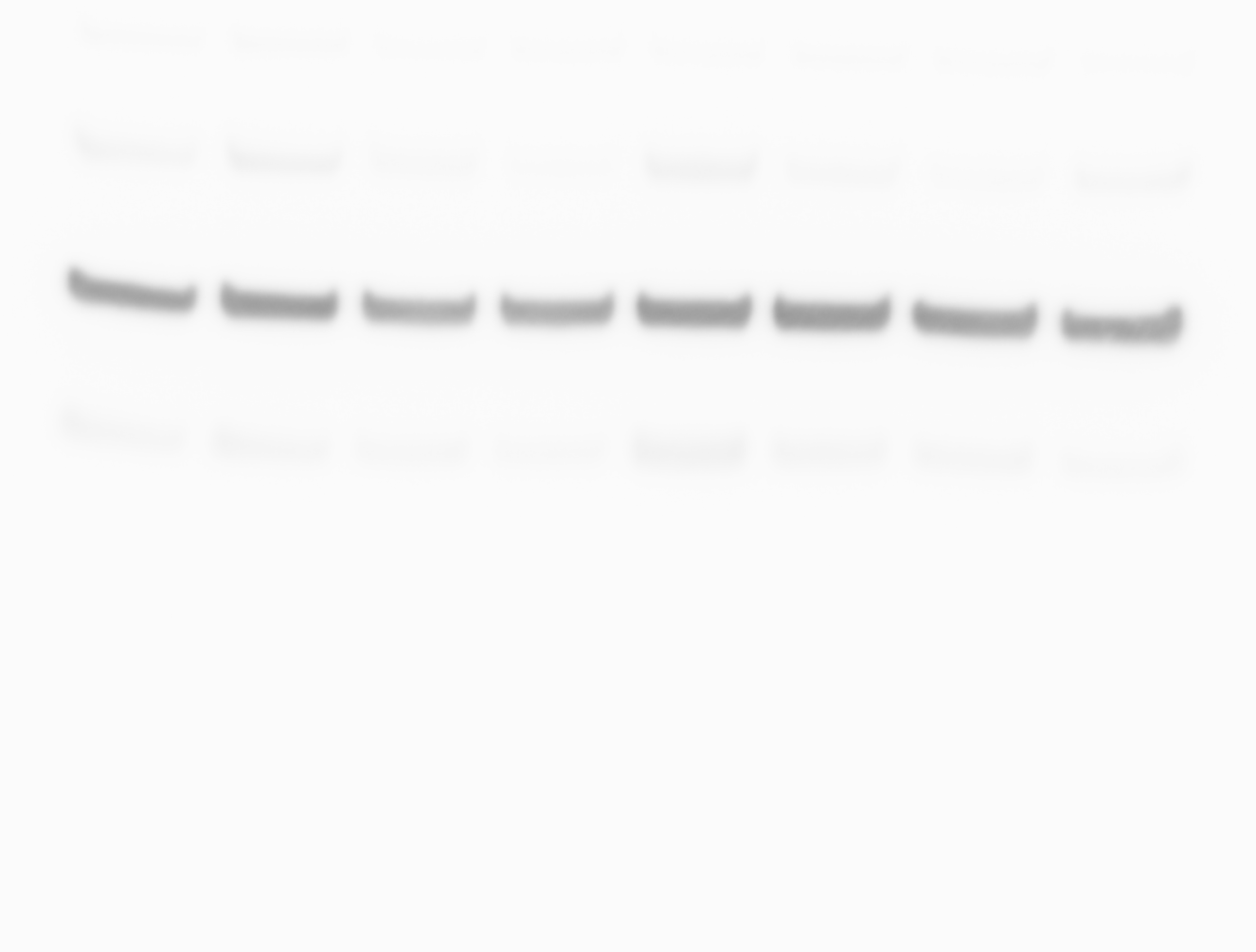

Supplement: Figure 1—figure supplement 1—source data 1. [file elife-82283-fig1-figsupp1-data1.zip › Fig1-SupFig1-Source data/EpiWAT_Gapdh_raw.tif]

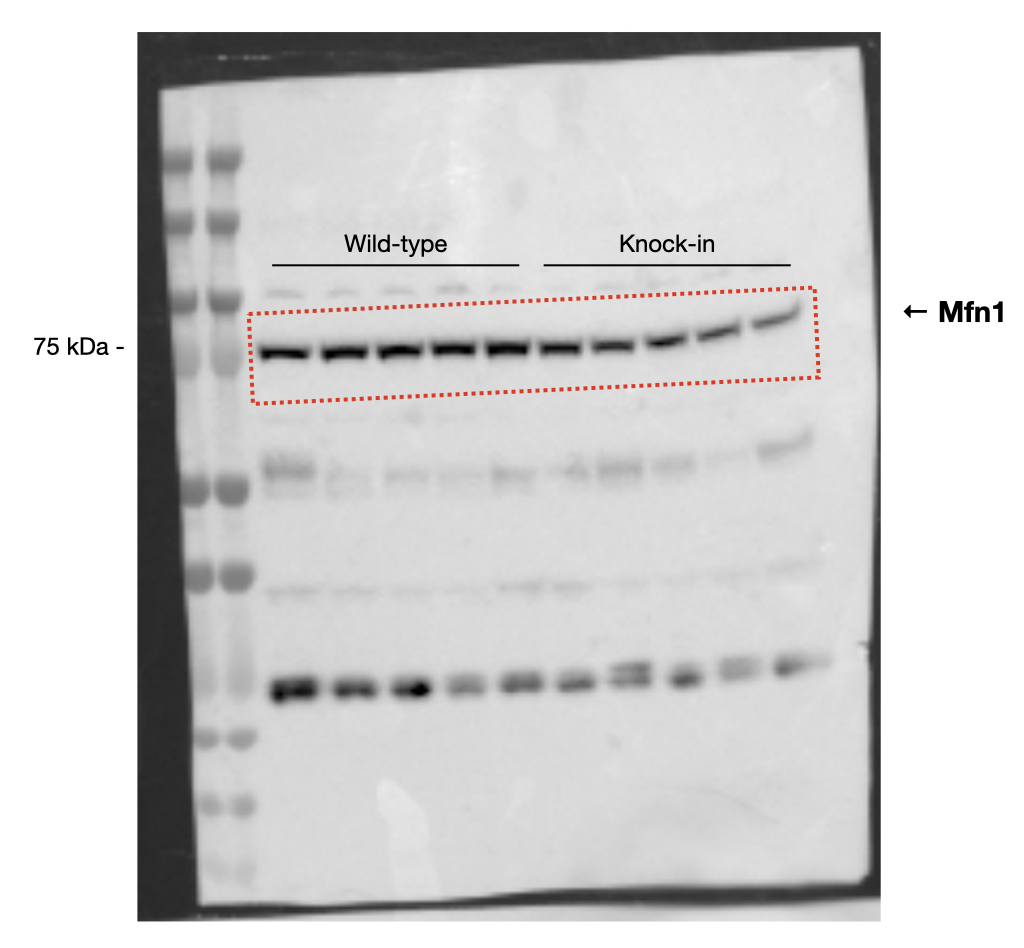

Supplement: Figure 1—figure supplement 1—source data 1. [file elife-82283-fig1-figsupp1-data1.zip › Fig1-SupFig1-Source data/Liver_Mfn1_annotated.jpeg]

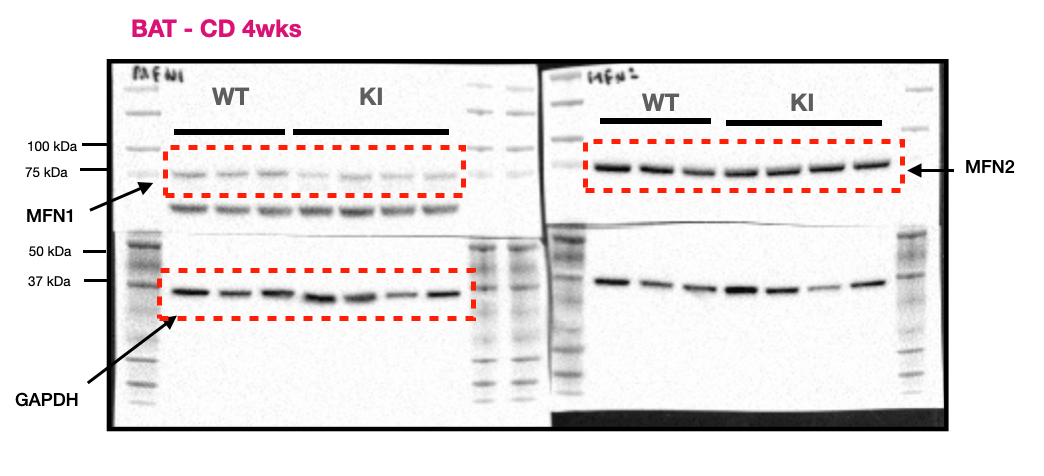

Supplement: Figure 1—figure supplement 1—source data 1. [file elife-82283-fig1-figsupp1-data1.zip › Fig1-SupFig1-Source data/BAT_4weeks.png]

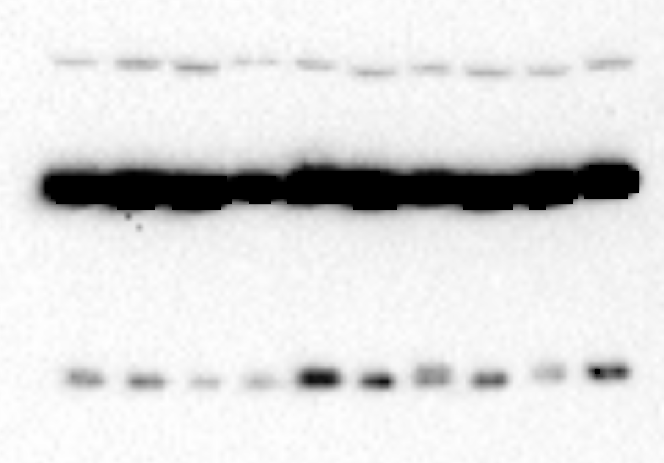

Supplement: Figure 1—figure supplement 1—source data 1. [file elife-82283-fig1-figsupp1-data1.zip › Fig1-SupFig1-Source data/Heart_Mfn1_raw.tif]

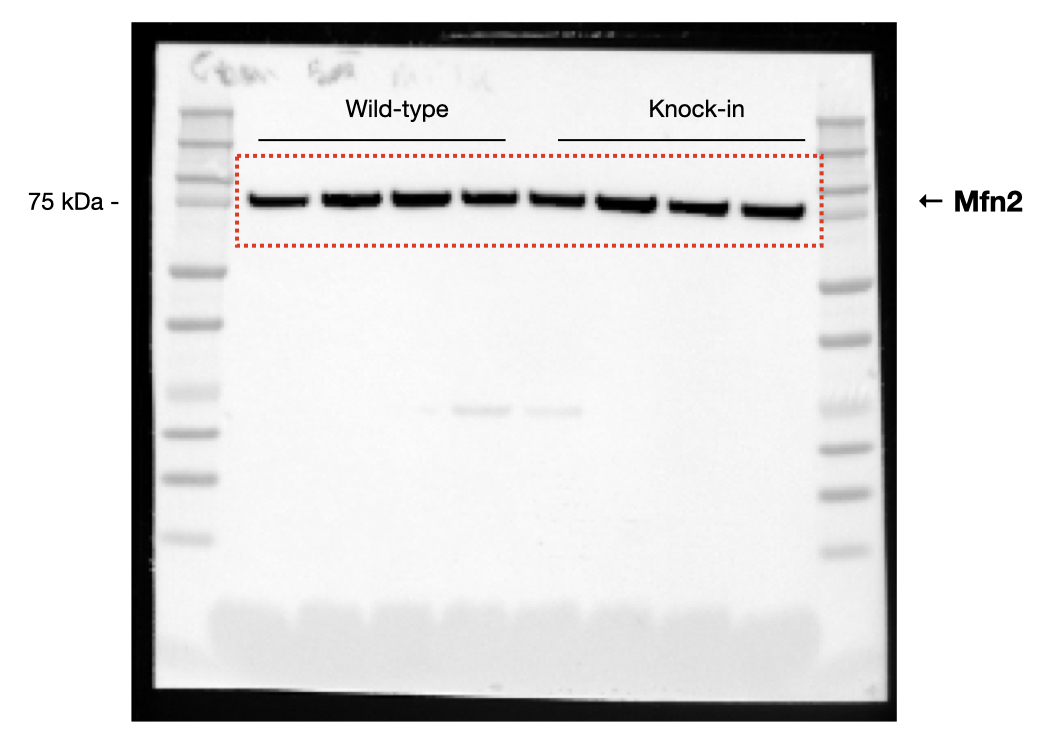

Supplement: Figure 1—figure supplement 1—source data 1. [file elife-82283-fig1-figsupp1-data1.zip › Fig1-SupFig1-Source data/EpiWAT_Mfn2_annotated.jpeg]

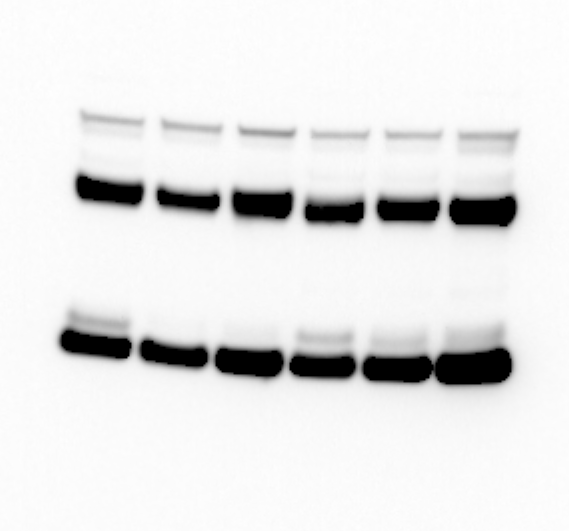

Supplement: Figure 1—figure supplement 1—source data 1. [file elife-82283-fig1-figsupp1-data1.zip › Fig1-SupFig1-Source data/IngWAT_Mfn1_raw.tif]

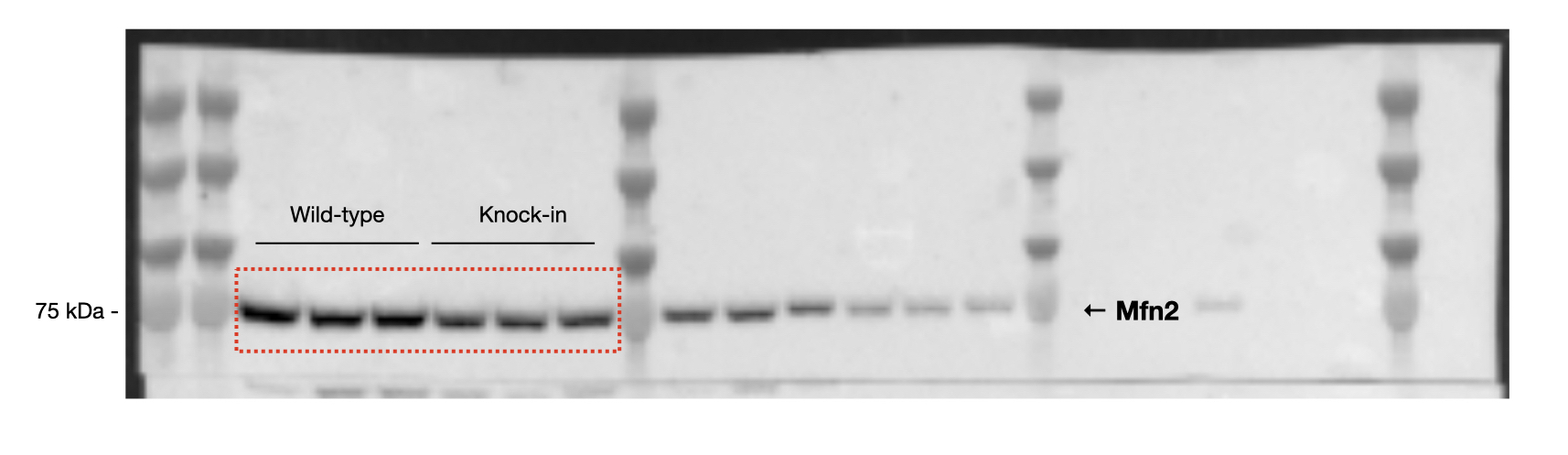

Supplement: Figure 1—figure supplement 1—source data 1. [file elife-82283-fig1-figsupp1-data1.zip › Fig1-SupFig1-Source data/BAT_Mfn2_annotated.jpeg]

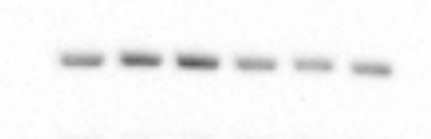

Supplement: Figure 1—figure supplement 1—source data 1. [file elife-82283-fig1-figsupp1-data1.zip › Fig1-SupFig1-Source data/SkelMusc_Mfn2_raw.tif]

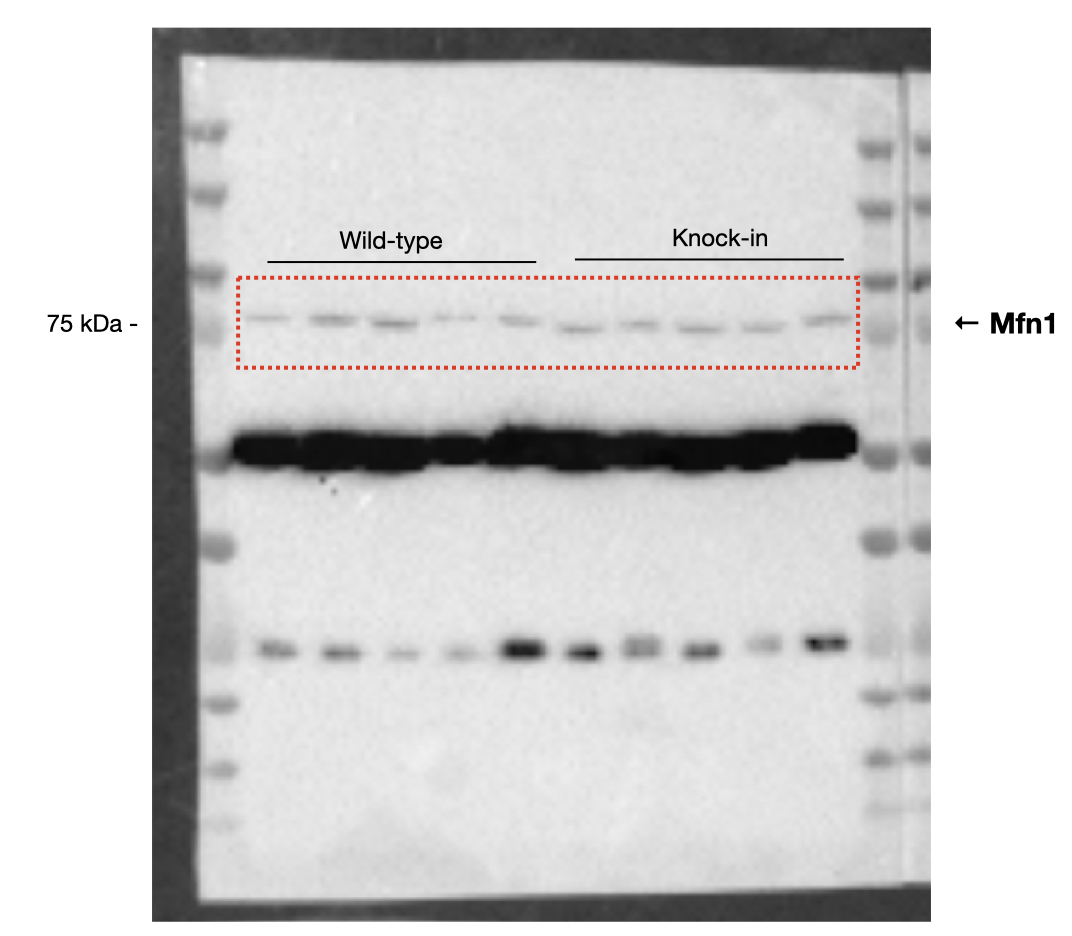

Supplement: Figure 1—figure supplement 1—source data 1. [file elife-82283-fig1-figsupp1-data1.zip › Fig1-SupFig1-Source data/Heart_Mfn1_annotated.jpeg]

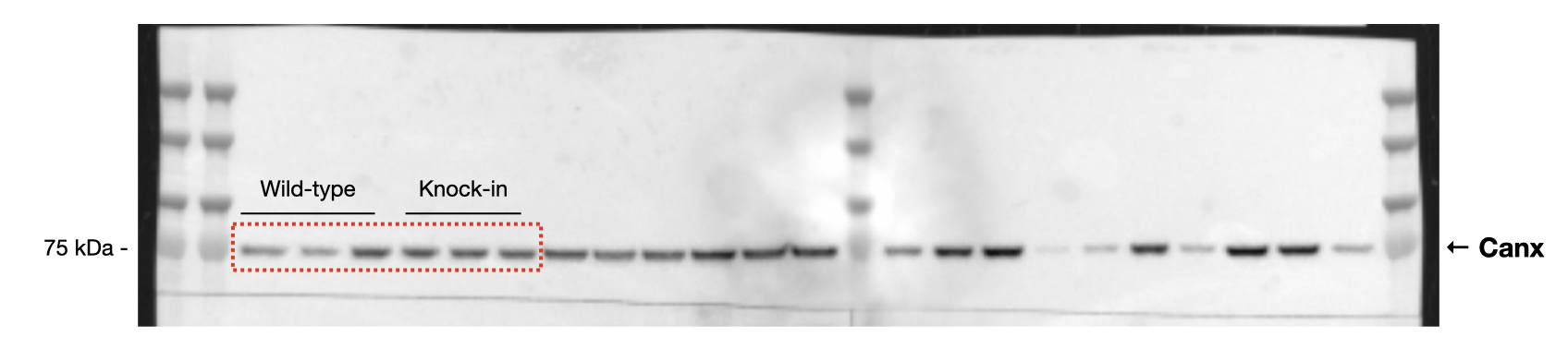

Supplement: Figure 1—figure supplement 1—source data 1. [file elife-82283-fig1-figsupp1-data1.zip › Fig1-SupFig1-Source data/BAT_Canx_annotated.jpeg]

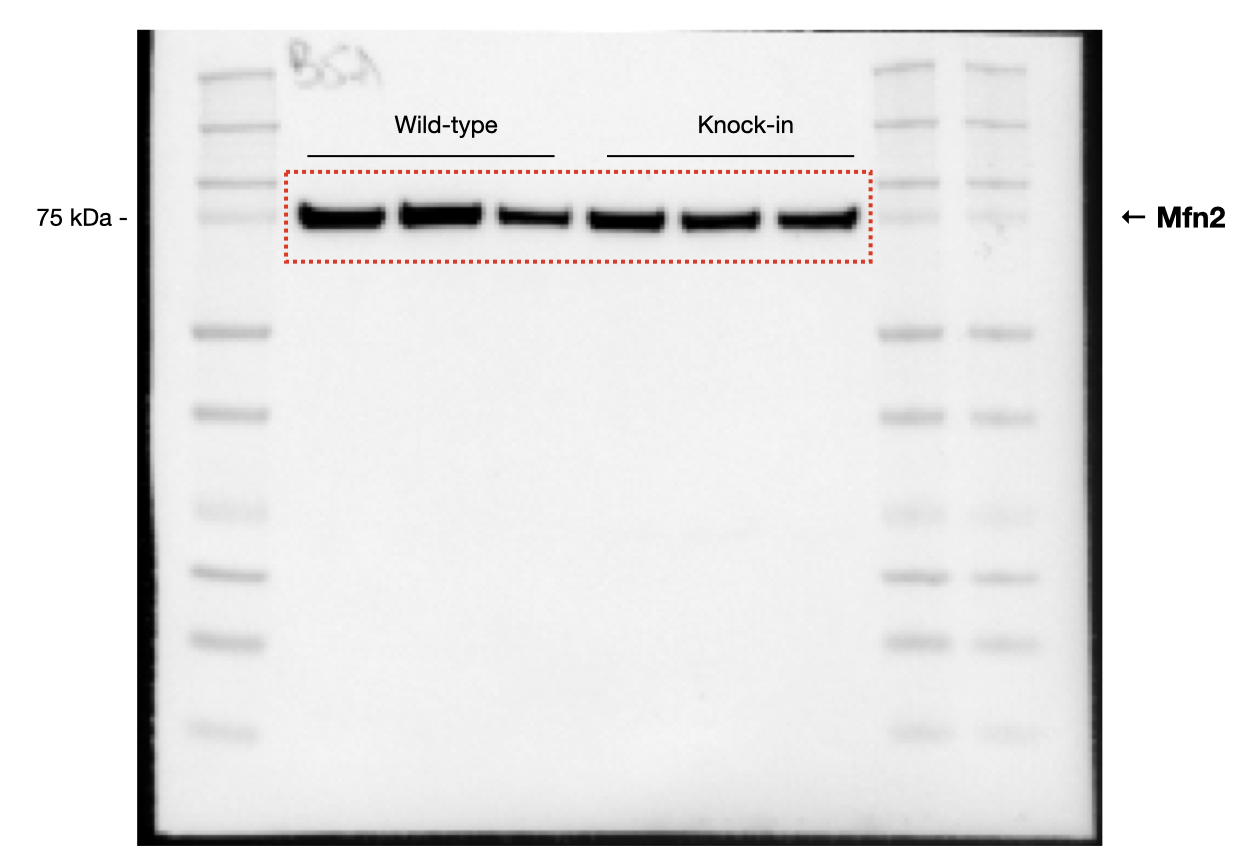

Supplement: Figure 1—figure supplement 1—source data 1. [file elife-82283-fig1-figsupp1-data1.zip › Fig1-SupFig1-Source data/IngWAT_Mfn2.jpeg]

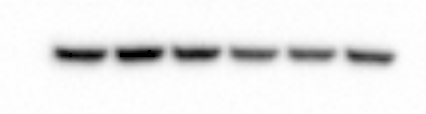

Supplement: Figure 1—figure supplement 1—source data 1. [file elife-82283-fig1-figsupp1-data1.zip › Fig1-SupFig1-Source data/SkelMusc_Tubb_raw.tif]

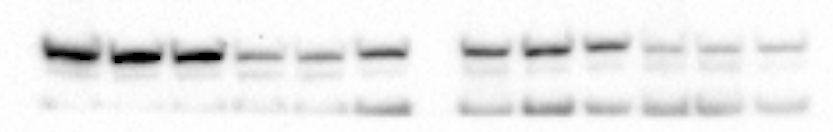

Supplement: Figure 1—figure supplement 1—source data 1. [file elife-82283-fig1-figsupp1-data1.zip › Fig1-SupFig1-Source data/BAT_Mfn1_raw.tif]

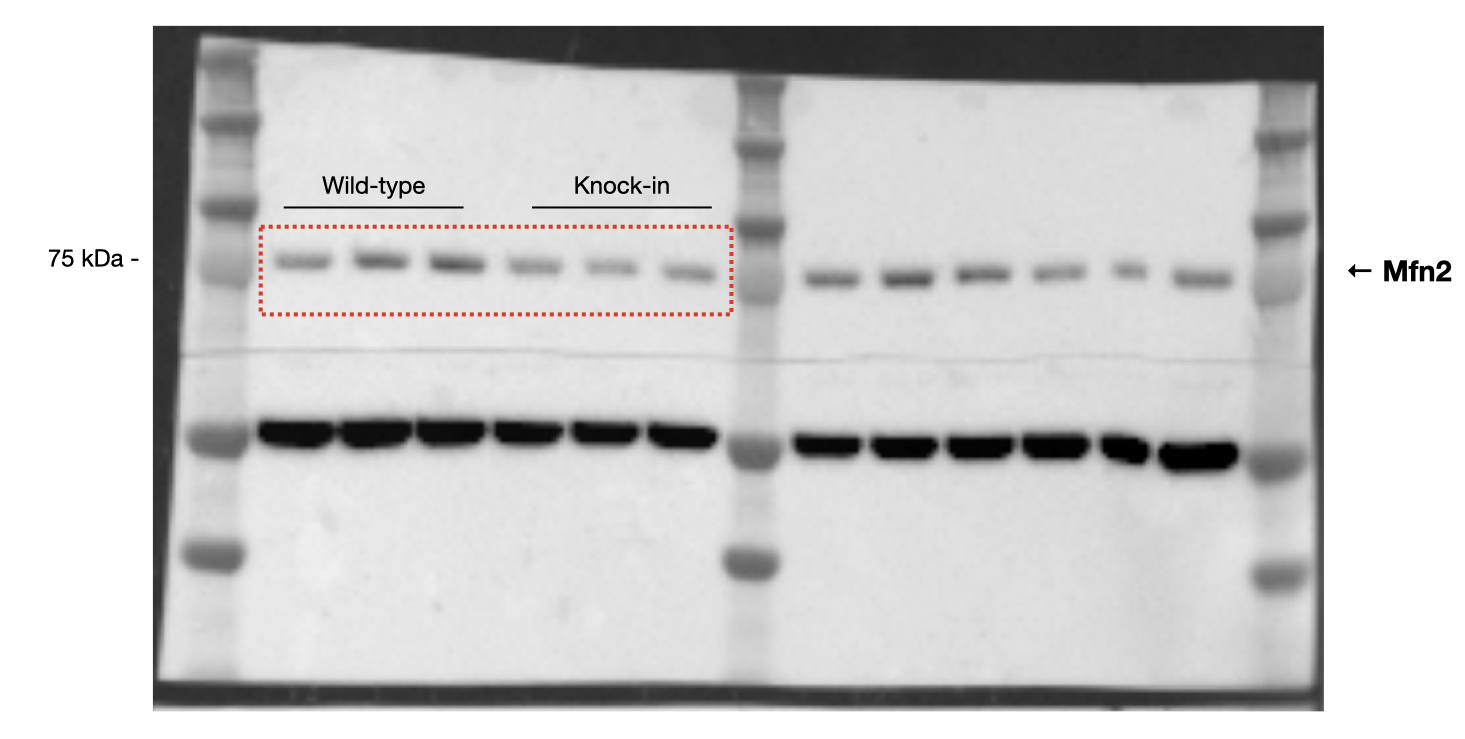

Supplement: Figure 1—figure supplement 1—source data 1. [file elife-82283-fig1-figsupp1-data1.zip › Fig1-SupFig1-Source data/SkelMusc_Mfn2_annotated.jpeg]

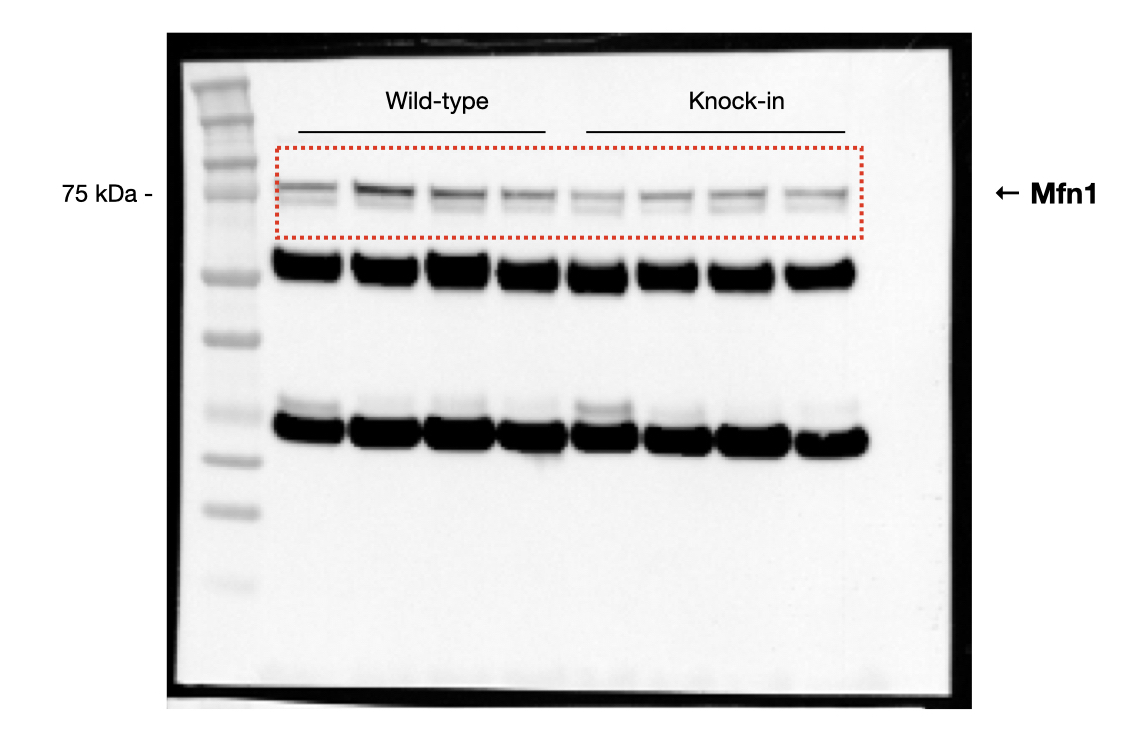

Supplement: Figure 1—figure supplement 1—source data 1. [file elife-82283-fig1-figsupp1-data1.zip › Fig1-SupFig1-Source data/EpiWAT_Mfn1_annotated.jpeg]

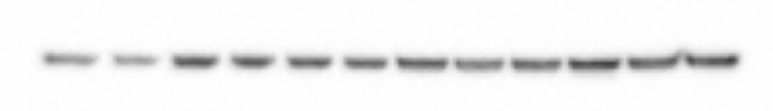

Supplement: Figure 1—figure supplement 1—source data 1. [file elife-82283-fig1-figsupp1-data1.zip › Fig1-SupFig1-Source data/BAT_Canx_raw.tif]

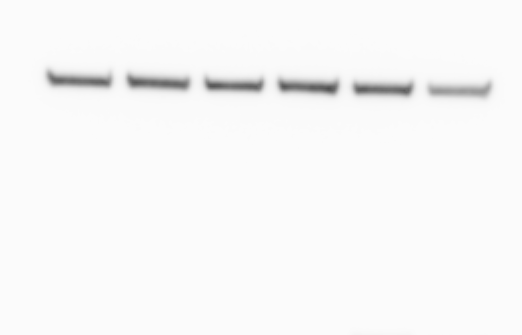

Supplement: Figure 1—figure supplement 1—source data 1. [file elife-82283-fig1-figsupp1-data1.zip › Fig1-SupFig1-Source data/IngWAT_Mfn2_raw.tif]

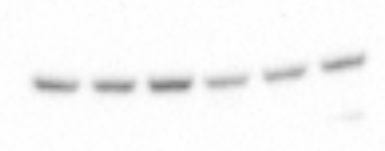

Supplement: Figure 1—figure supplement 1—source data 1. [file elife-82283-fig1-figsupp1-data1.zip › Fig1-SupFig1-Source data/SkelMusc_Mfn1_raw.tif]

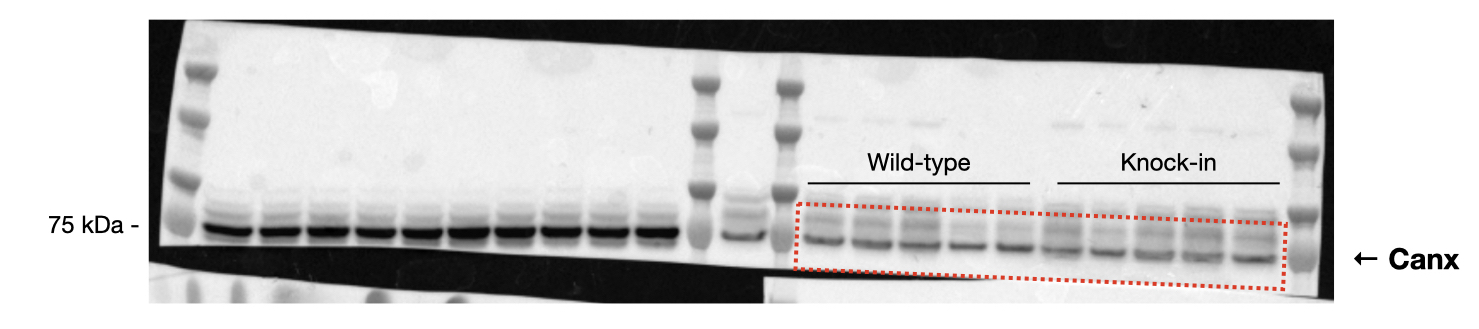

Supplement: Figure 1—figure supplement 1—source data 1. [file elife-82283-fig1-figsupp1-data1.zip › Fig1-SupFig1-Source data/Heart_Canx_annotated.jpeg]

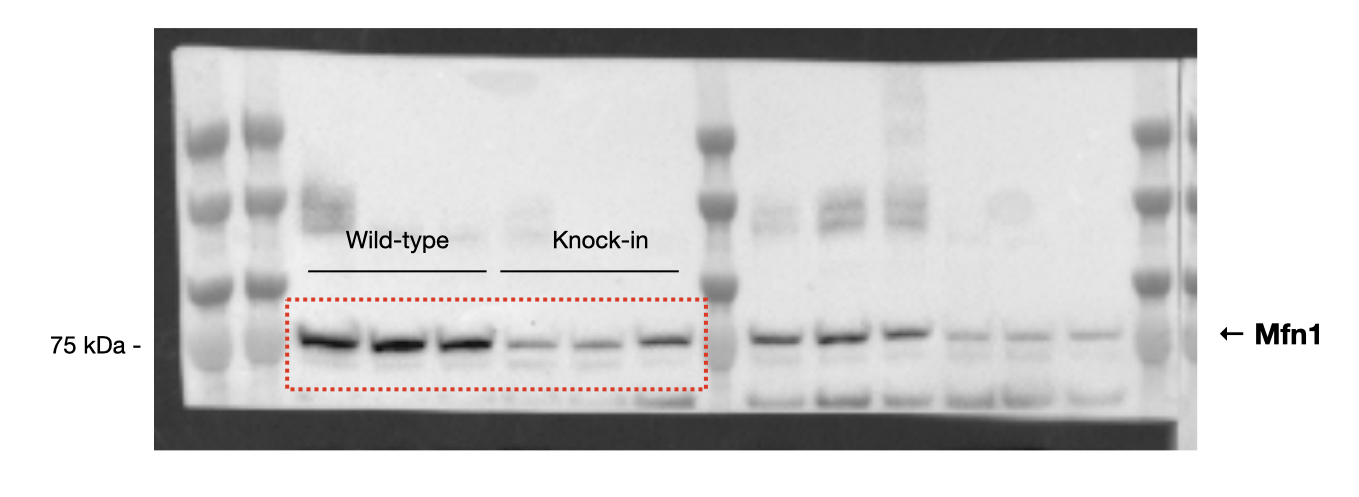

Supplement: Figure 1—figure supplement 1—source data 1. [file elife-82283-fig1-figsupp1-data1.zip › Fig1-SupFig1-Source data/BAT_Mfn1_annotated.jpeg]

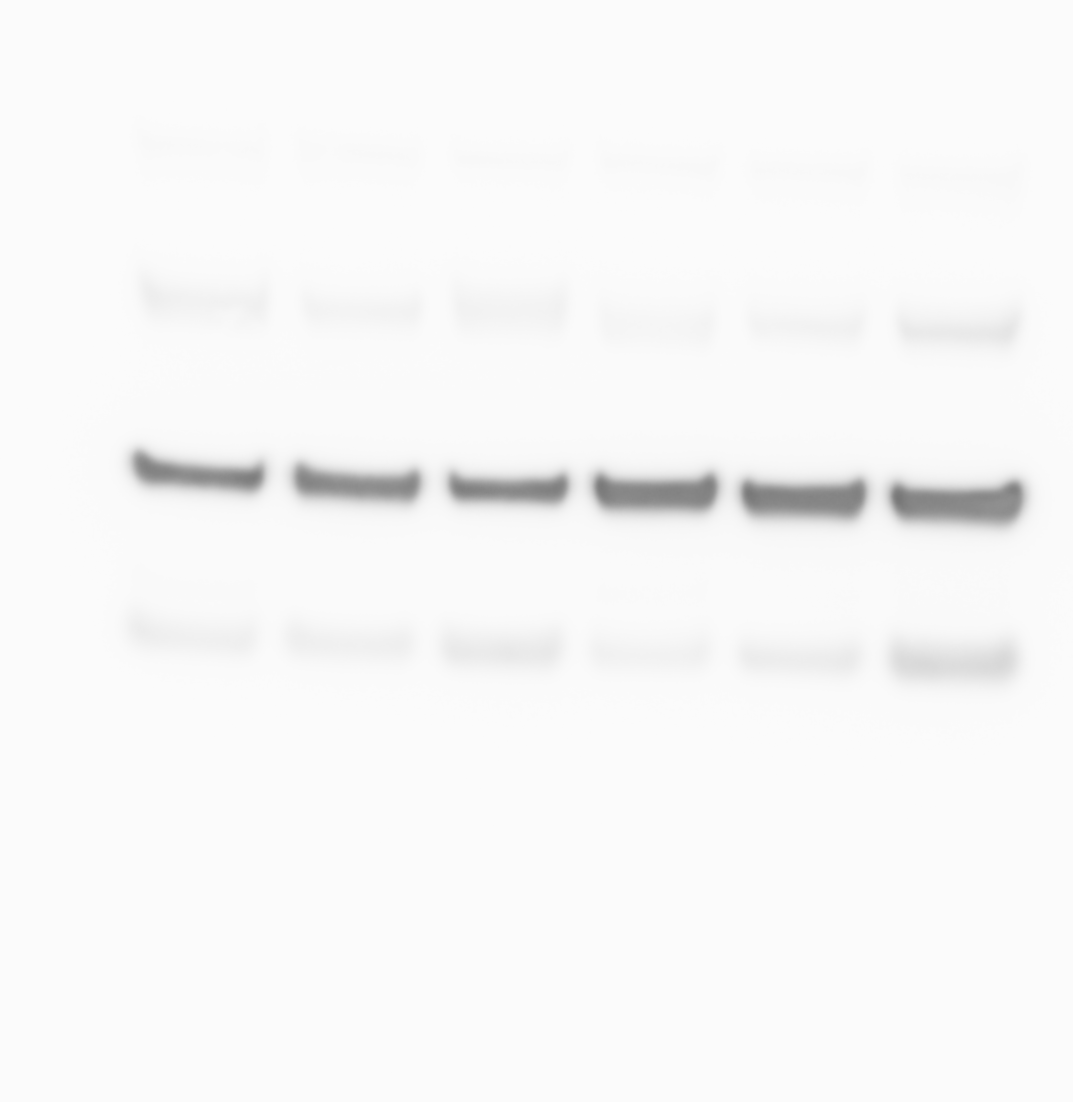

Supplement: Figure 1—figure supplement 1—source data 1. [file elife-82283-fig1-figsupp1-data1.zip › Fig1-SupFig1-Source data/IngWAT_Gapdh_raw.tif]

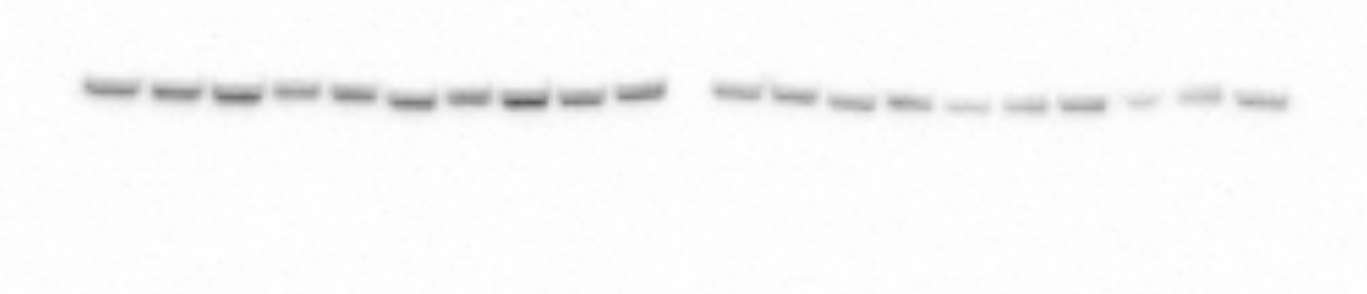

Supplement: Figure 1—figure supplement 1—source data 1. [file elife-82283-fig1-figsupp1-data1.zip › Fig1-SupFig1-Source data/Heart_Mfn2_raw.tif]

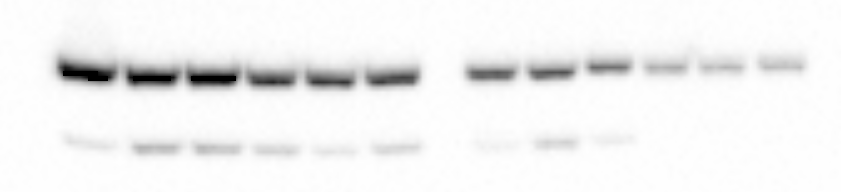

Supplement: Figure 1—figure supplement 1—source data 1. [file elife-82283-fig1-figsupp1-data1.zip › Fig1-SupFig1-Source data/BAT_Mfn2_raw.tif]

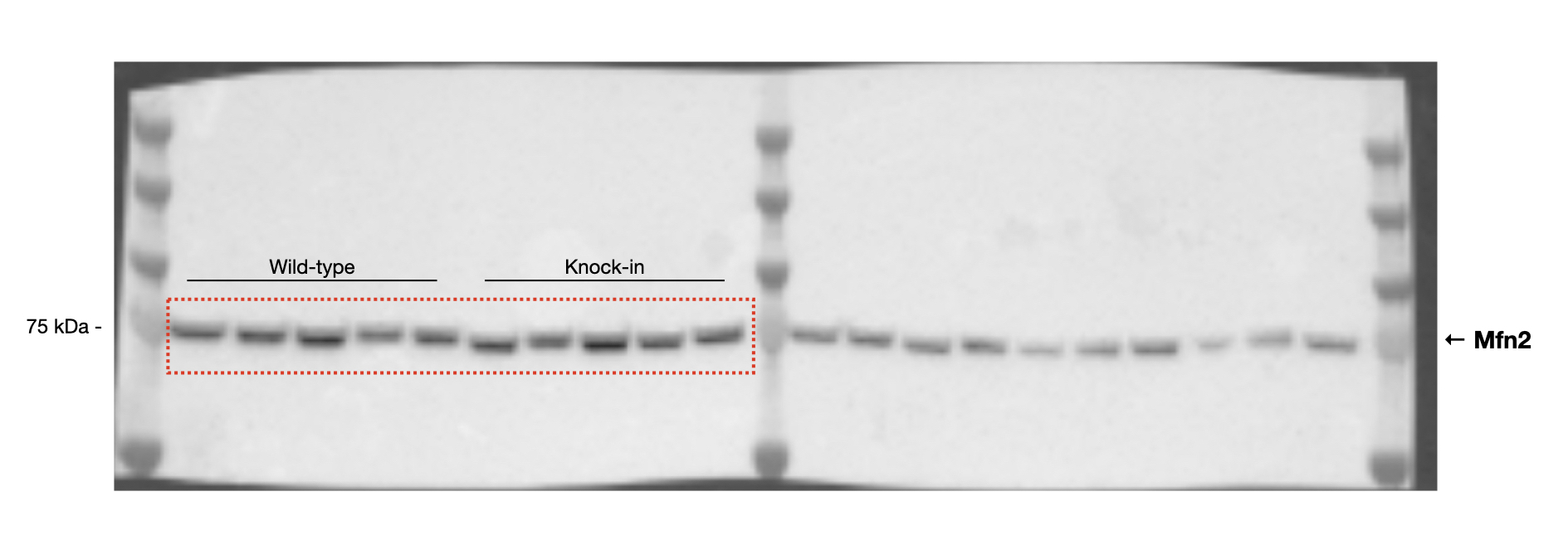

Supplement: Figure 1—figure supplement 1—source data 1. [file elife-82283-fig1-figsupp1-data1.zip › Fig1-SupFig1-Source data/Heart_Mfn2_annotated.jpeg]

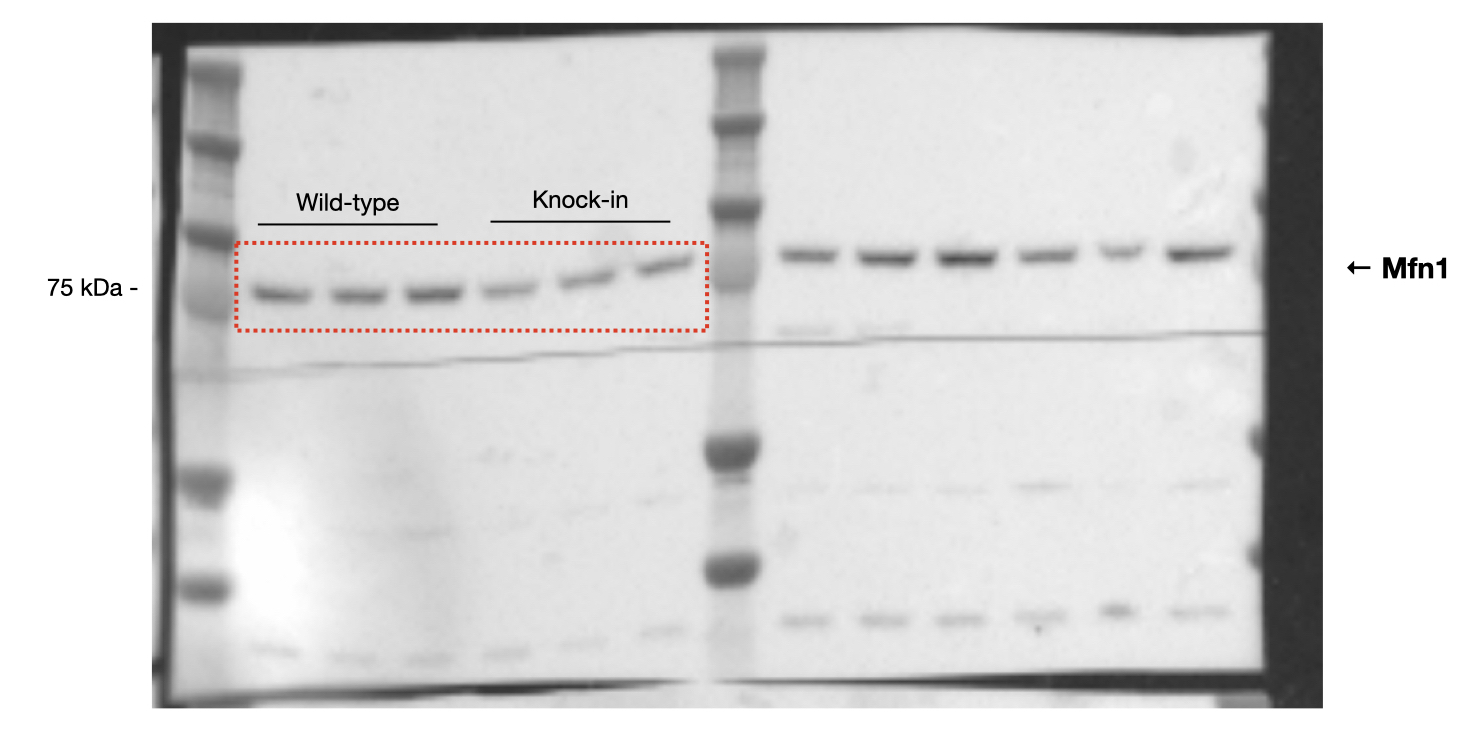

Supplement: Figure 1—figure supplement 1—source data 1. [file elife-82283-fig1-figsupp1-data1.zip › Fig1-SupFig1-Source data/SkelMusc_Mfn1_annotated.jpeg]

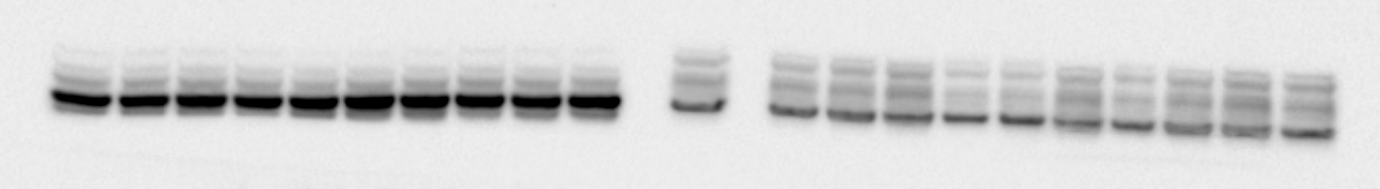

Supplement: Figure 1—figure supplement 1—source data 1. [file elife-82283-fig1-figsupp1-data1.zip › Fig1-SupFig1-Source data/Heart_Canx_raw.tif]

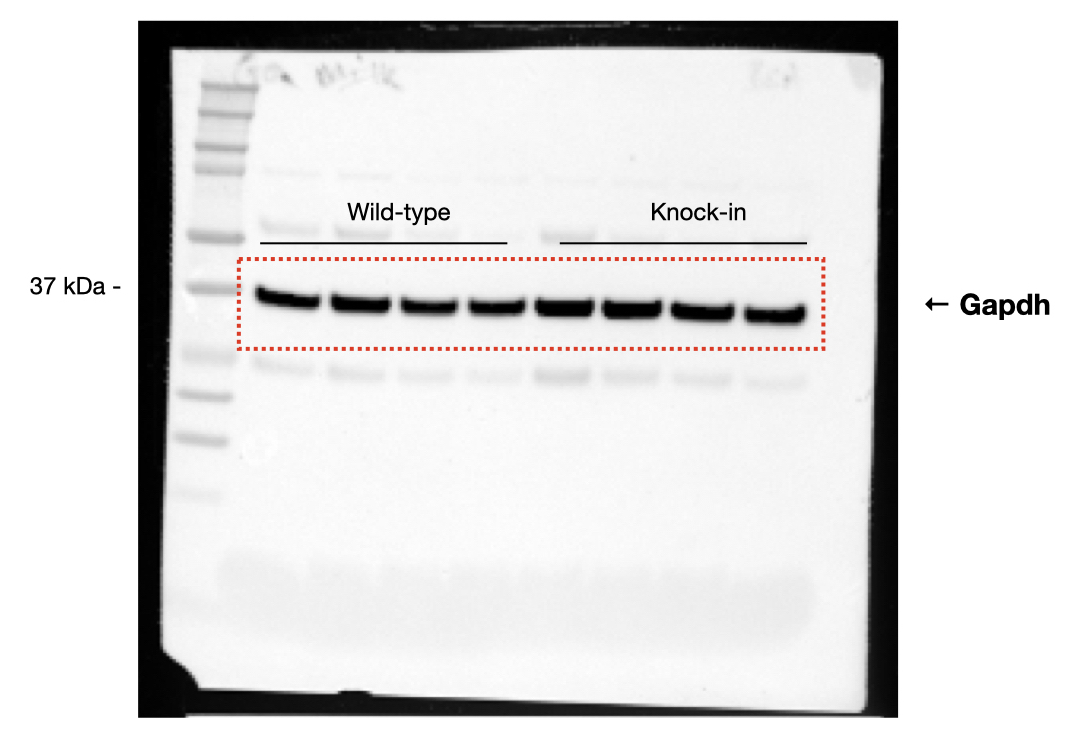

Supplement: Figure 1—figure supplement 1—source data 1. [file elife-82283-fig1-figsupp1-data1.zip › Fig1-SupFig1-Source data/EpiWAT_Gapdh_annotated.jpeg]

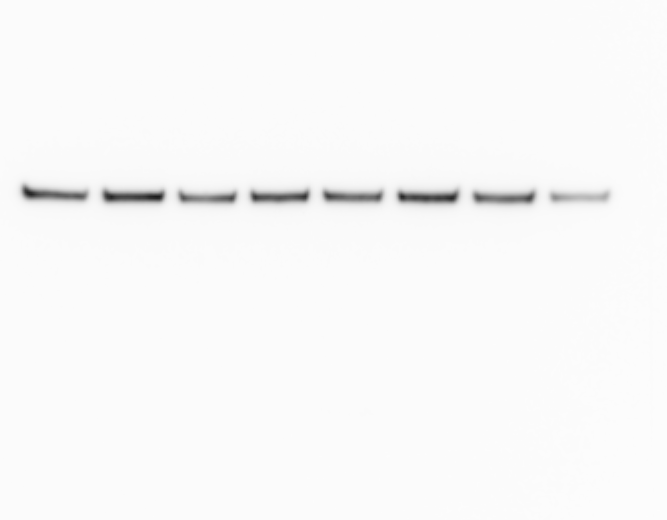

Supplement: Figure 1—figure supplement 3—source data 1. [file elife-82283-fig1-figsupp3-data1.zip › Fig1-SupFig3-Source data/EpiWAT_Mfn2_raw.tif]

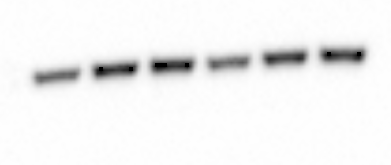

Supplement: Figure 1—figure supplement 3—source data 1. [file elife-82283-fig1-figsupp3-data1.zip › Fig1-SupFig3-Source data/Liver_Mfn2_raw.tif]

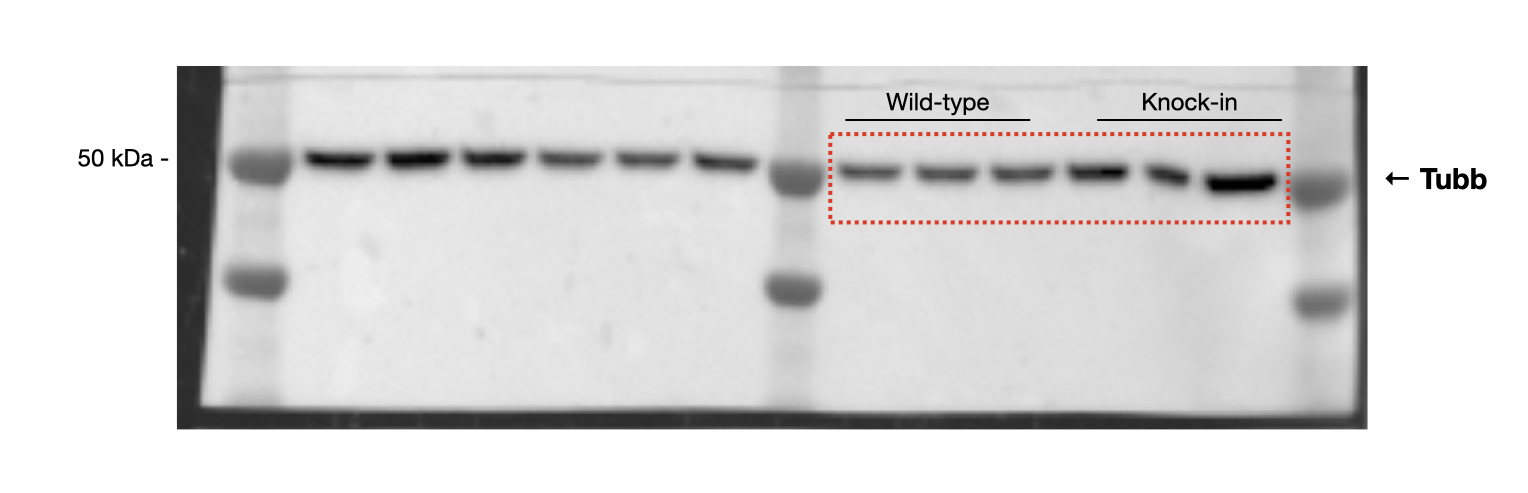

Supplement: Figure 1—figure supplement 3—source data 1. [file elife-82283-fig1-figsupp3-data1.zip › Fig1-SupFig3-Source data/SkelMusc_Tubb_annotated.jpeg]

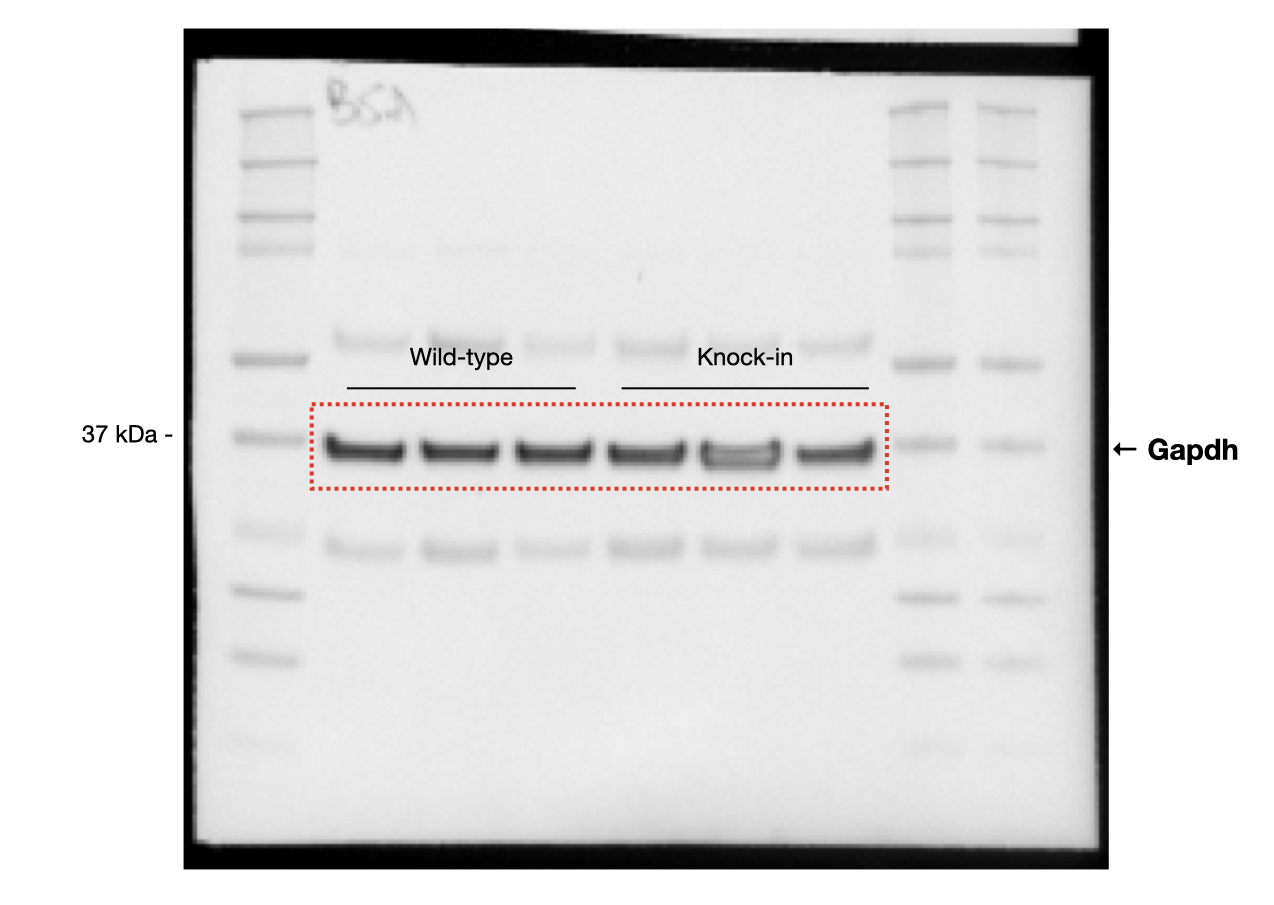

Supplement: Figure 1—figure supplement 3—source data 1. [file elife-82283-fig1-figsupp3-data1.zip › Fig1-SupFig3-Source data/IngWAT_Gapdh_annotated.jpeg]

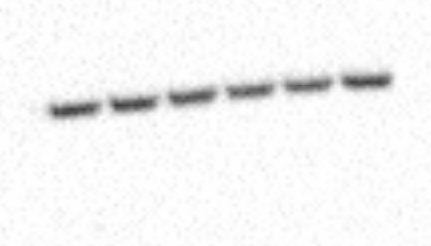

Supplement: Figure 1—figure supplement 3—source data 1. [file elife-82283-fig1-figsupp3-data1.zip › Fig1-SupFig3-Source data/Liver_Tubb_raw.tif]

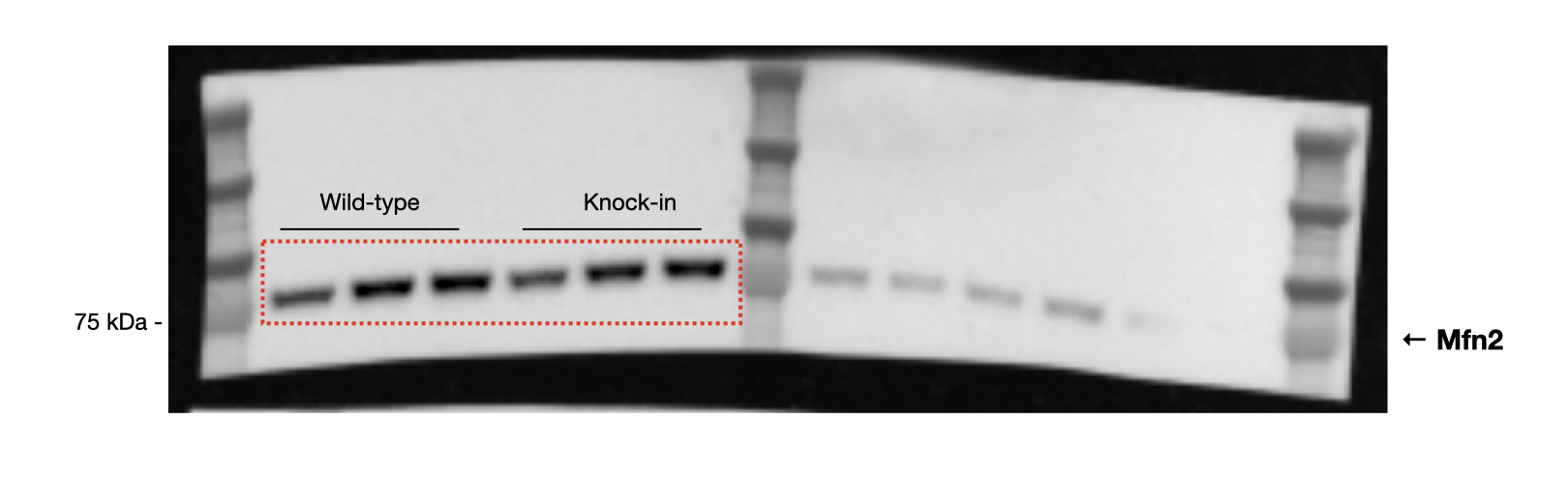

Supplement: Figure 1—figure supplement 3—source data 1. [file elife-82283-fig1-figsupp3-data1.zip › Fig1-SupFig3-Source data/Liver_Mfn2_annotated.jpeg]

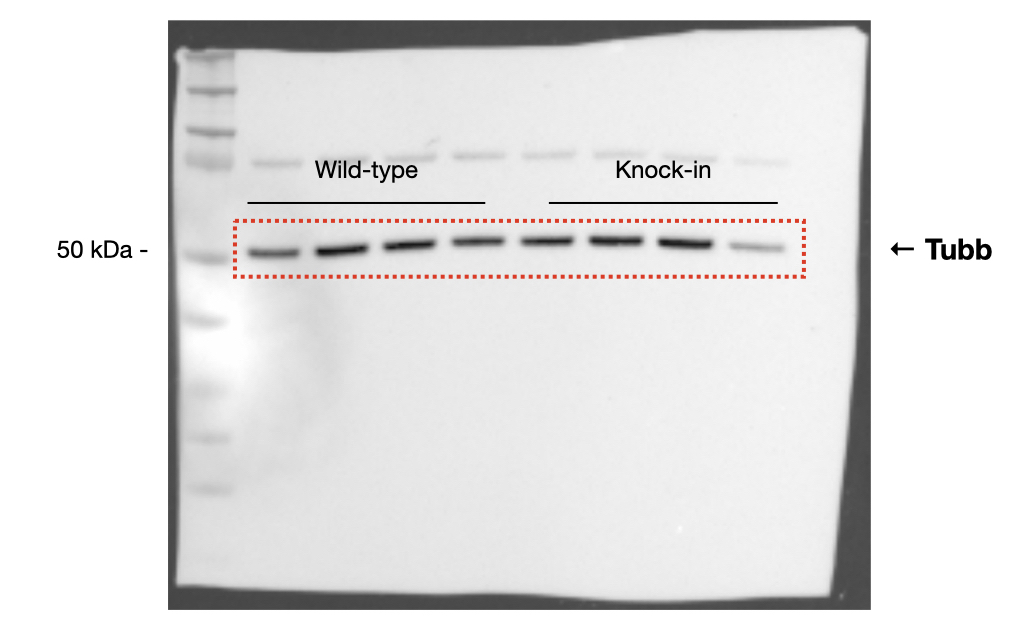

Supplement: Figure 1—figure supplement 3—source data 1. [file elife-82283-fig1-figsupp3-data1.zip › Fig1-SupFig3-Source data/BAT_Tubb_annotated.jpeg]

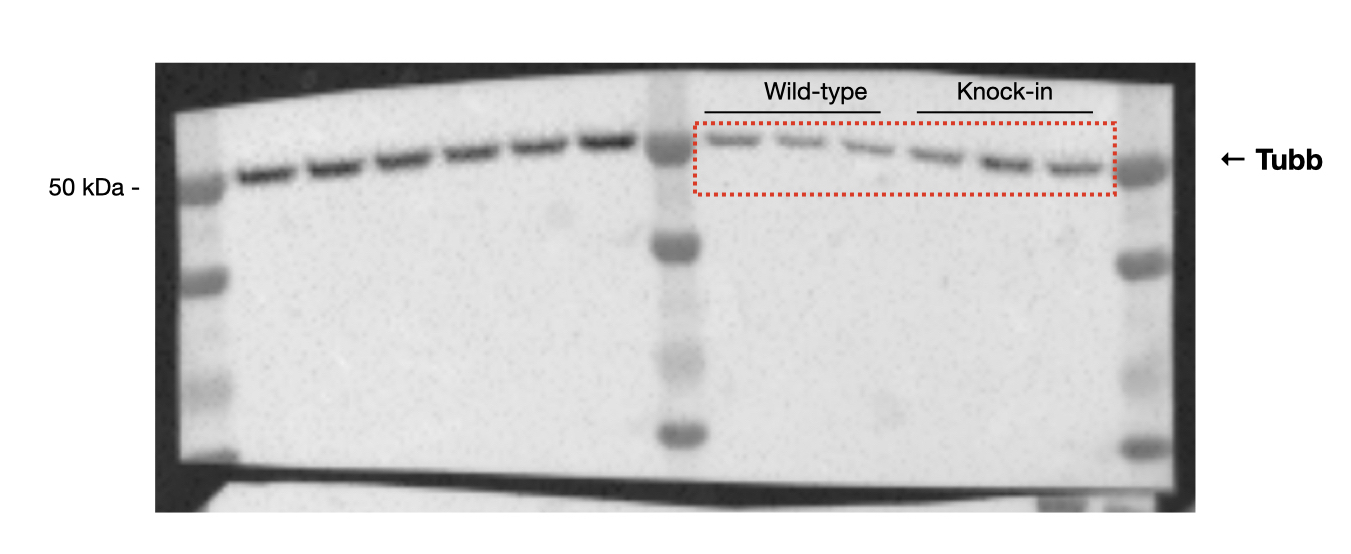

Supplement: Figure 1—figure supplement 3—source data 1. [file elife-82283-fig1-figsupp3-data1.zip › Fig1-SupFig3-Source data/Heart_Tubb_annotated.jpeg]

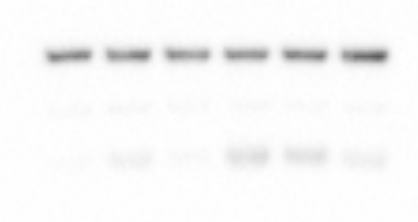

Supplement: Figure 1—figure supplement 3—source data 1. [file elife-82283-fig1-figsupp3-data1.zip › Fig1-SupFig3-Source data/Liver_Mfn1_raw.tif]

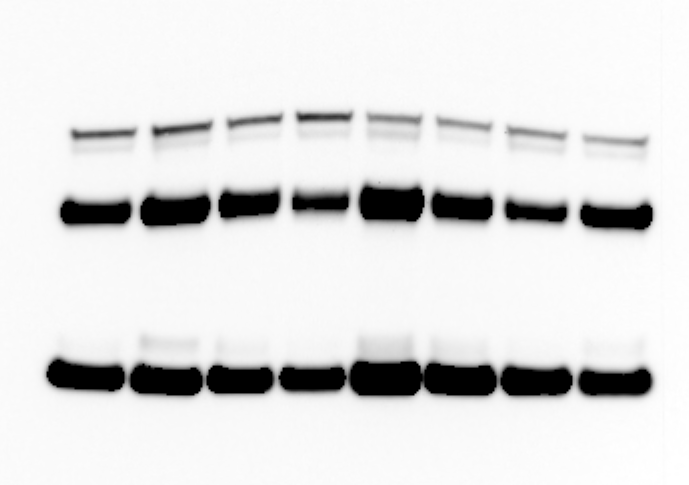

Supplement: Figure 1—figure supplement 3—source data 1. [file elife-82283-fig1-figsupp3-data1.zip › Fig1-SupFig3-Source data/EpiWAT_Mfn1_raw.tif]

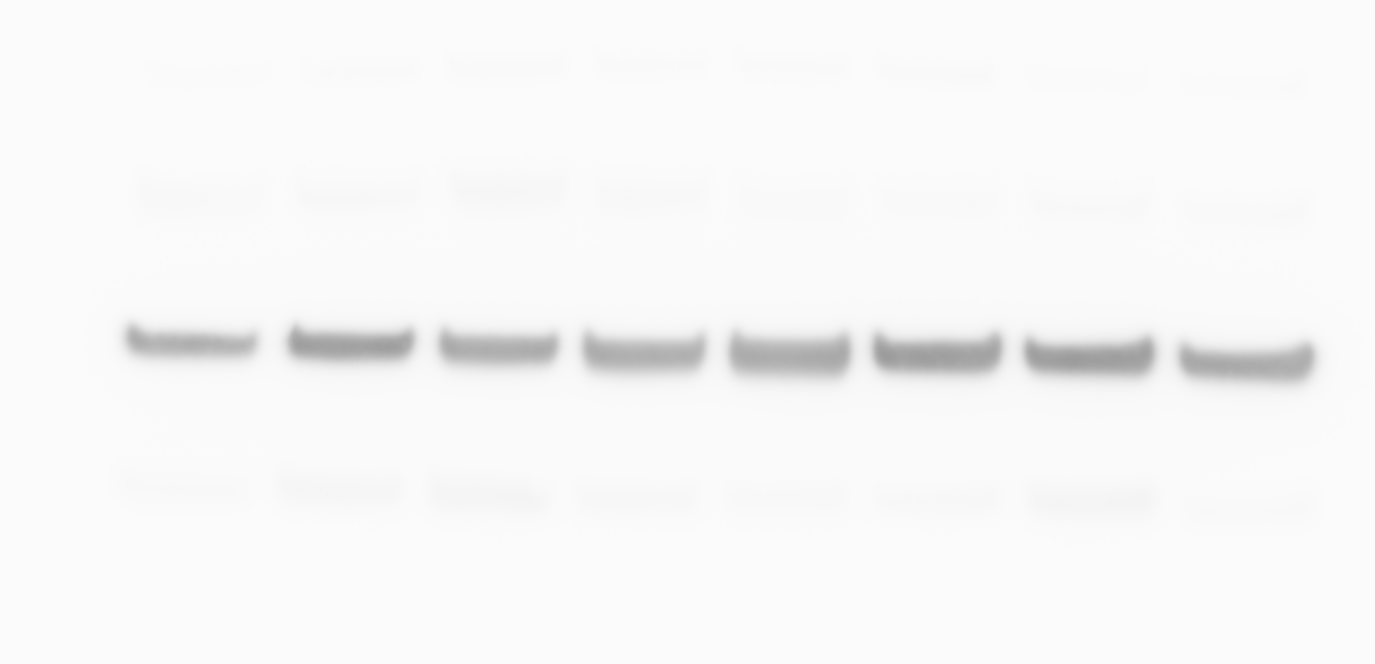

Supplement: Figure 1—figure supplement 3—source data 1. [file elife-82283-fig1-figsupp3-data1.zip › Fig1-SupFig3-Source data/EpiWAT_Gapdh_raw.tif]

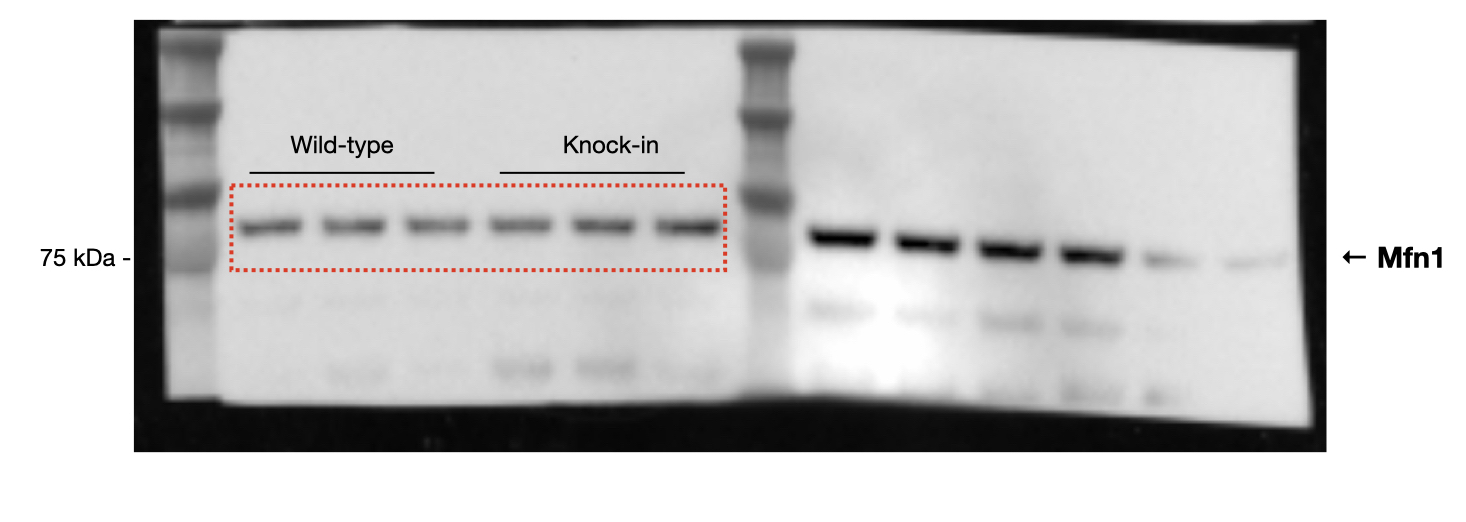

Supplement: Figure 1—figure supplement 3—source data 1. [file elife-82283-fig1-figsupp3-data1.zip › Fig1-SupFig3-Source data/Liver_Mfn1_annotated.jpeg]

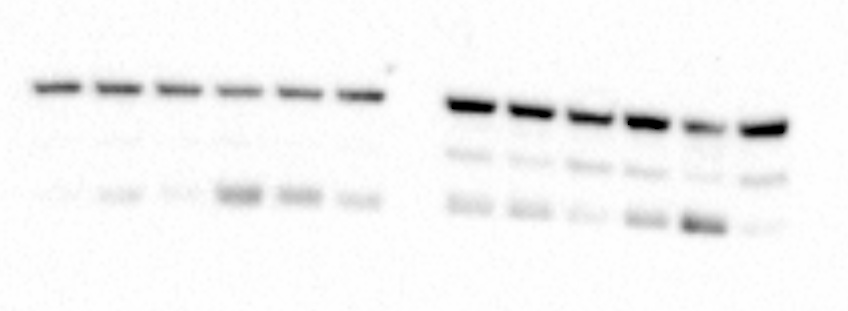

Supplement: Figure 1—figure supplement 3—source data 1. [file elife-82283-fig1-figsupp3-data1.zip › Fig1-SupFig3-Source data/Heart_Mfn1_raw.tif]

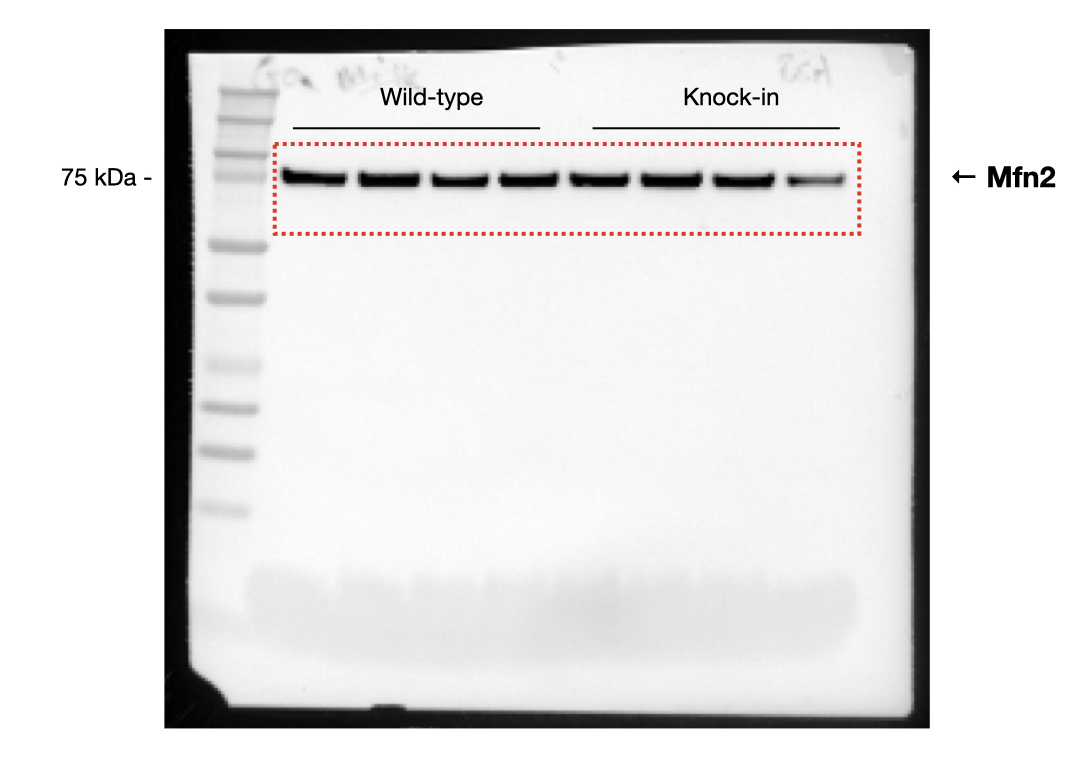

Supplement: Figure 1—figure supplement 3—source data 1. [file elife-82283-fig1-figsupp3-data1.zip › Fig1-SupFig3-Source data/EpiWAT_Mfn2_annotated.jpeg]

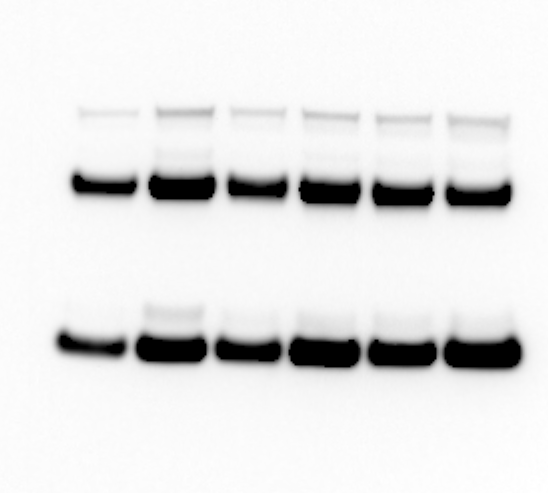

Supplement: Figure 1—figure supplement 3—source data 1. [file elife-82283-fig1-figsupp3-data1.zip › Fig1-SupFig3-Source data/IngWAT_Mfn1_raw.tif]

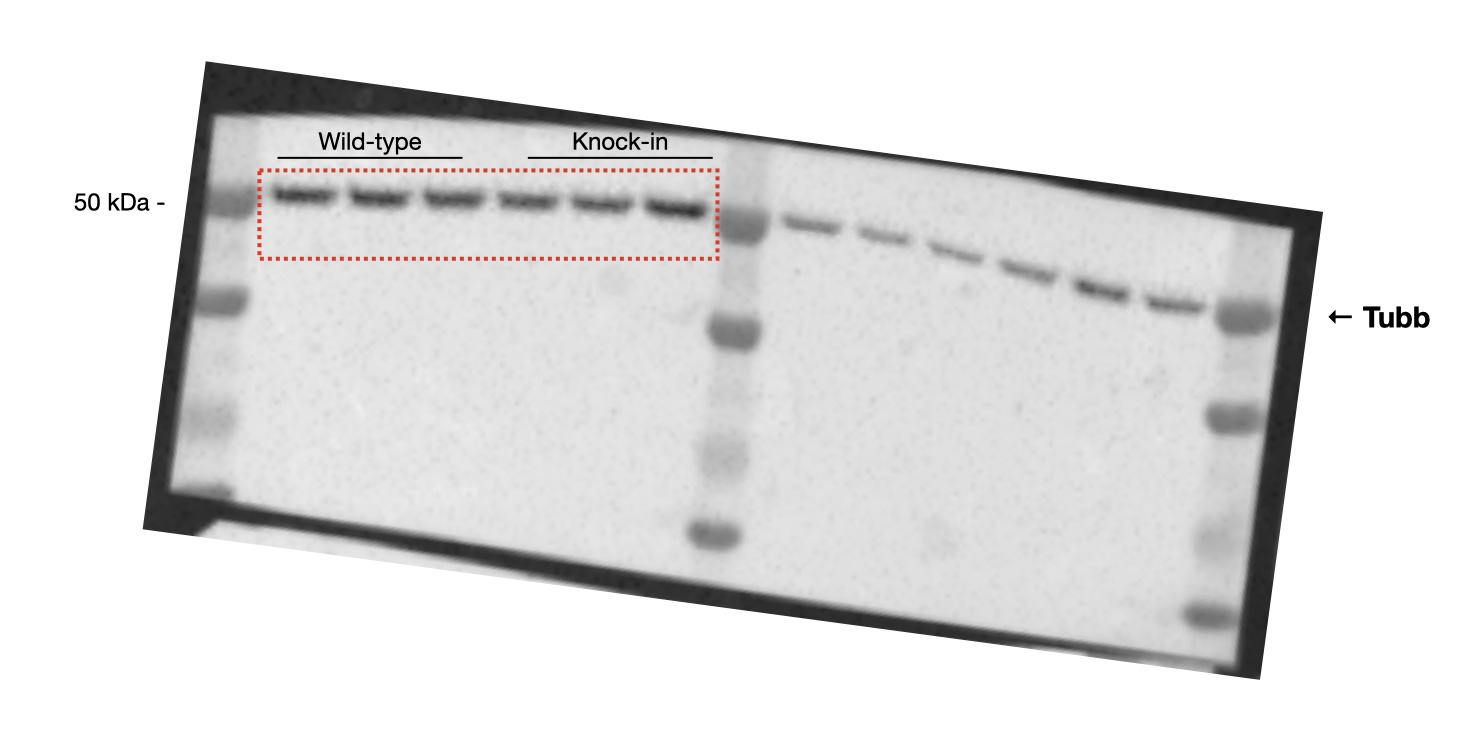

Supplement: Figure 1—figure supplement 3—source data 1. [file elife-82283-fig1-figsupp3-data1.zip › Fig1-SupFig3-Source data/Liver_Tubb_annotated.jpeg]

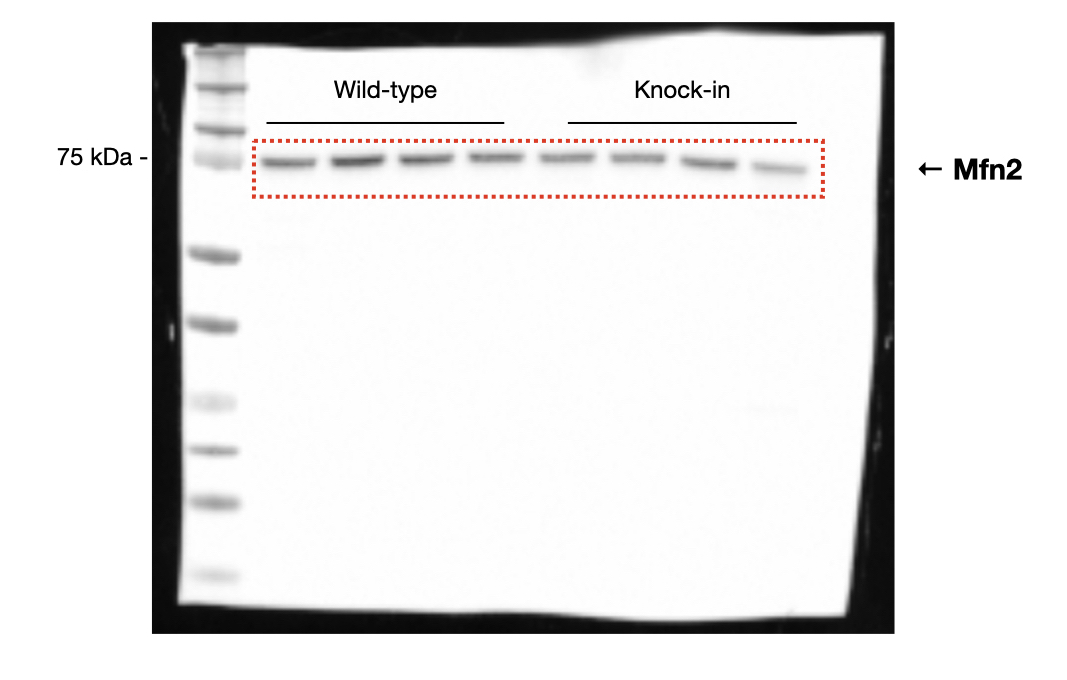

Supplement: Figure 1—figure supplement 3—source data 1. [file elife-82283-fig1-figsupp3-data1.zip › Fig1-SupFig3-Source data/BAT_Mfn2_annotated.jpeg]

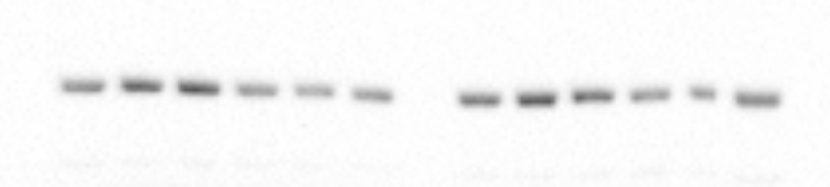

Supplement: Figure 1—figure supplement 3—source data 1. [file elife-82283-fig1-figsupp3-data1.zip › Fig1-SupFig3-Source data/SkelMusc_Mfn2_raw.tif]

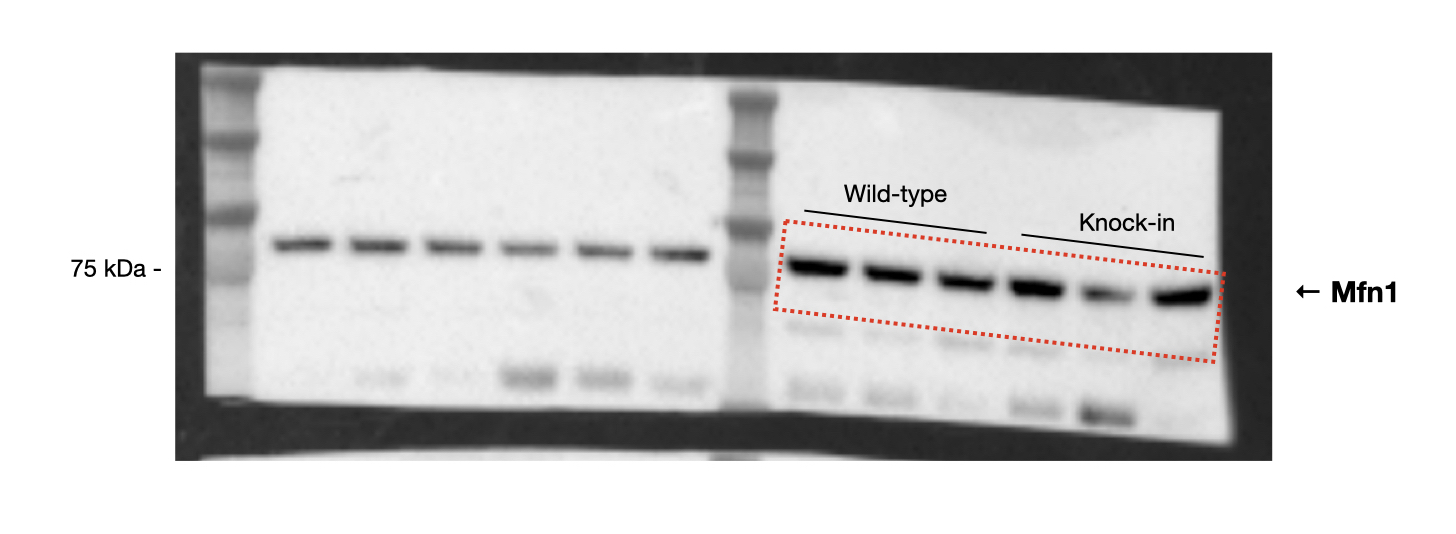

Supplement: Figure 1—figure supplement 3—source data 1. [file elife-82283-fig1-figsupp3-data1.zip › Fig1-SupFig3-Source data/Heart_Mfn1_annotated.jpeg]

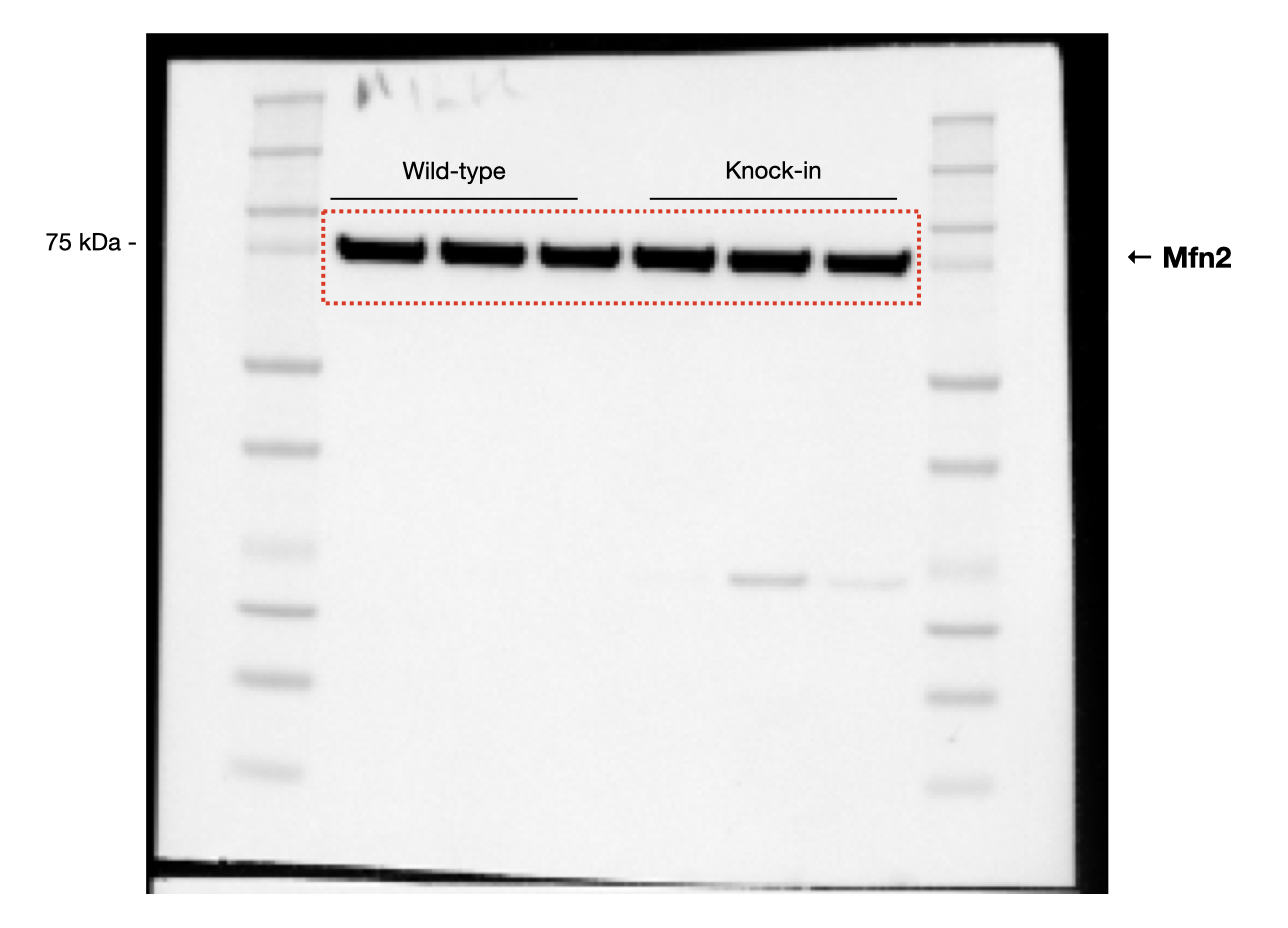

Supplement: Figure 1—figure supplement 3—source data 1. [file elife-82283-fig1-figsupp3-data1.zip › Fig1-SupFig3-Source data/IngWAT_Mfn2_annotated.jpeg]

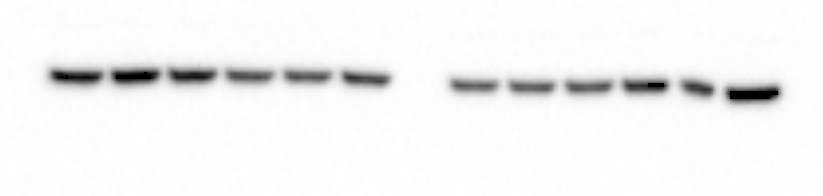

Supplement: Figure 1—figure supplement 3—source data 1. [file elife-82283-fig1-figsupp3-data1.zip › Fig1-SupFig3-Source data/SkelMusc_Tubb_raw.tif]

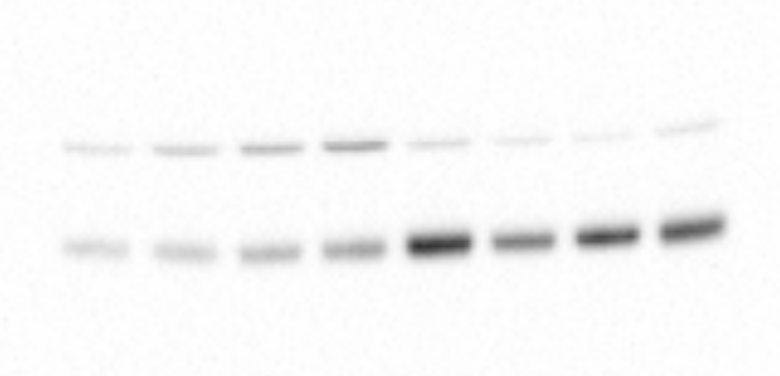

Supplement: Figure 1—figure supplement 3—source data 1. [file elife-82283-fig1-figsupp3-data1.zip › Fig1-SupFig3-Source data/BAT_Mfn1_raw.tif]

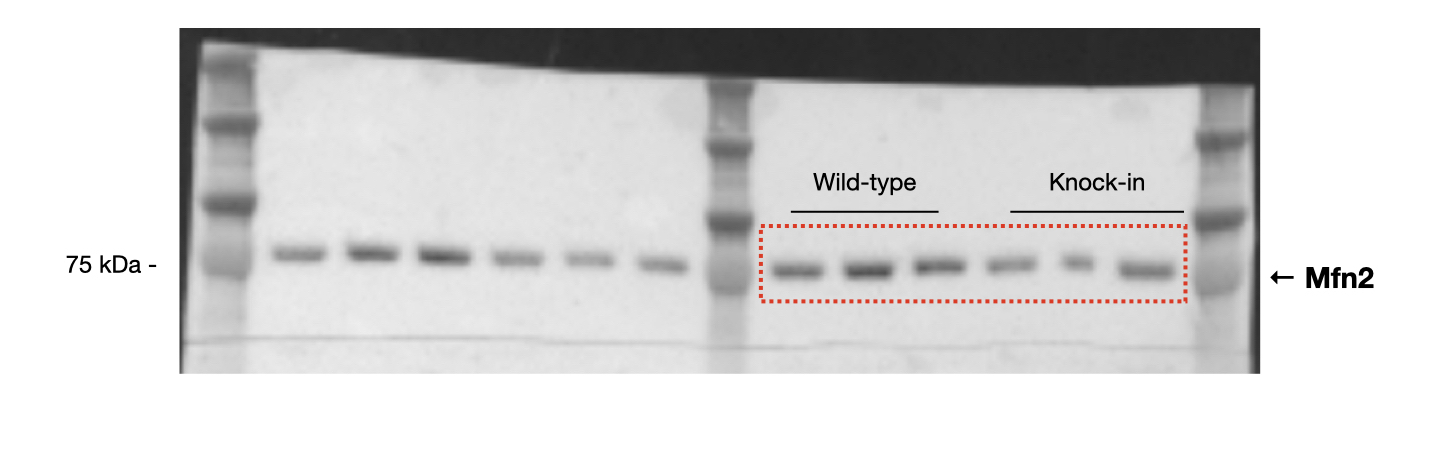

Supplement: Figure 1—figure supplement 3—source data 1. [file elife-82283-fig1-figsupp3-data1.zip › Fig1-SupFig3-Source data/SkelMusc_Mfn2_annotated.jpeg]

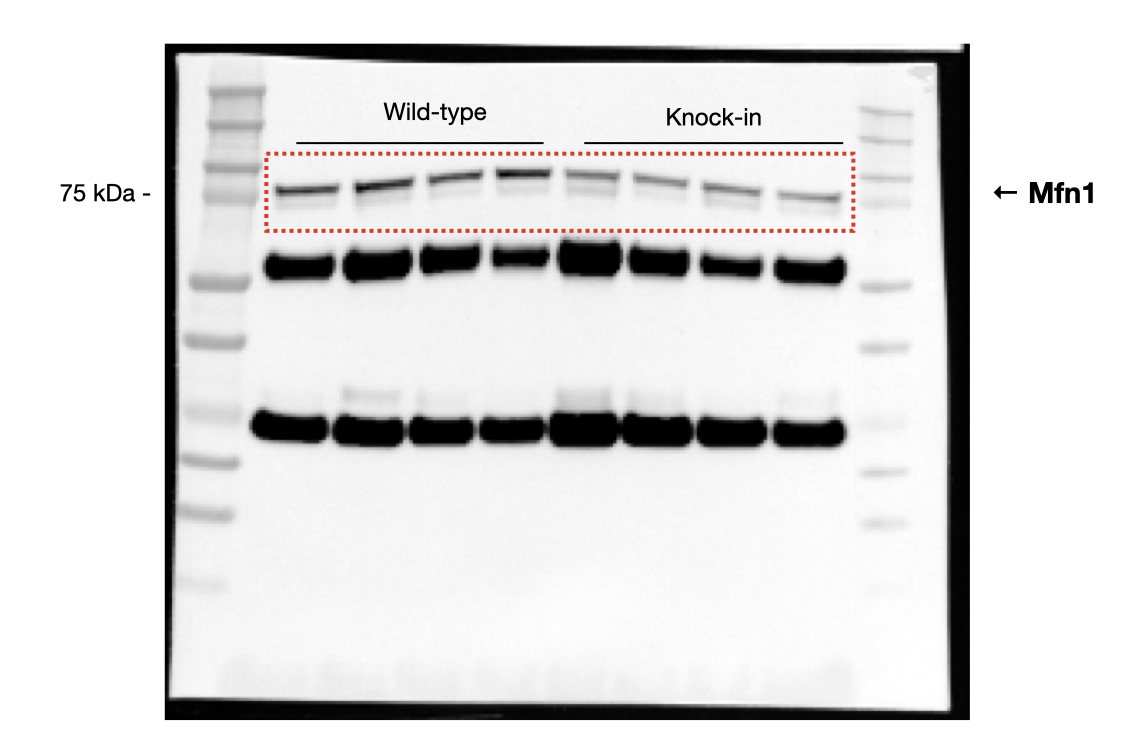

Supplement: Figure 1—figure supplement 3—source data 1. [file elife-82283-fig1-figsupp3-data1.zip › Fig1-SupFig3-Source data/EpiWAT_Mfn1_annotated.jpeg]

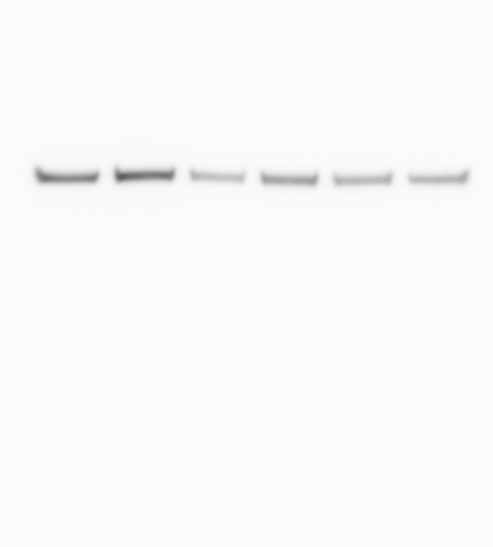

Supplement: Figure 1—figure supplement 3—source data 1. [file elife-82283-fig1-figsupp3-data1.zip › Fig1-SupFig3-Source data/IngWAT_Mfn2_raw.tif]

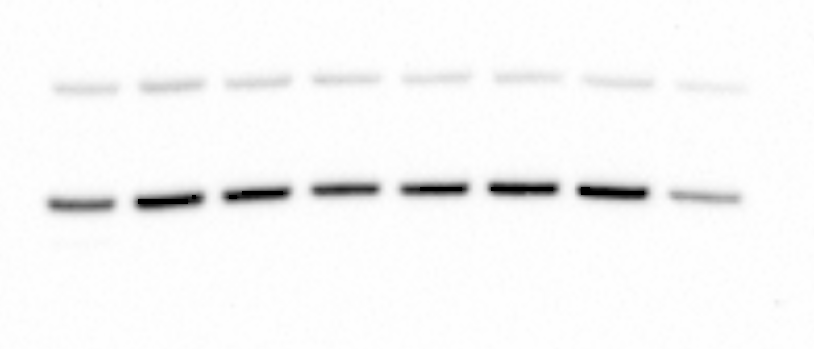

Supplement: Figure 1—figure supplement 3—source data 1. [file elife-82283-fig1-figsupp3-data1.zip › Fig1-SupFig3-Source data/BAT_Tubb_raw.tif]

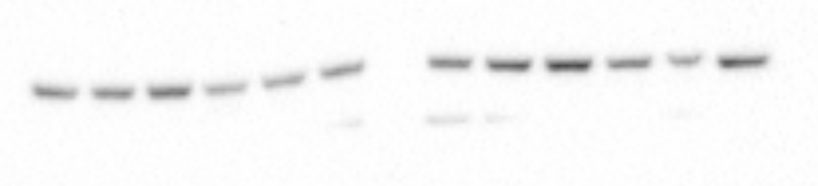

Supplement: Figure 1—figure supplement 3—source data 1. [file elife-82283-fig1-figsupp3-data1.zip › Fig1-SupFig3-Source data/SkelMusc_Mfn1_raw.tif]

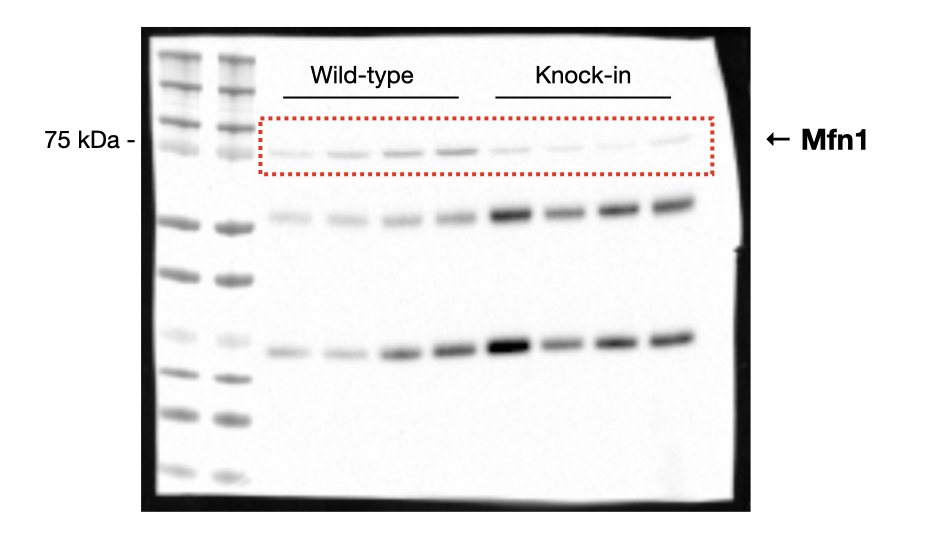

Supplement: Figure 1—figure supplement 3—source data 1. [file elife-82283-fig1-figsupp3-data1.zip › Fig1-SupFig3-Source data/BAT_Mfn1_annotated.jpeg]

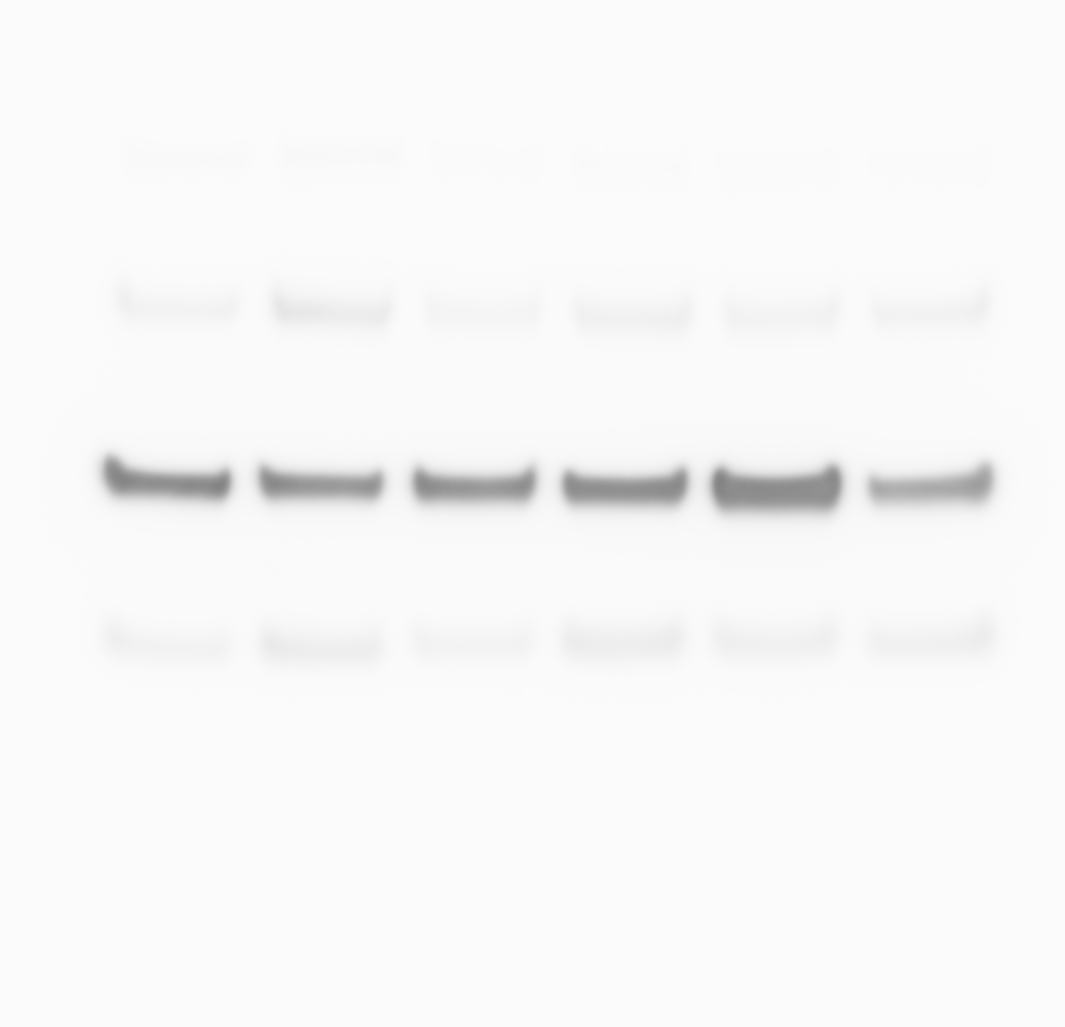

Supplement: Figure 1—figure supplement 3—source data 1. [file elife-82283-fig1-figsupp3-data1.zip › Fig1-SupFig3-Source data/IngWAT_Gapdh_raw.tif]

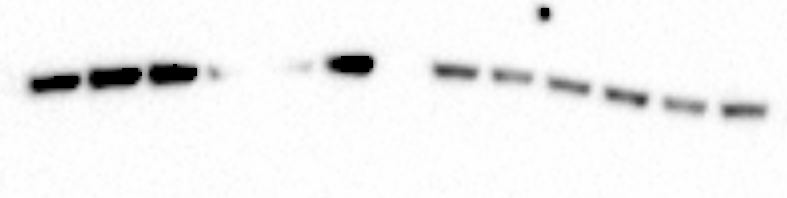

Supplement: Figure 1—figure supplement 3—source data 1. [file elife-82283-fig1-figsupp3-data1.zip › Fig1-SupFig3-Source data/Heart_Mfn2_raw.tif]

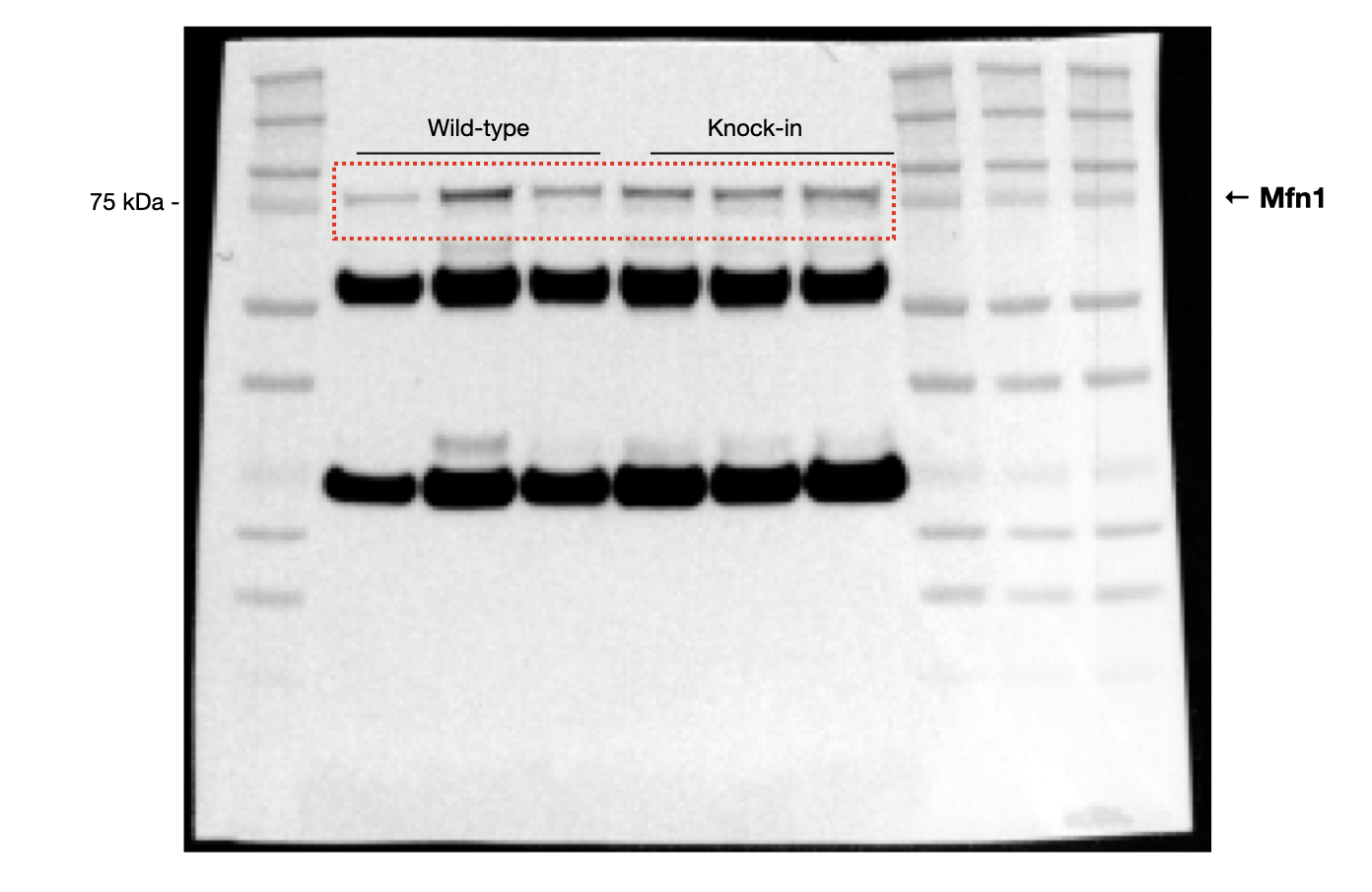

Supplement: Figure 1—figure supplement 3—source data 1. [file elife-82283-fig1-figsupp3-data1.zip › Fig1-SupFig3-Source data/IngWAT_Mfn1_annotated.jpeg]

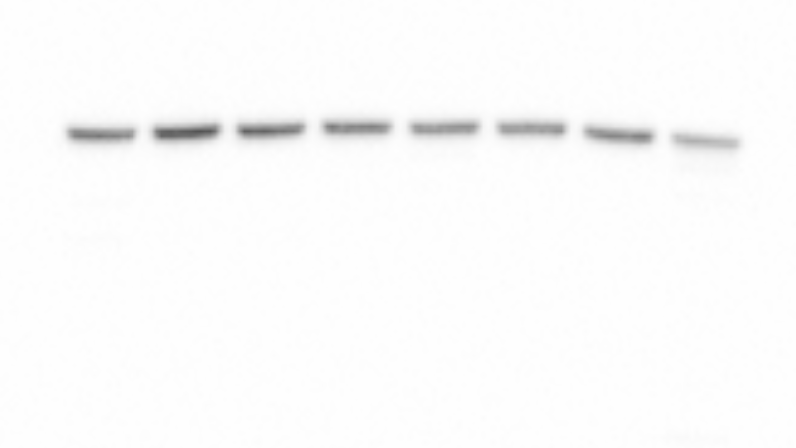

Supplement: Figure 1—figure supplement 3—source data 1. [file elife-82283-fig1-figsupp3-data1.zip › Fig1-SupFig3-Source data/BAT_Mfn2_raw.tif]

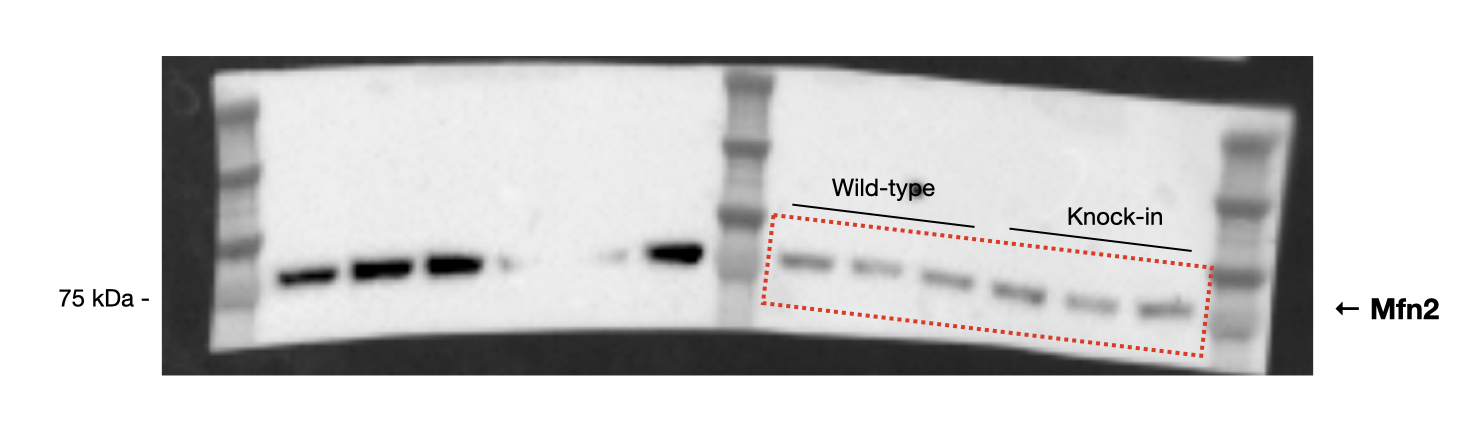

Supplement: Figure 1—figure supplement 3—source data 1. [file elife-82283-fig1-figsupp3-data1.zip › Fig1-SupFig3-Source data/Heart_Mfn2_annotated.jpeg]

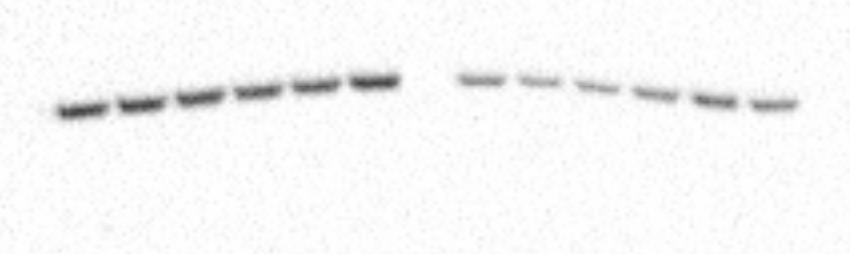

Supplement: Figure 1—figure supplement 3—source data 1. [file elife-82283-fig1-figsupp3-data1.zip › Fig1-SupFig3-Source data/Heart_Tubb_raw.tif]

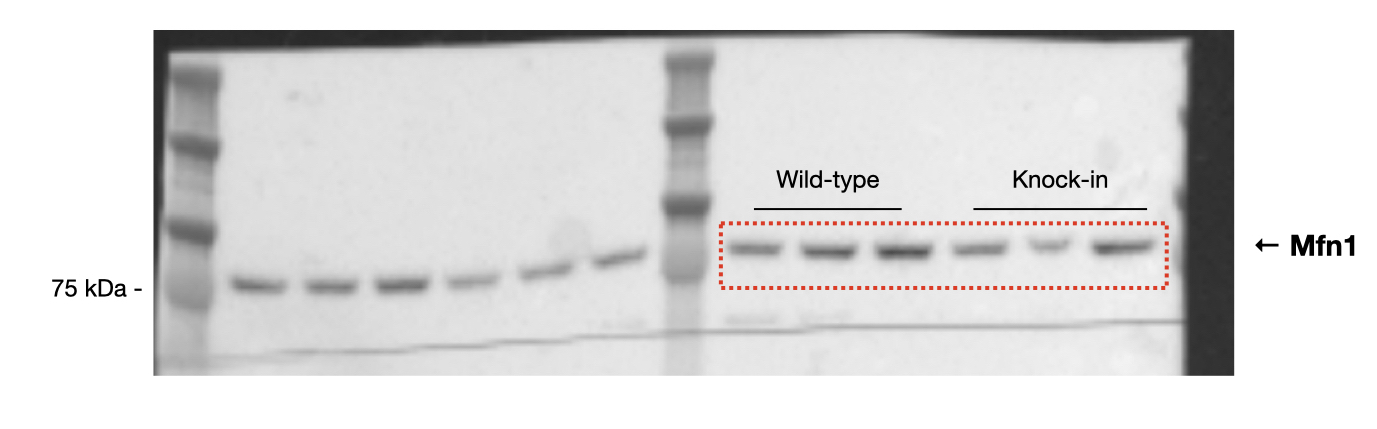

Supplement: Figure 1—figure supplement 3—source data 1. [file elife-82283-fig1-figsupp3-data1.zip › Fig1-SupFig3-Source data/SkelMusc_Mfn1_annotated.jpeg]

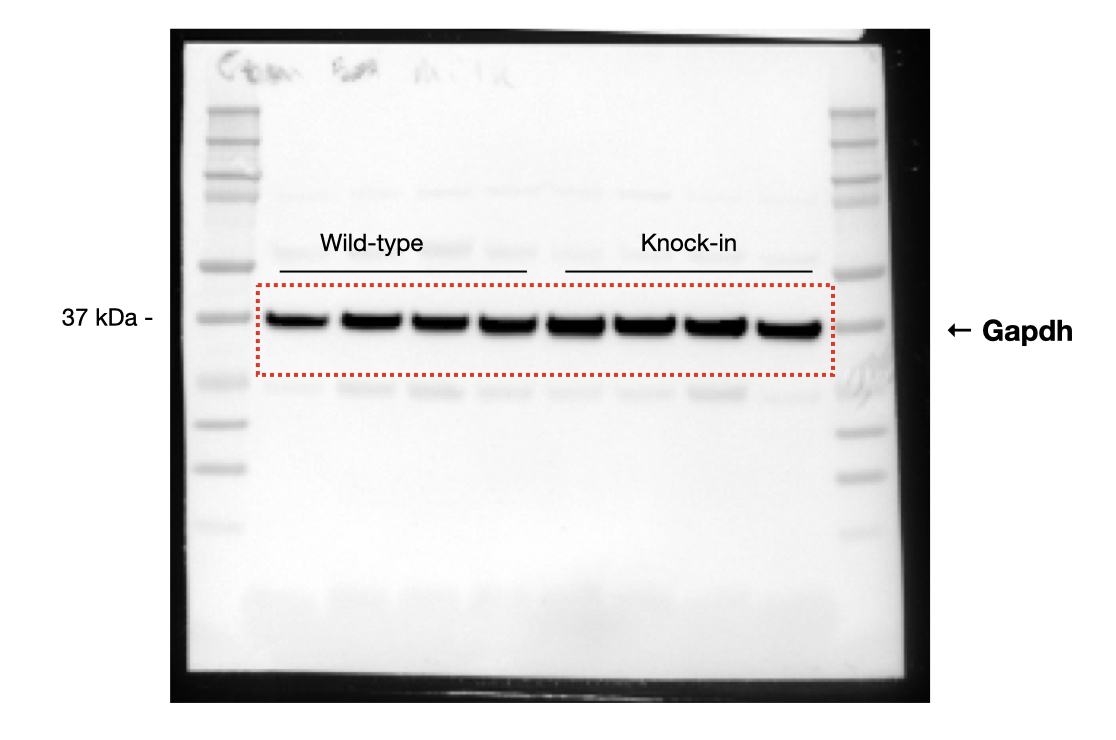

Supplement: Figure 1—figure supplement 3—source data 1. [file elife-82283-fig1-figsupp3-data1.zip › Fig1-SupFig3-Source data/EpiWAT_Gapdh_annotated.jpeg]

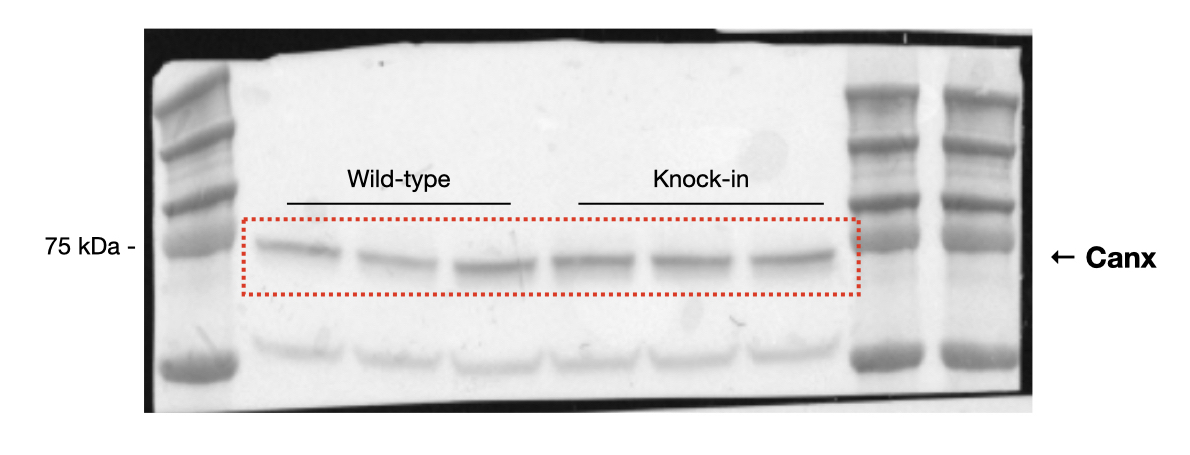

Supplement: Figure 2—figure supplement 3—source data 1. [file elife-82283-fig2-figsupp3-data1.zip › Fig2-SupFig3-Source data/Liver_Canx_annotated.jpeg]

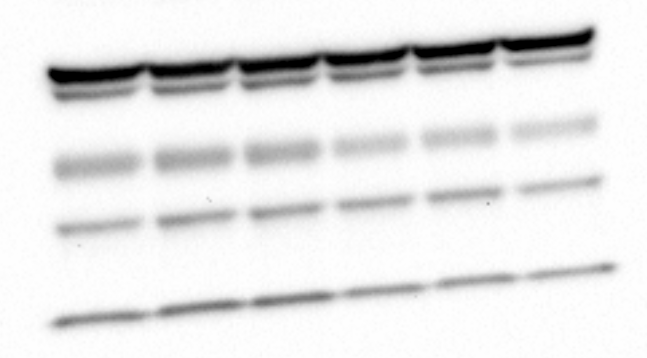

Supplement: Figure 2—figure supplement 3—source data 1. [file elife-82283-fig2-figsupp3-data1.zip › Fig2-SupFig3-Source data/BAT_Oxphos_raw.tif]

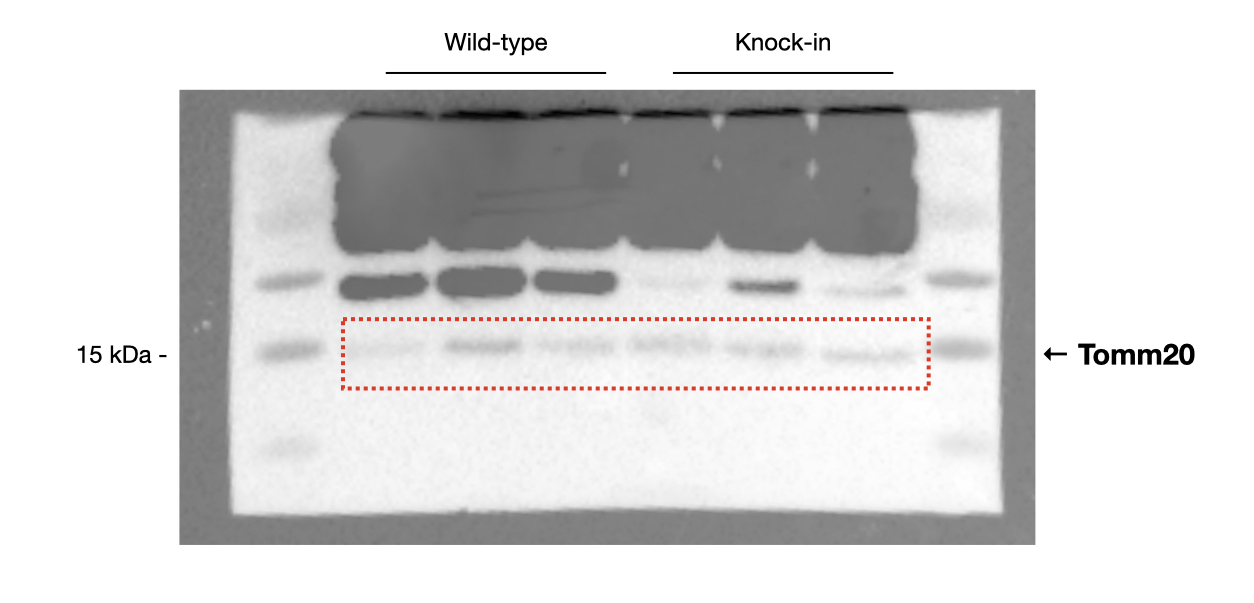

Supplement: Figure 2—figure supplement 3—source data 1. [file elife-82283-fig2-figsupp3-data1.zip › Fig2-SupFig3-Source data/IngWAT_Tomm20_annotated.jpeg]

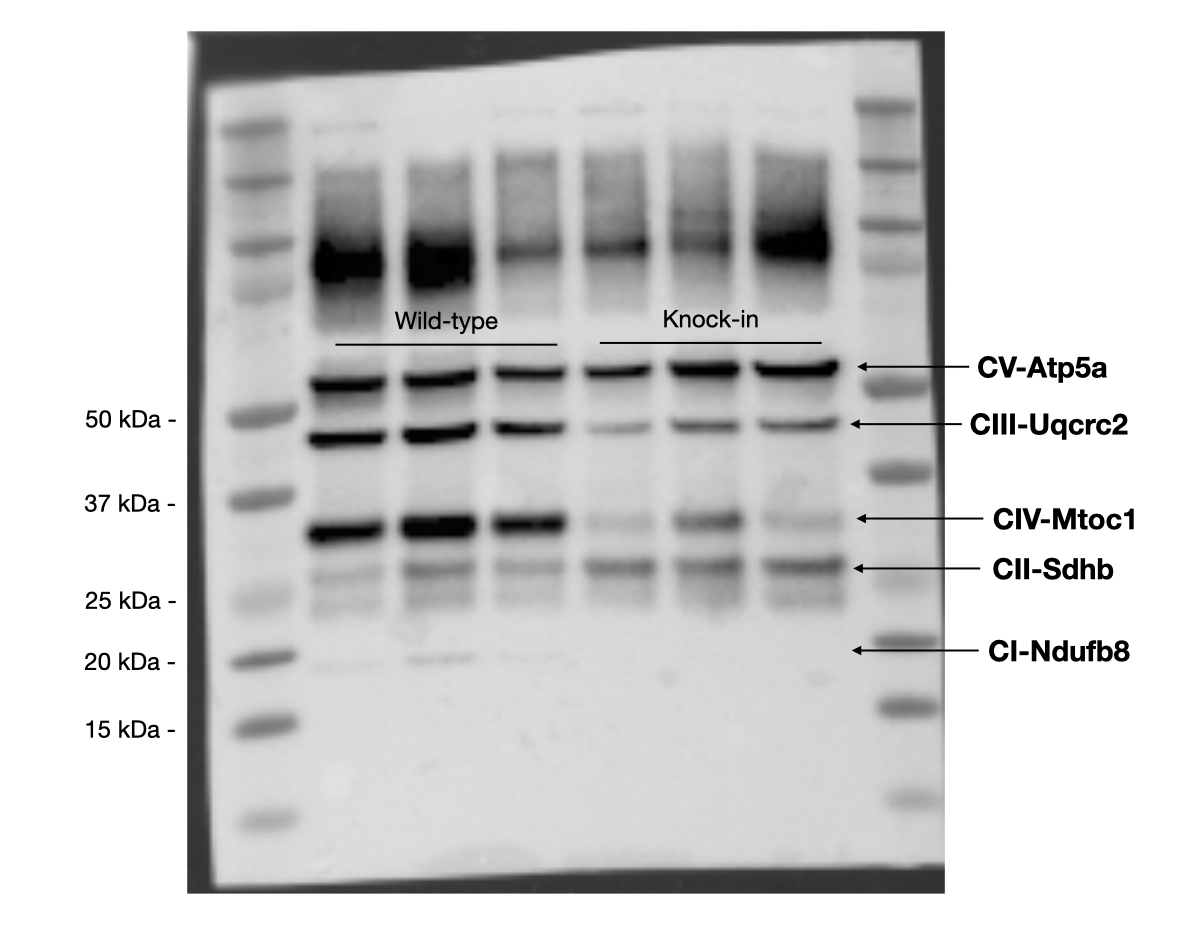

Supplement: Figure 2—figure supplement 3—source data 1. [file elife-82283-fig2-figsupp3-data1.zip › Fig2-SupFig3-Source data/IngWAT_Oxphos_annotated.jpeg]

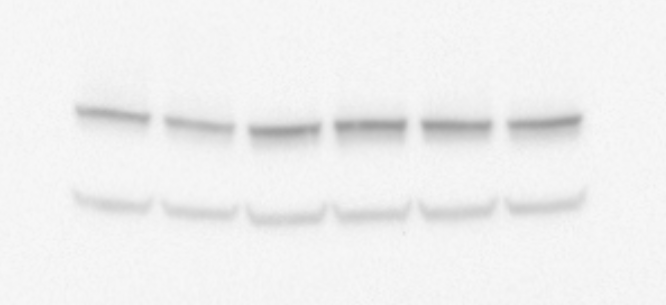

Supplement: Figure 2—figure supplement 3—source data 1. [file elife-82283-fig2-figsupp3-data1.zip › Fig2-SupFig3-Source data/Liver_Canx_raw.tif]

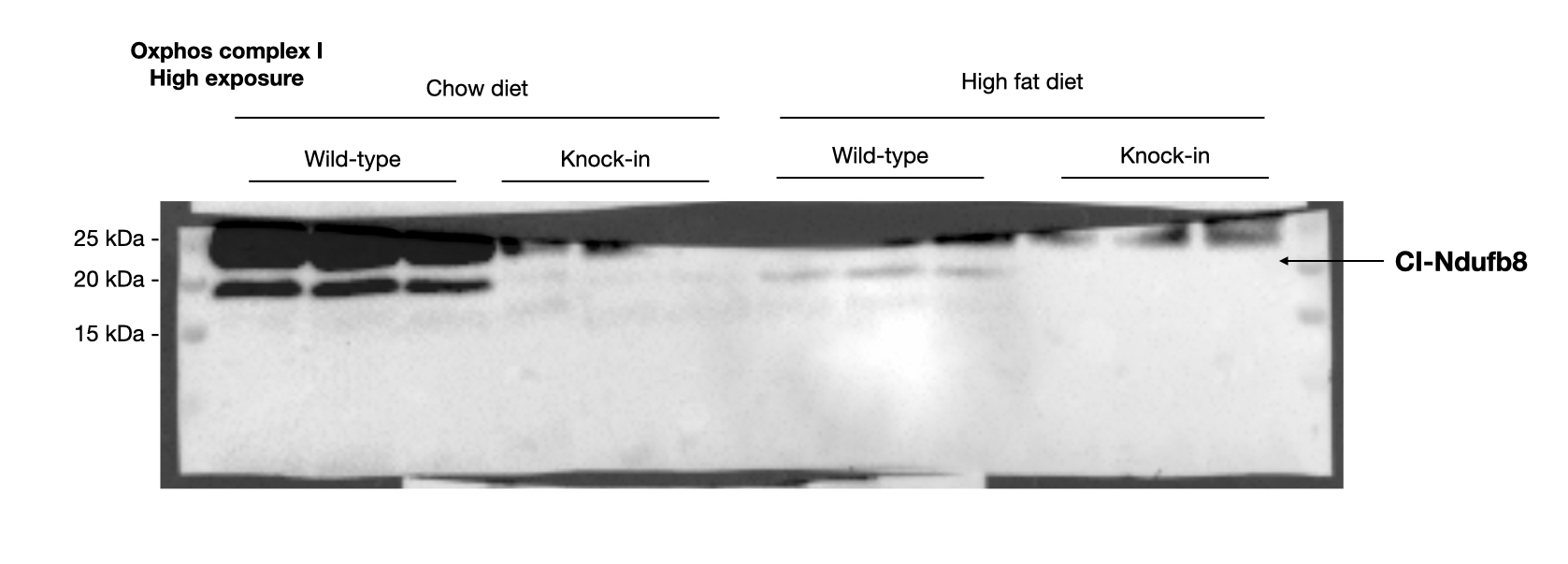

Supplement: Figure 2—figure supplement 3—source data 1. [file elife-82283-fig2-figsupp3-data1.zip › Fig2-SupFig3-Source data/EpiWAT_Oxphos_high_annotated.jpeg]

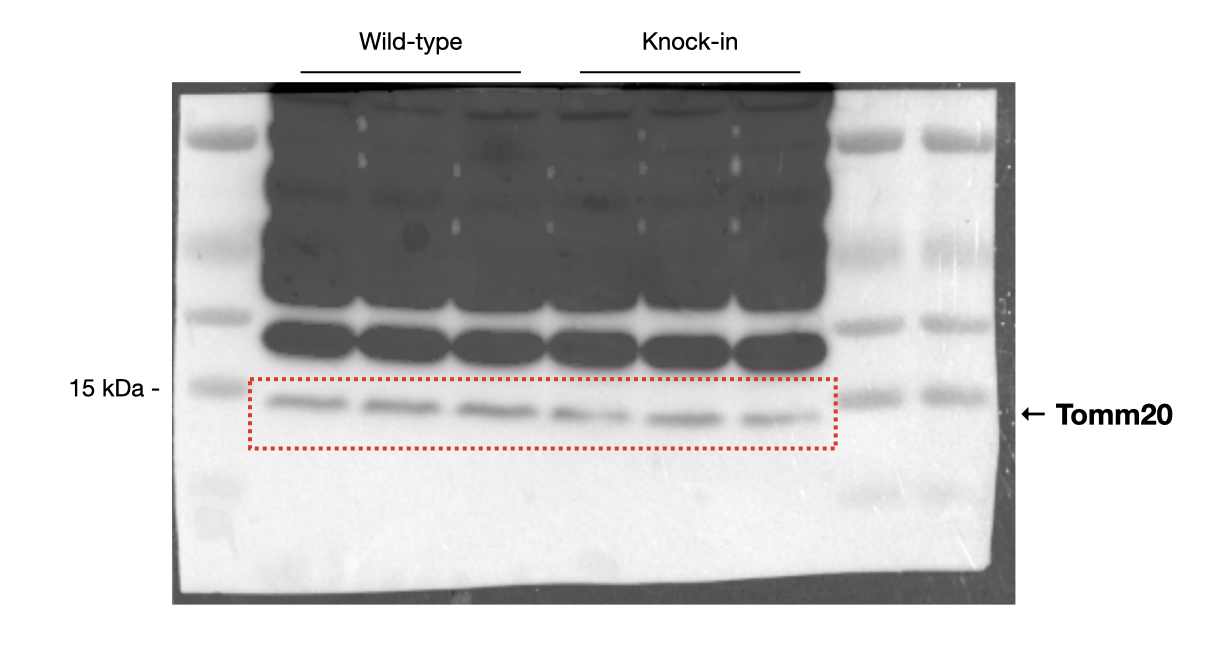

Supplement: Figure 2—figure supplement 3—source data 1. [file elife-82283-fig2-figsupp3-data1.zip › Fig2-SupFig3-Source data/Liver_Tomm20_annotated.jpeg]

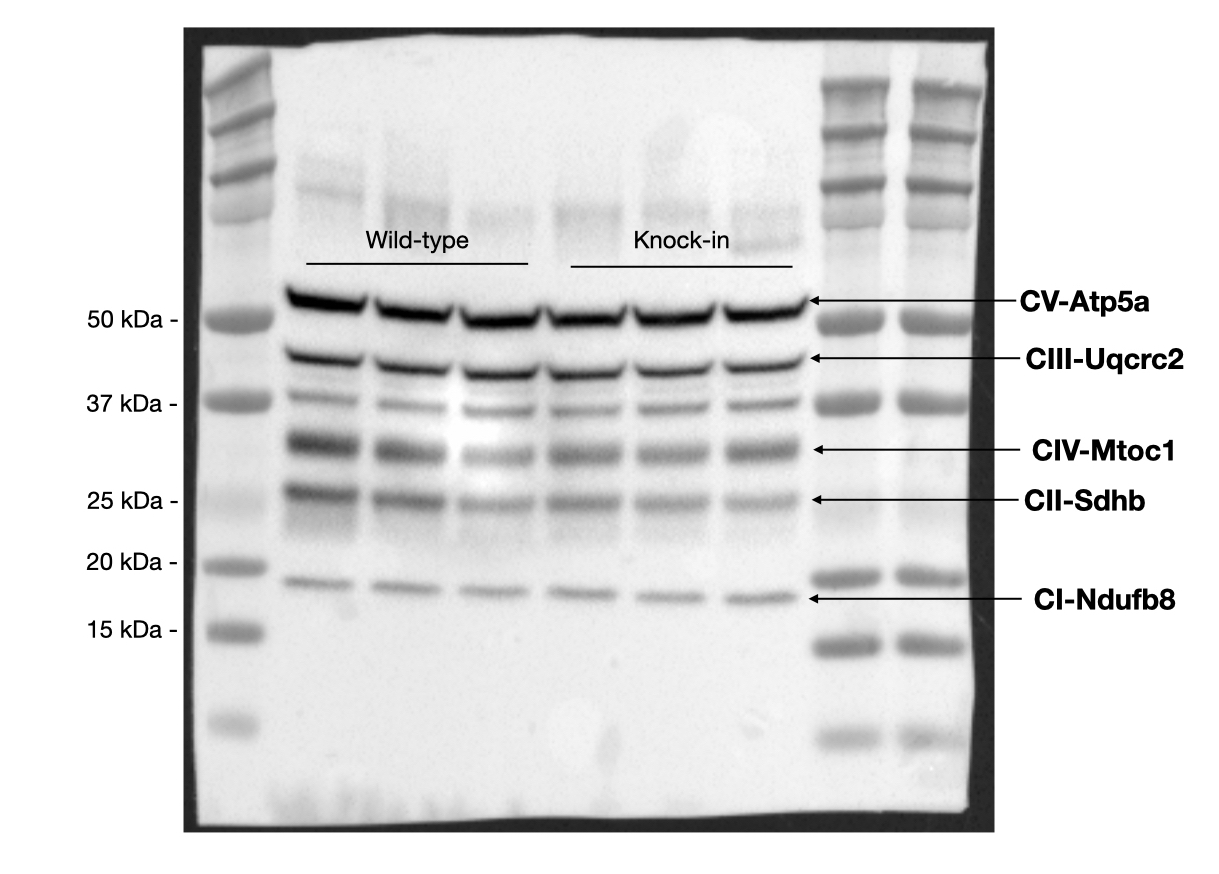

Supplement: Figure 2—figure supplement 3—source data 1. [file elife-82283-fig2-figsupp3-data1.zip › Fig2-SupFig3-Source data/Liver_Oxphos_annotated.jpeg]

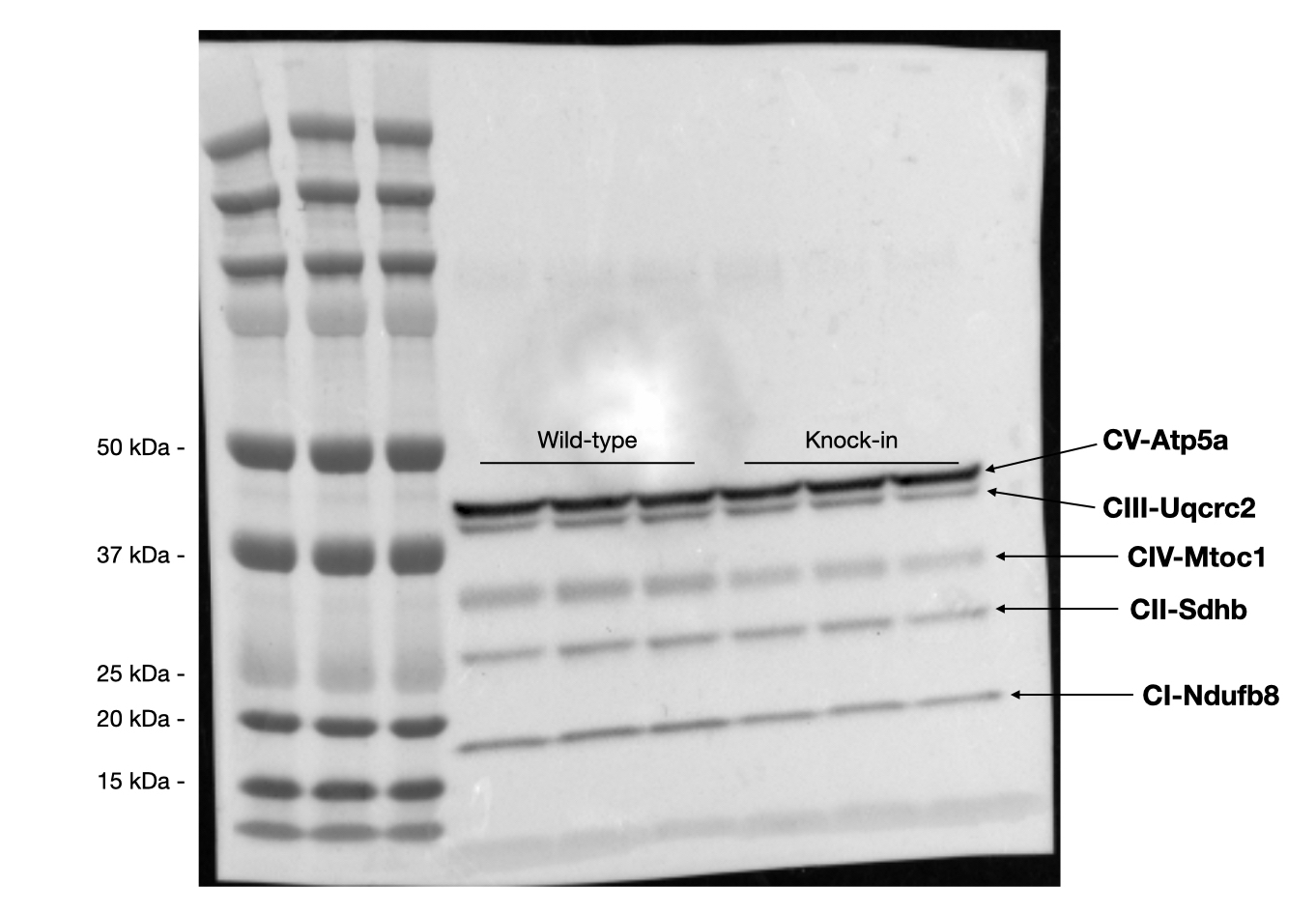

Supplement: Figure 2—figure supplement 3—source data 1. [file elife-82283-fig2-figsupp3-data1.zip › Fig2-SupFig3-Source data/BAT_Oxphos_annotated.jpeg]

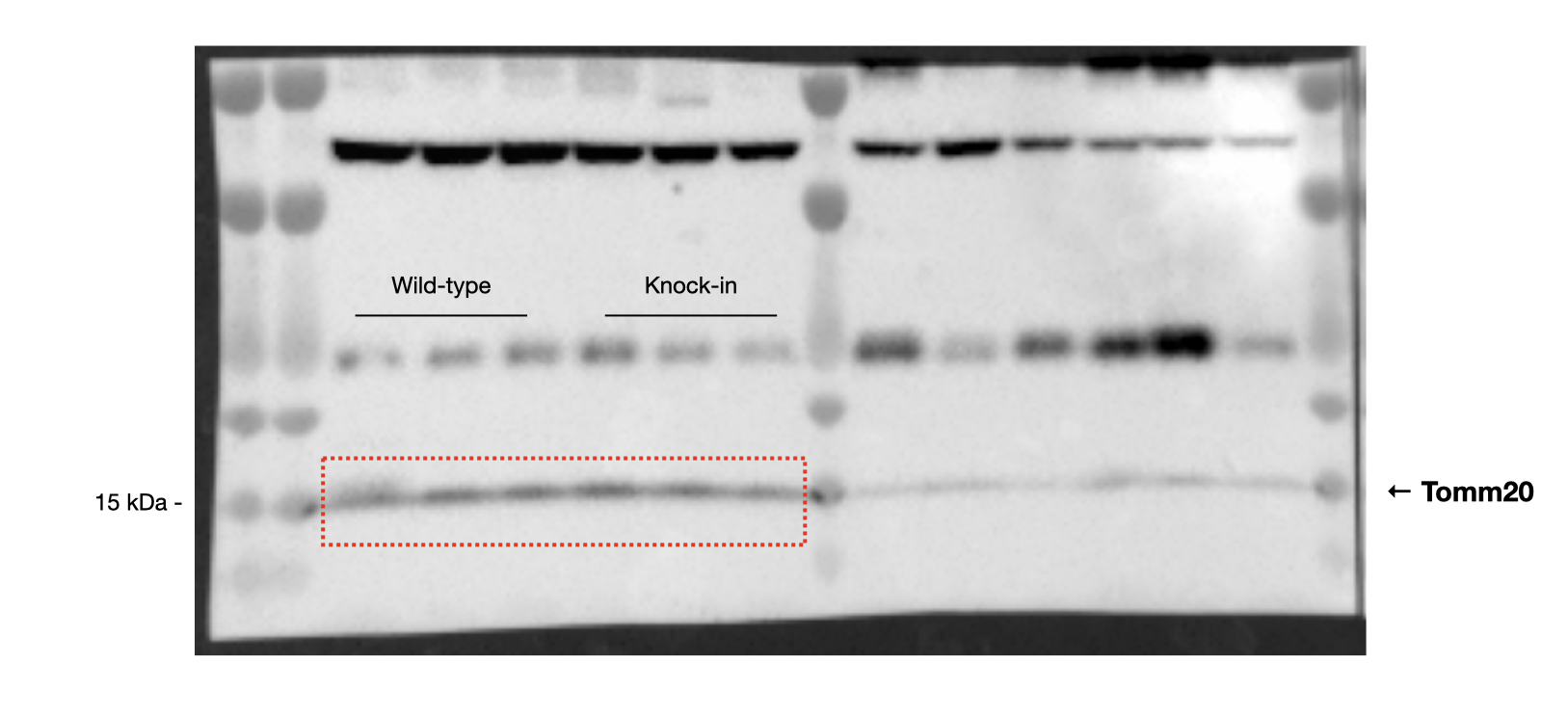

Supplement: Figure 2—figure supplement 3—source data 1. [file elife-82283-fig2-figsupp3-data1.zip › Fig2-SupFig3-Source data/BAT_Tomm20_annotated.jpeg]

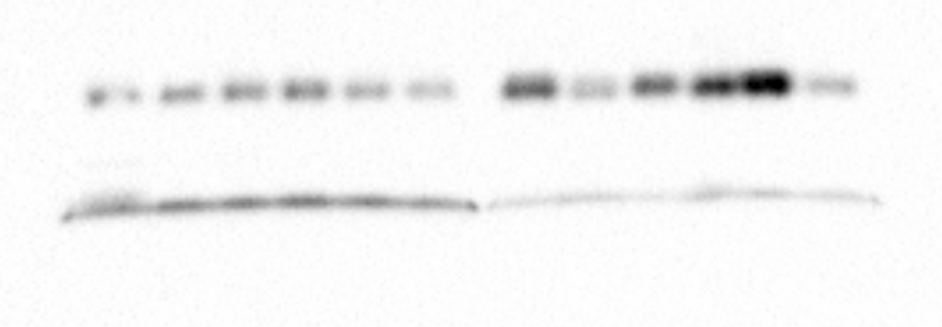

Supplement: Figure 2—figure supplement 3—source data 1. [file elife-82283-fig2-figsupp3-data1.zip › Fig2-SupFig3-Source data/BAT_Tomm20_raw.tif]

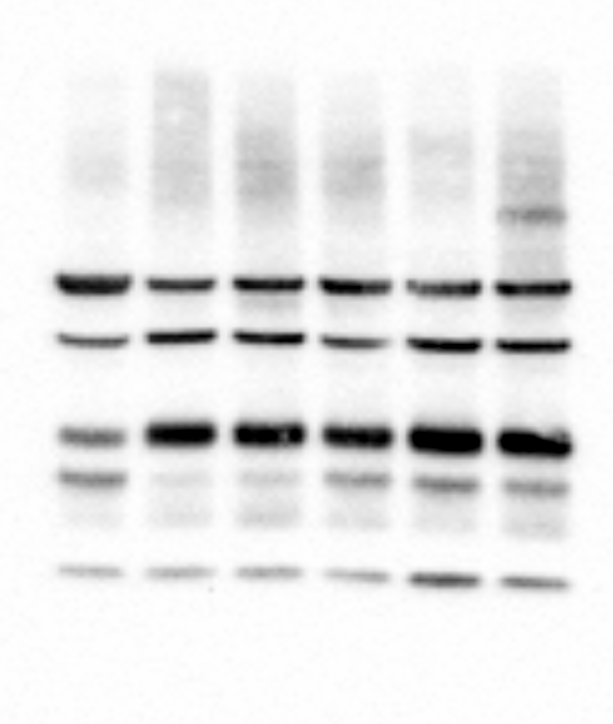

Supplement: Figure 2—figure supplement 3—source data 1. [file elife-82283-fig2-figsupp3-data1.zip › Fig2-SupFig3-Source data/Heart_Oxphos_raw.tif]

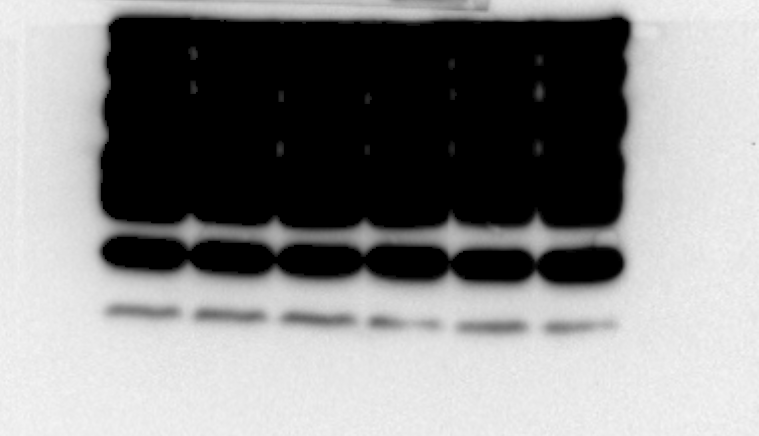

Supplement: Figure 2—figure supplement 3—source data 1. [file elife-82283-fig2-figsupp3-data1.zip › Fig2-SupFig3-Source data/Liver_Tomm20_raw.tif]

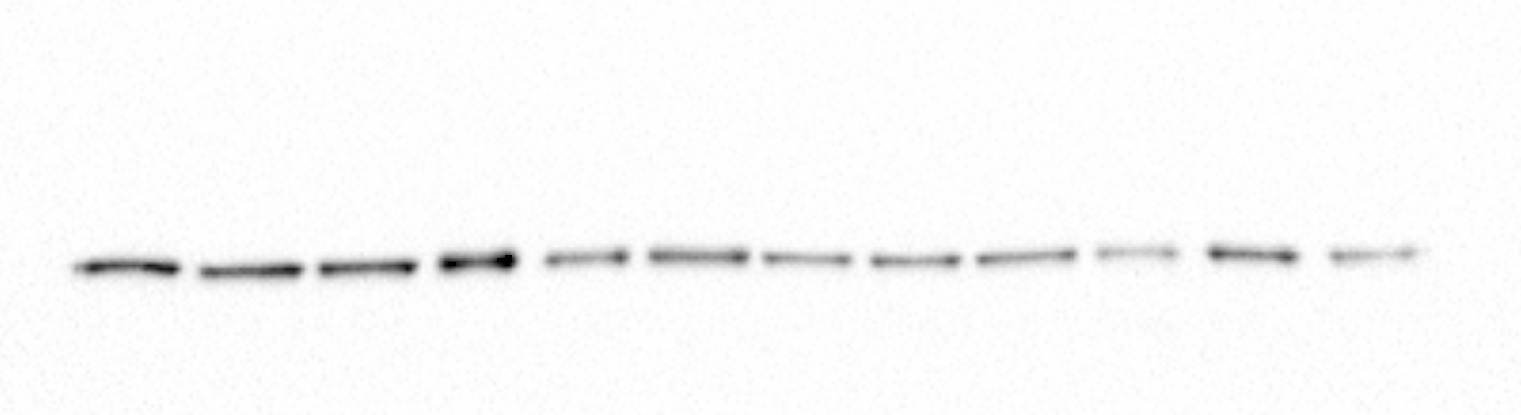

Supplement: Figure 2—figure supplement 3—source data 1. [file elife-82283-fig2-figsupp3-data1.zip › Fig2-SupFig3-Source data/EpiWAT_Cs_raw.tif]

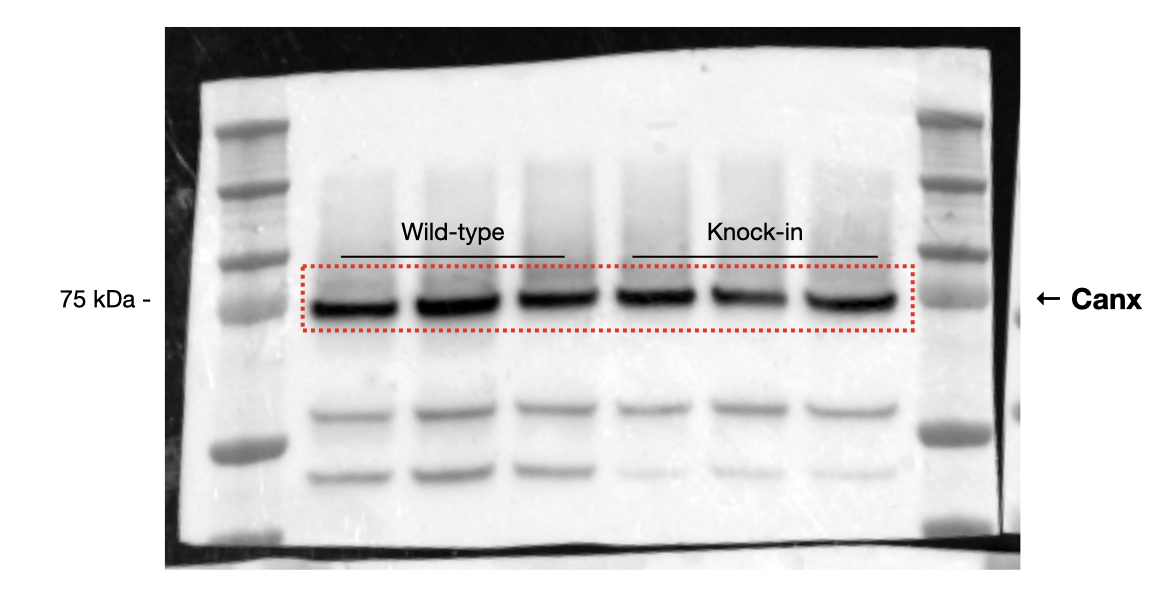

Supplement: Figure 2—figure supplement 3—source data 1. [file elife-82283-fig2-figsupp3-data1.zip › Fig2-SupFig3-Source data/IngWAT_Canx_annotated.jpeg]

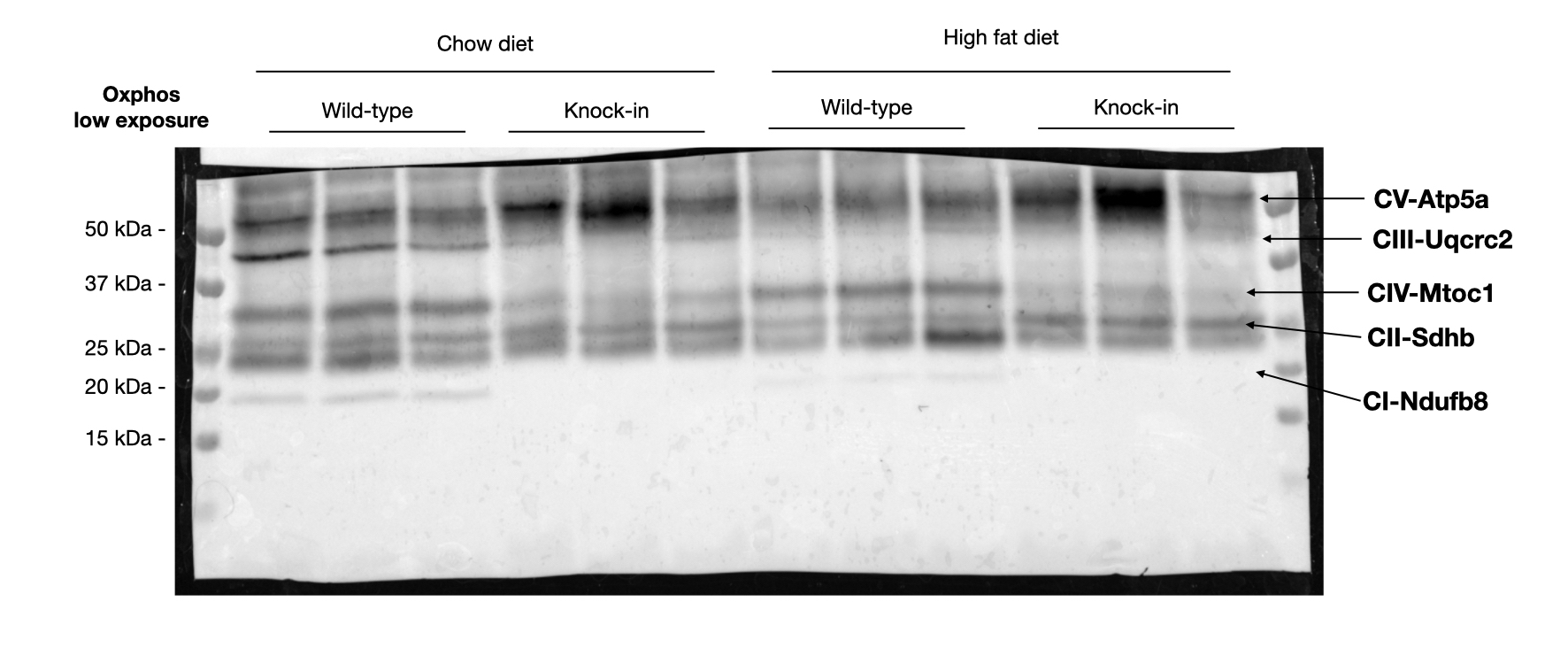

Supplement: Figure 2—figure supplement 3—source data 1. [file elife-82283-fig2-figsupp3-data1.zip › Fig2-SupFig3-Source data/EpiWAT_Oxphos_low_annotated.jpeg]

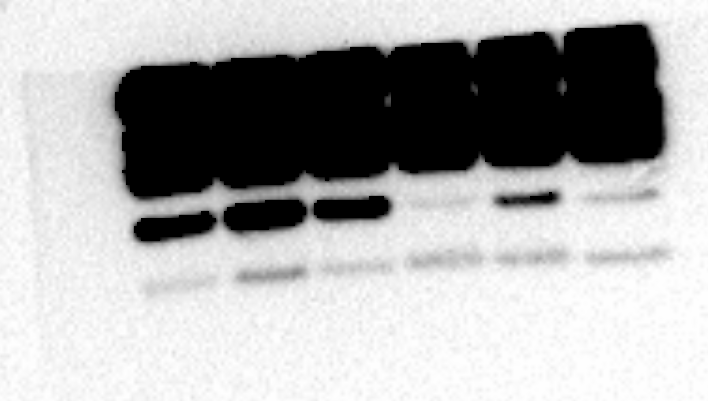

Supplement: Figure 2—figure supplement 3—source data 1. [file elife-82283-fig2-figsupp3-data1.zip › Fig2-SupFig3-Source data/IngWAT_Tomm20_raw.tif]

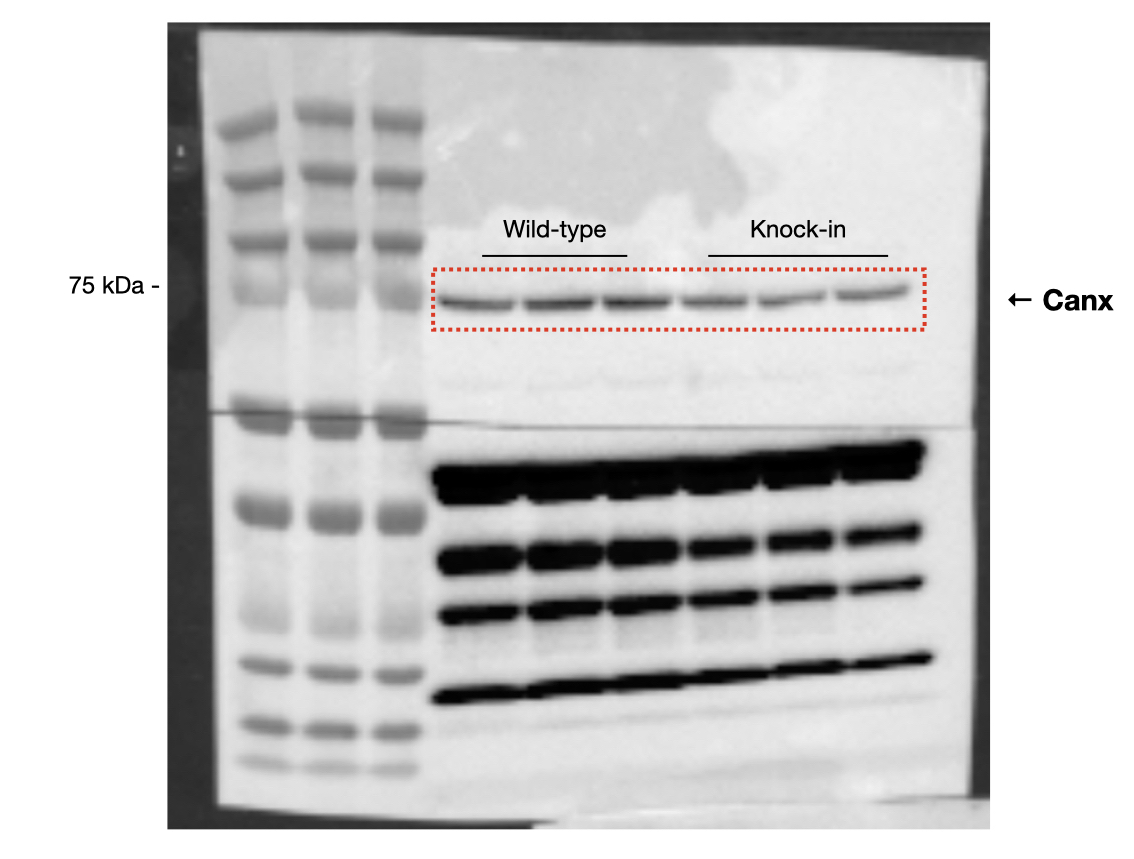

Supplement: Figure 2—figure supplement 3—source data 1. [file elife-82283-fig2-figsupp3-data1.zip › Fig2-SupFig3-Source data/BAT_Canx_annotated.jpeg]

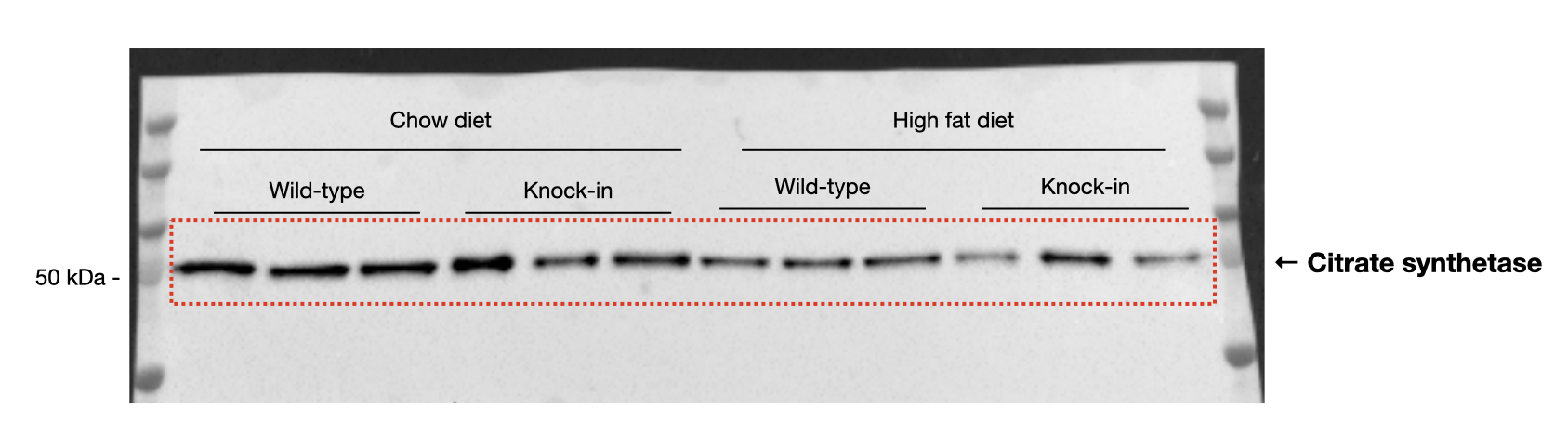

Supplement: Figure 2—figure supplement 3—source data 1. [file elife-82283-fig2-figsupp3-data1.zip › Fig2-SupFig3-Source data/EpiWAT_Cs_annotated.jpeg]
