# Supplementary figures and images for: A mouse model of human mitofusin-2-related lipodystrophy exhibits adipose-specific mitochondrial stress and reduced leptin secretion (part 2 of 2)
Source: eLife. 2023 Feb 1;12:e82283. doi: 10.7554/eLife.82283 (PMC9937658; doi:10.7554/eLife.82283)

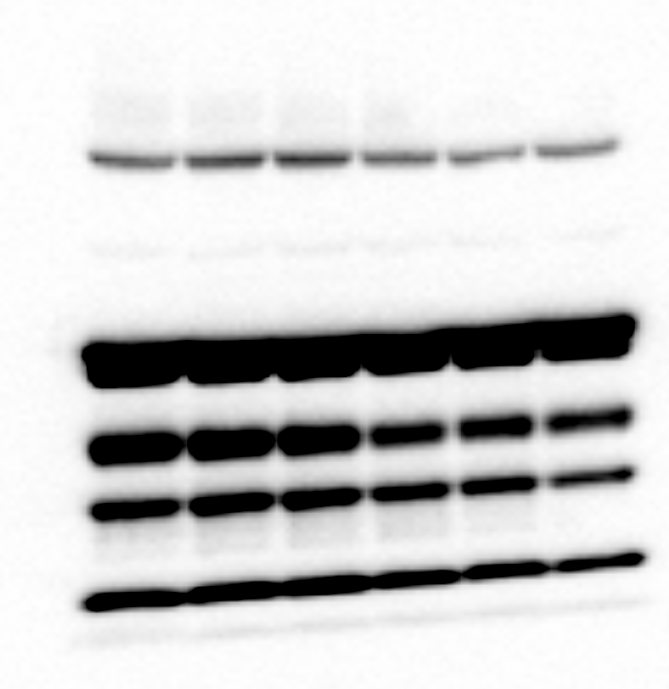

Supplement: Figure 2—figure supplement 3—source data 1. [file elife-82283-fig2-figsupp3-data1.zip › Fig2-SupFig3-Source data/BAT_Canx_raw.tif]

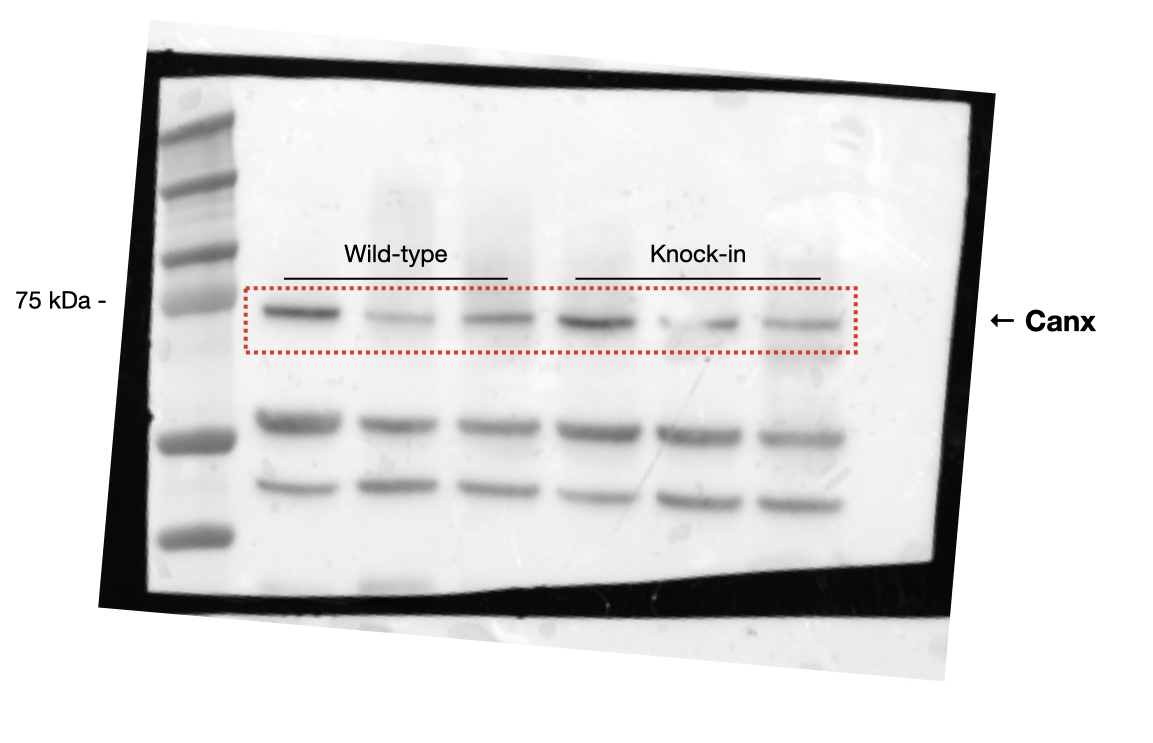

Supplement: Figure 2—figure supplement 3—source data 1. [file elife-82283-fig2-figsupp3-data1.zip › Fig2-SupFig3-Source data/Heart_Canx_annotated.jpeg]

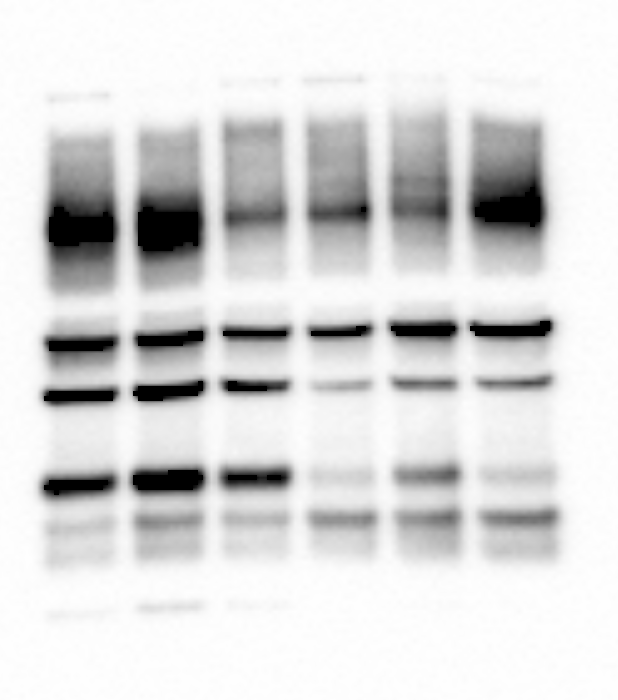

Supplement: Figure 2—figure supplement 3—source data 1. [file elife-82283-fig2-figsupp3-data1.zip › Fig2-SupFig3-Source data/IngWAT_Oxphos_raw.tif]

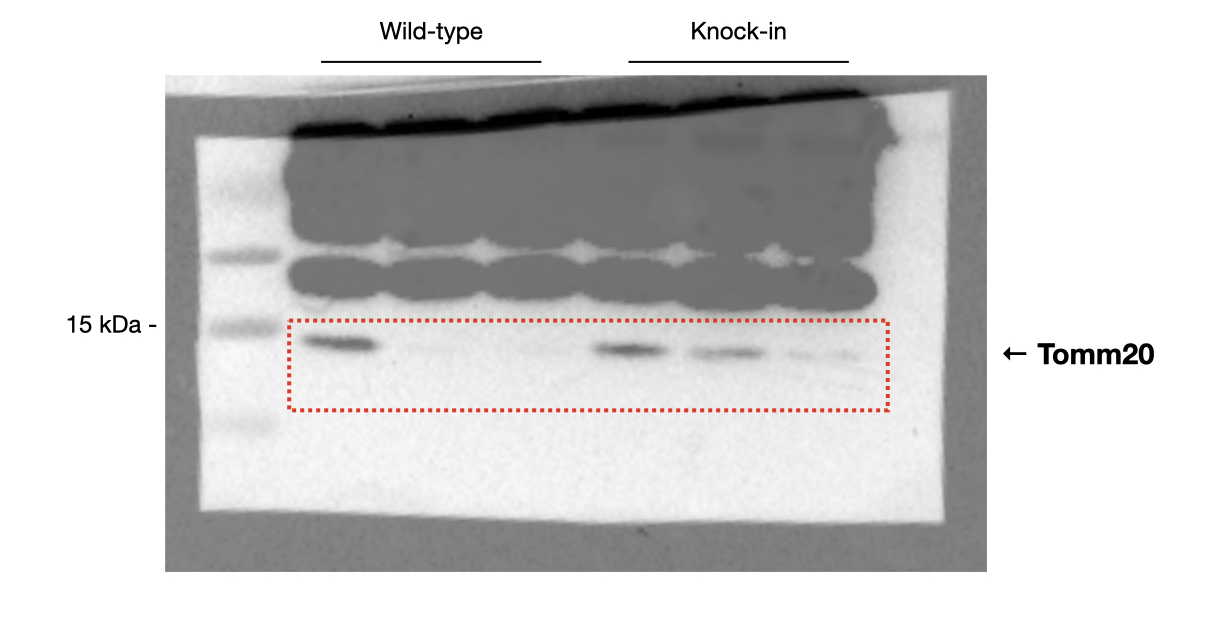

Supplement: Figure 2—figure supplement 3—source data 1. [file elife-82283-fig2-figsupp3-data1.zip › Fig2-SupFig3-Source data/Heart_Tomm20_annotated.jpeg]

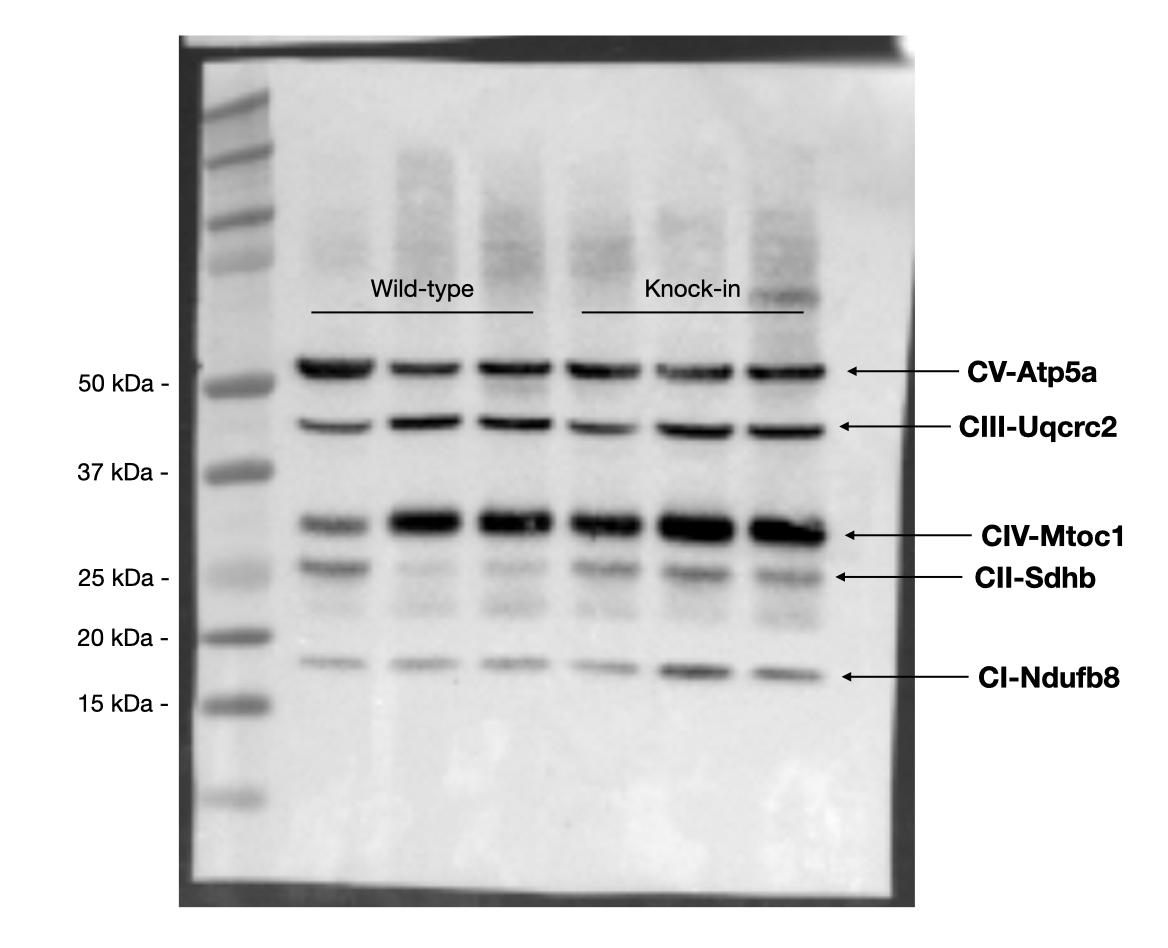

Supplement: Figure 2—figure supplement 3—source data 1. [file elife-82283-fig2-figsupp3-data1.zip › Fig2-SupFig3-Source data/Heart_Oxphos_annotated.jpeg]

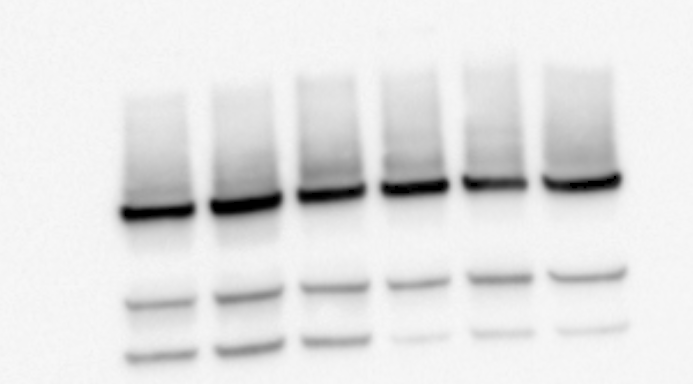

Supplement: Figure 2—figure supplement 3—source data 1. [file elife-82283-fig2-figsupp3-data1.zip › Fig2-SupFig3-Source data/IngWAT_Canx_raw.tif]

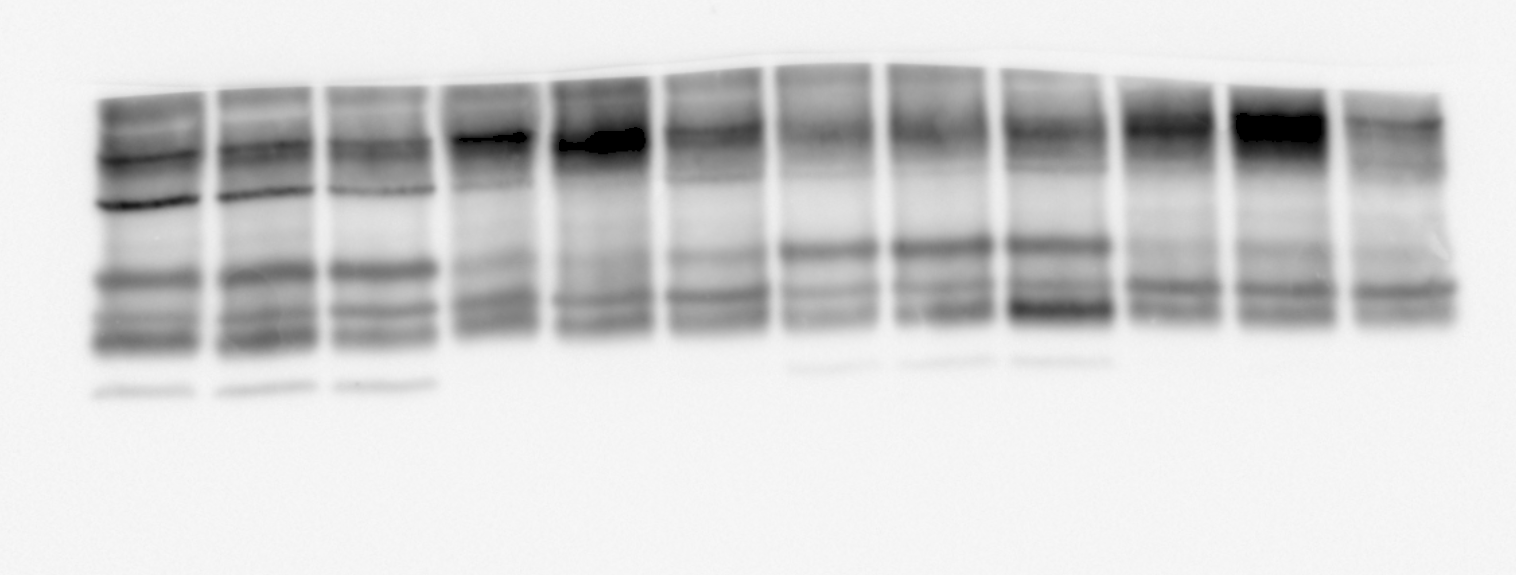

Supplement: Figure 2—figure supplement 3—source data 1. [file elife-82283-fig2-figsupp3-data1.zip › Fig2-SupFig3-Source data/EpiWAT_Oxphos_raw_low.tif]

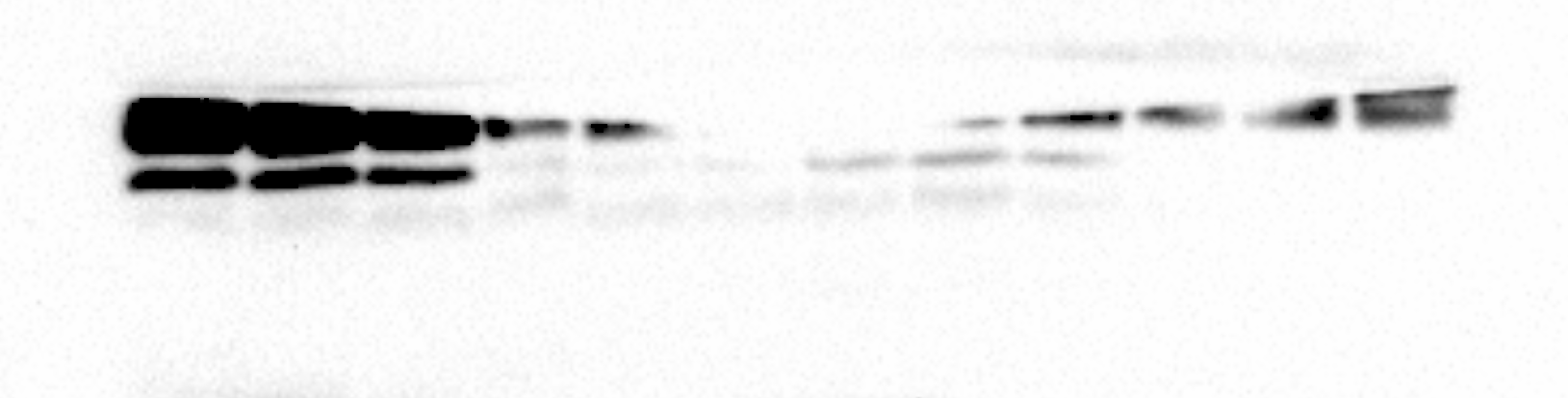

Supplement: Figure 2—figure supplement 3—source data 1. [file elife-82283-fig2-figsupp3-data1.zip › Fig2-SupFig3-Source data/EpiWAT_Oxphos_raw_high.tif]

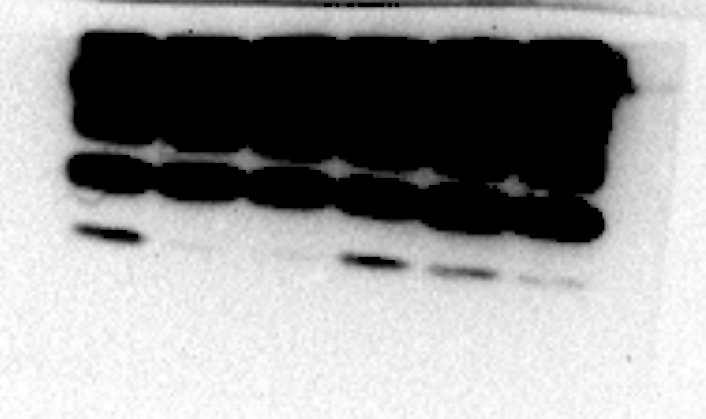

Supplement: Figure 2—figure supplement 3—source data 1. [file elife-82283-fig2-figsupp3-data1.zip › Fig2-SupFig3-Source data/Heart_Tomm20_raw.tif]

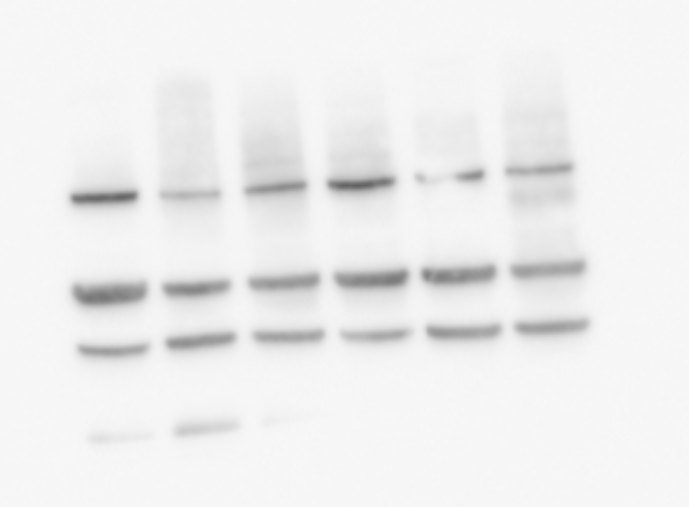

Supplement: Figure 2—figure supplement 3—source data 1. [file elife-82283-fig2-figsupp3-data1.zip › Fig2-SupFig3-Source data/Heart_Canx_raw.tif]

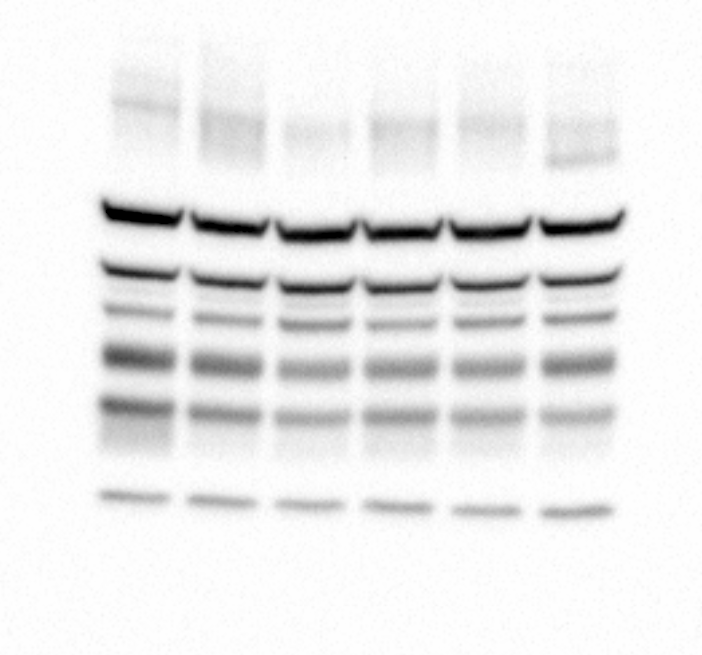

Supplement: Figure 2—figure supplement 3—source data 1. [file elife-82283-fig2-figsupp3-data1.zip › Fig2-SupFig3-Source data/Liver_Oxphos_raw.tif]

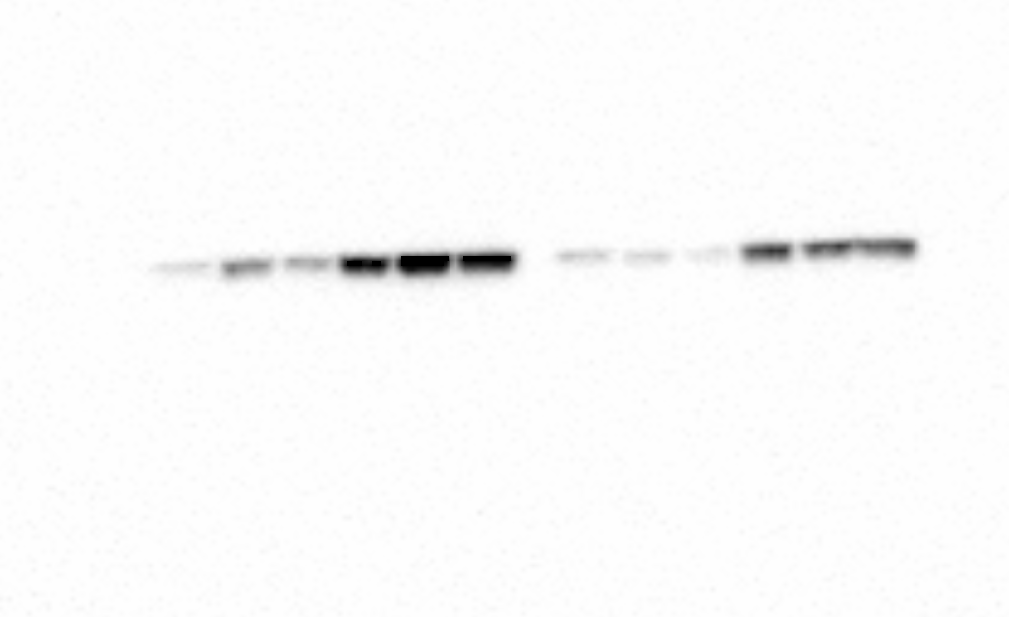

Supplement: Figure 4—source data 1. [file elife-82283-fig4-data1.zip › Fig4-Source data/BAT_PhosEif_raw.tif]

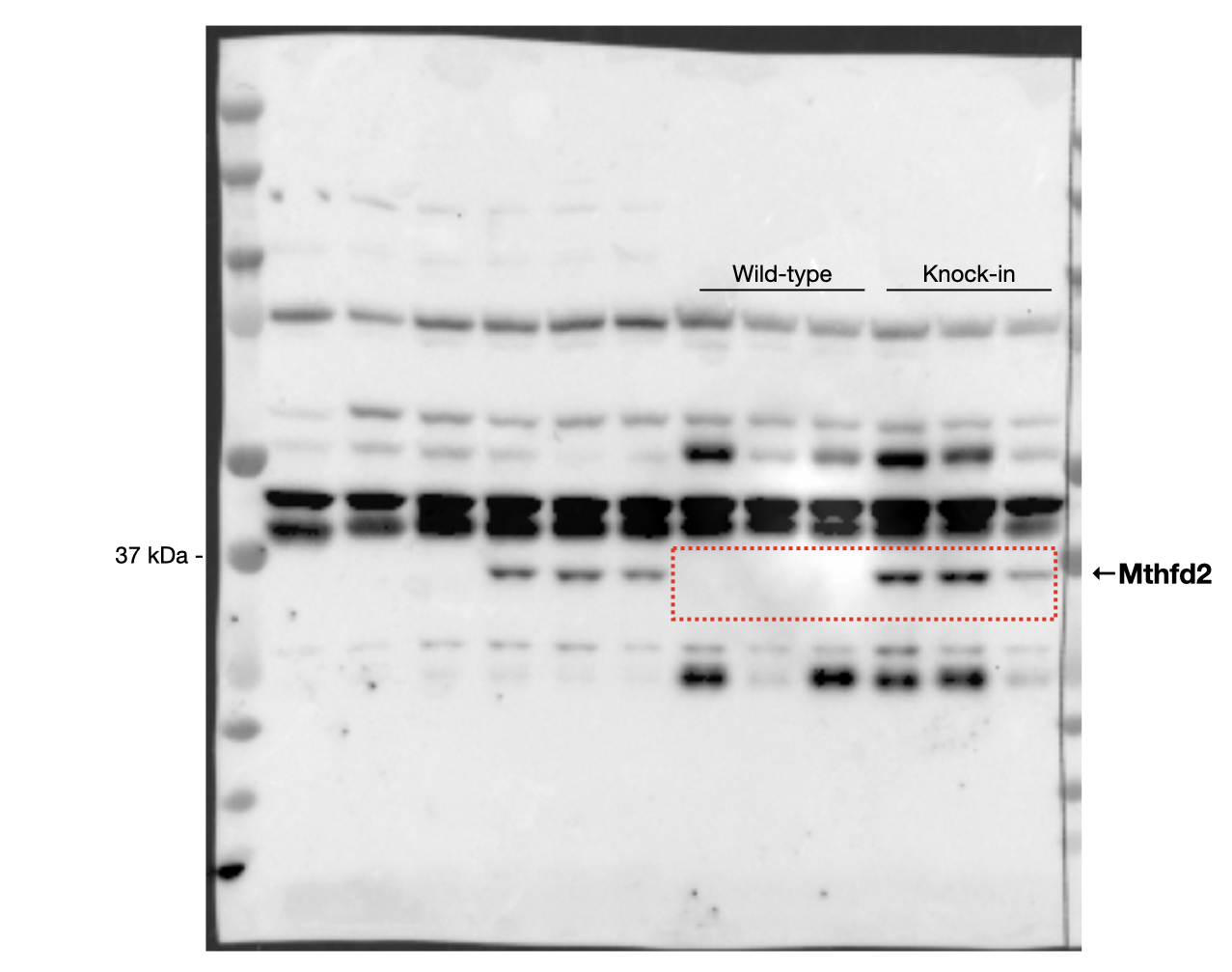

Supplement: Figure 4—source data 1. [file elife-82283-fig4-data1.zip › Fig4-Source data/BAT_Mthfd2_annotated.jpeg]

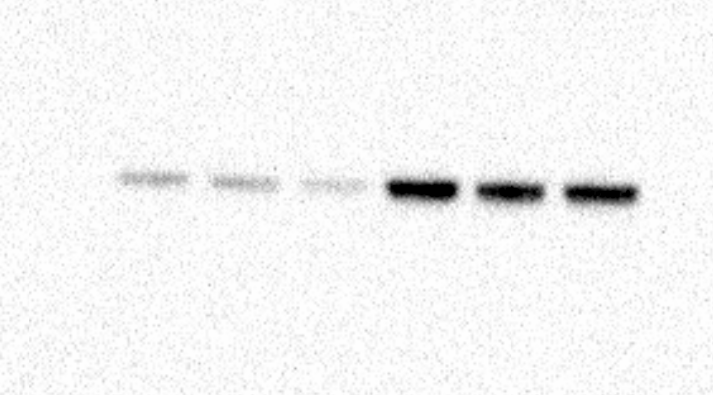

Supplement: Figure 4—source data 1. [file elife-82283-fig4-data1.zip › Fig4-Source data/EpiWAT_PhosEif_raw.tif]

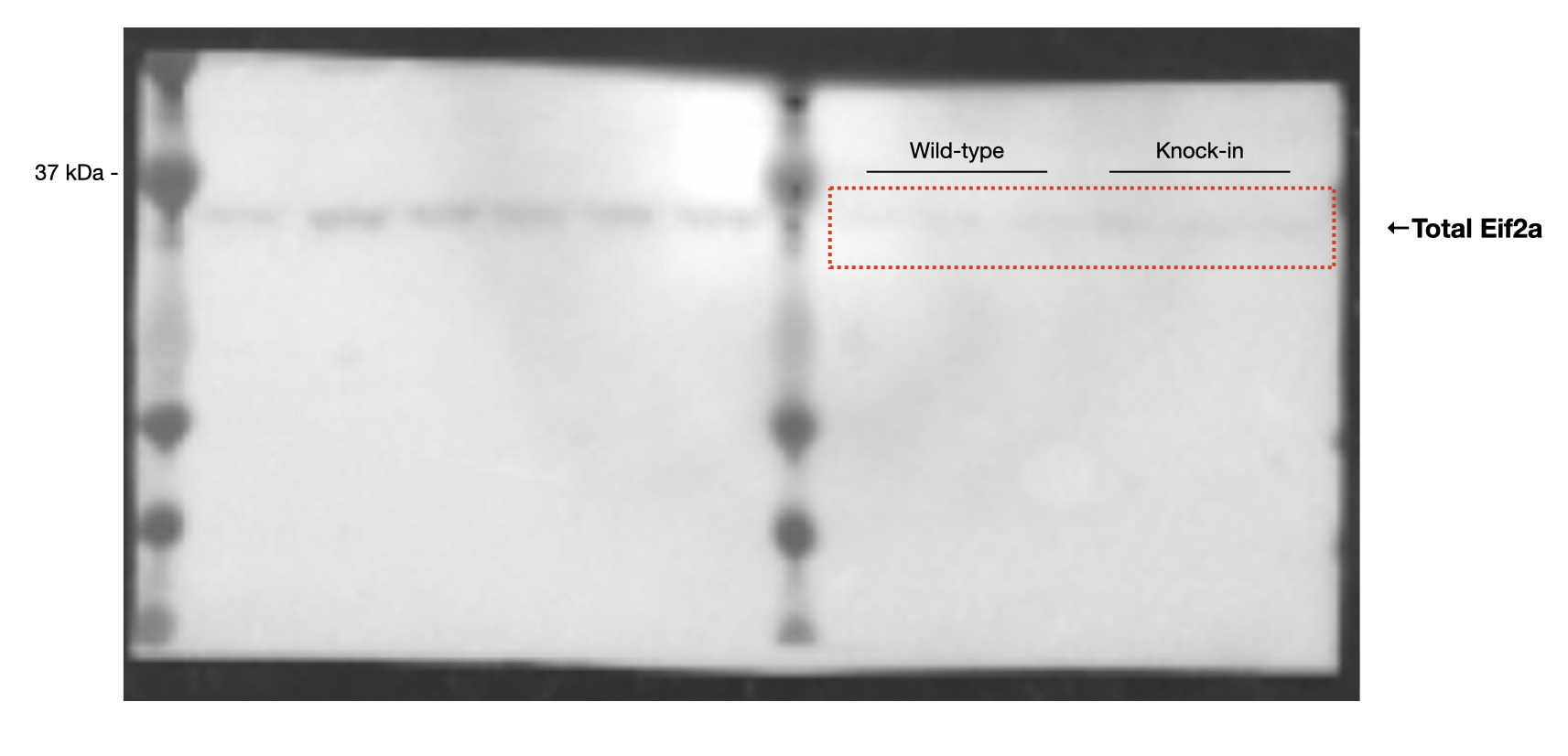

Supplement: Figure 4—source data 1. [file elife-82283-fig4-data1.zip › Fig4-Source data/BAT_TotEif_annotated.jpeg]

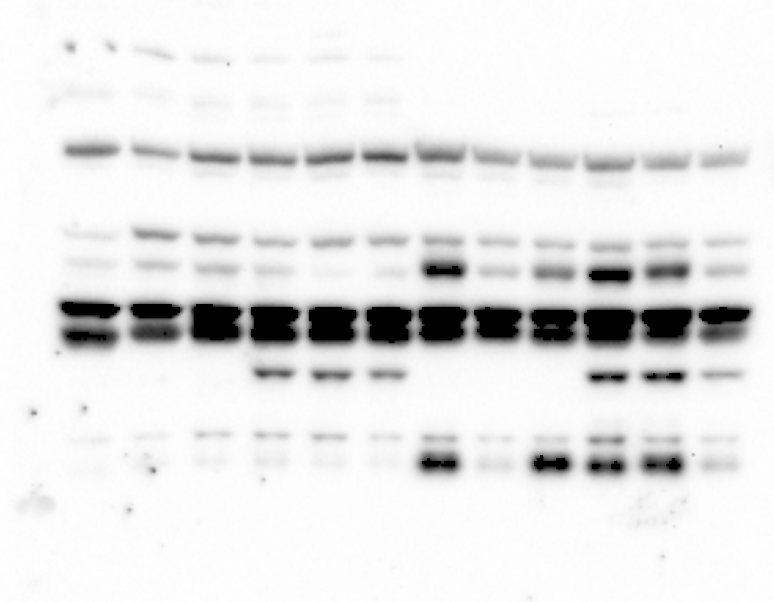

Supplement: Figure 4—source data 1. [file elife-82283-fig4-data1.zip › Fig4-Source data/BAT_Mthfd2_raw.tif]

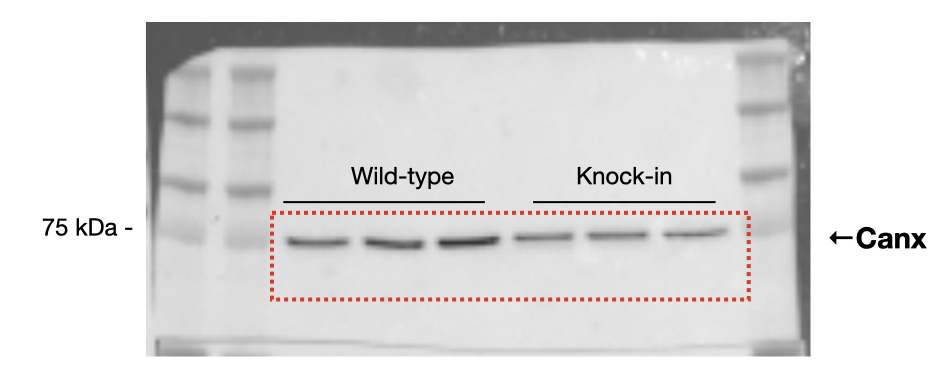

Supplement: Figure 4—source data 1. [file elife-82283-fig4-data1.zip › Fig4-Source data/Liver_Canx_annotated.jpeg]

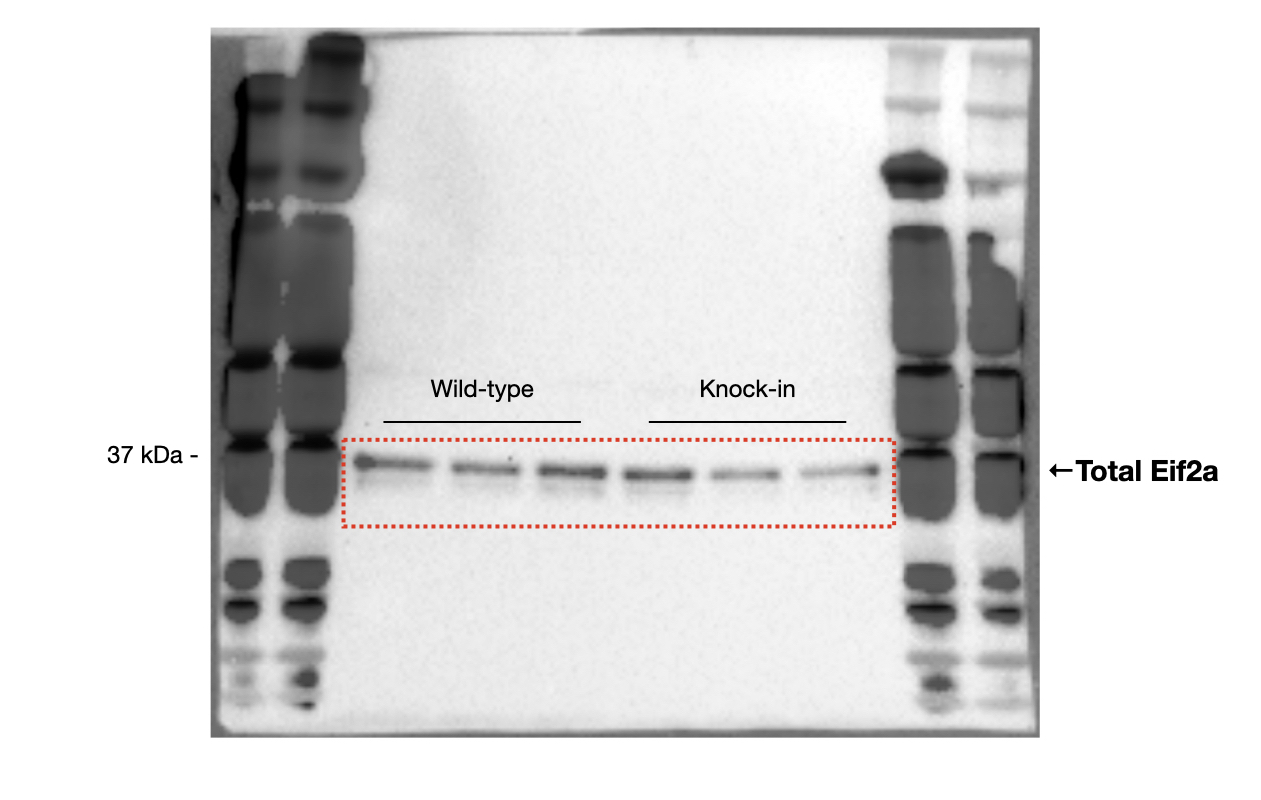

Supplement: Figure 4—source data 1. [file elife-82283-fig4-data1.zip › Fig4-Source data/Liver_TotEif_annotated.jpeg]

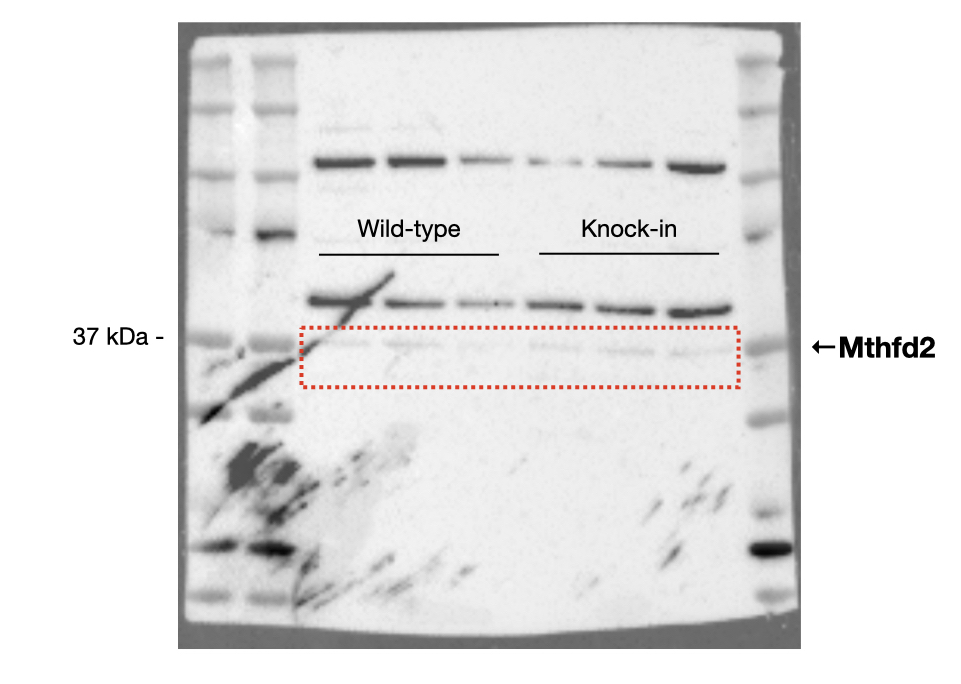

Supplement: Figure 4—source data 1. [file elife-82283-fig4-data1.zip › Fig4-Source data/Liver_Mthfd2_annotated.jpeg]

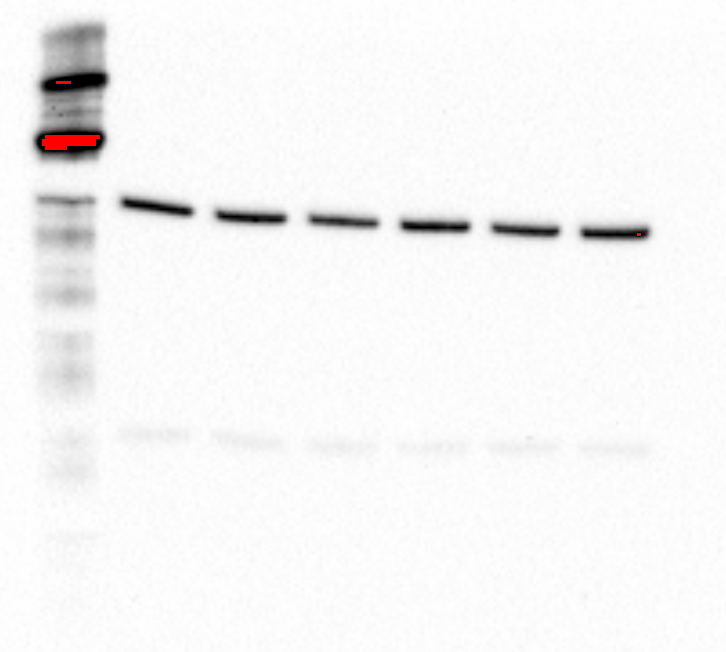

Supplement: Figure 4—source data 1. [file elife-82283-fig4-data1.zip › Fig4-Source data/EpiWAT_Canx_raw.tif]

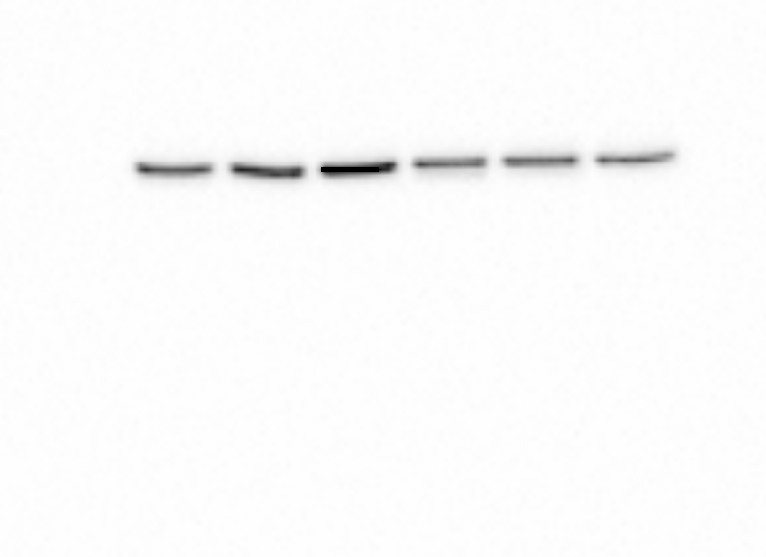

Supplement: Figure 4—source data 1. [file elife-82283-fig4-data1.zip › Fig4-Source data/Liver_Canx_raw.tif]

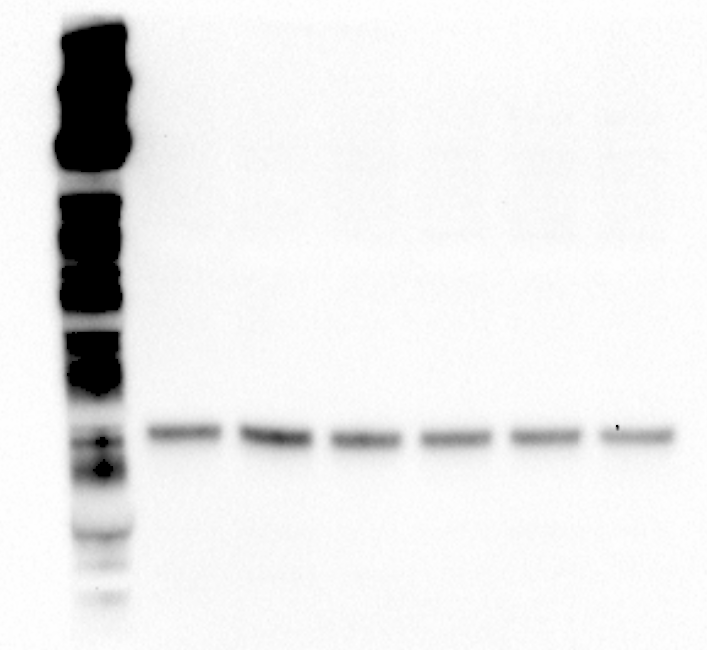

Supplement: Figure 4—source data 1. [file elife-82283-fig4-data1.zip › Fig4-Source data/EpiWAT_TotEif_raw.tif]

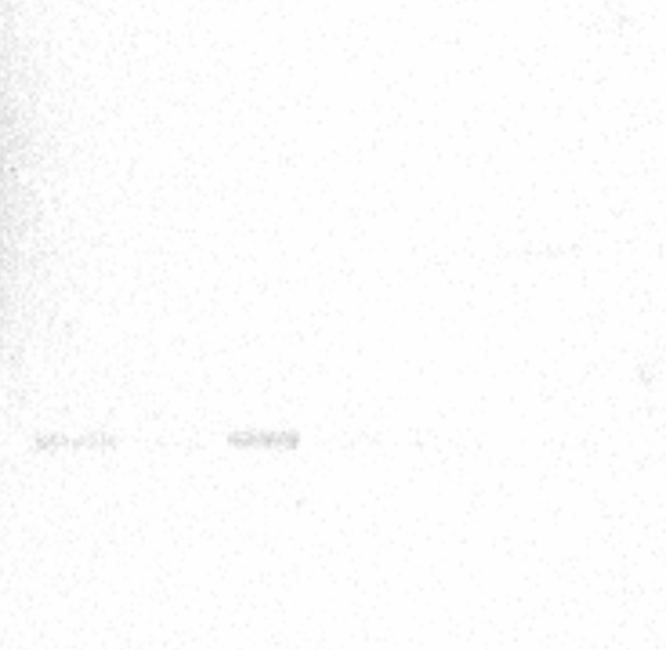

Supplement: Figure 4—source data 1. [file elife-82283-fig4-data1.zip › Fig4-Source data/Liver_PhosEif_raw.tif]

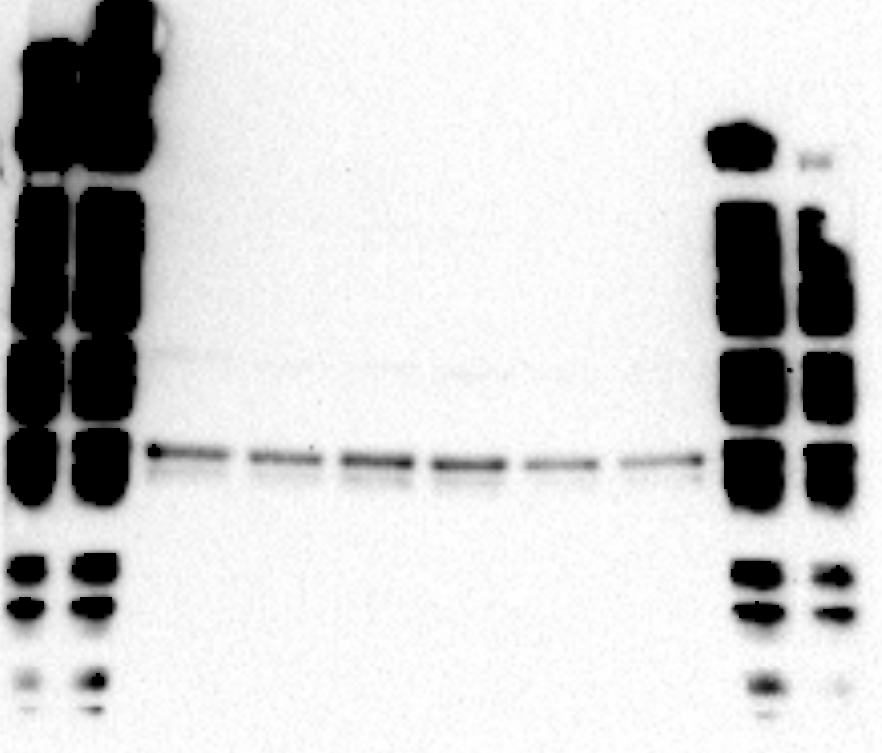

Supplement: Figure 4—source data 1. [file elife-82283-fig4-data1.zip › Fig4-Source data/Liver_TotEif_raw.tif]

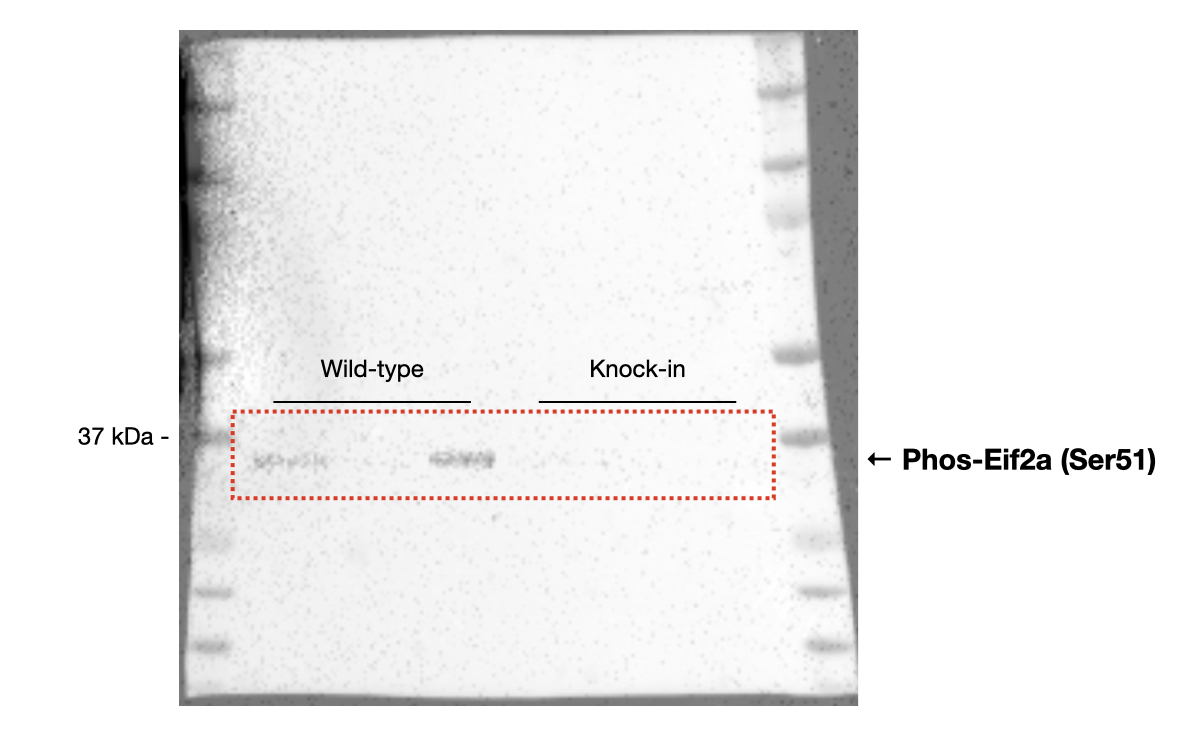

Supplement: Figure 4—source data 1. [file elife-82283-fig4-data1.zip › Fig4-Source data/Liver_PhosEif_annotated.jpeg]

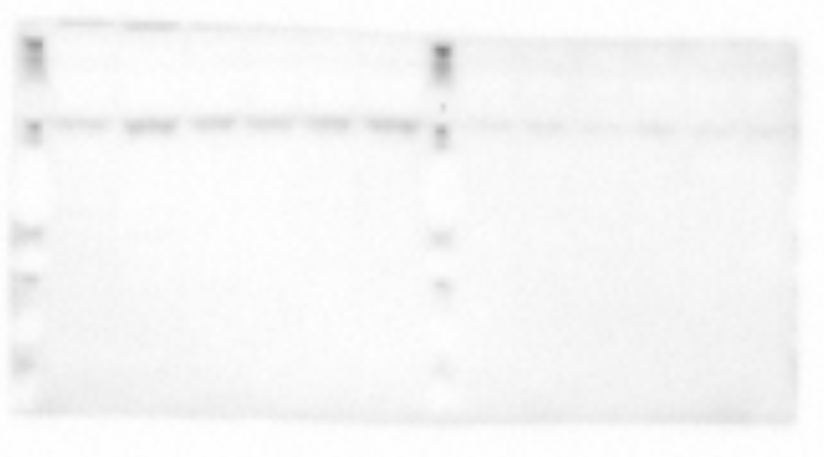

Supplement: Figure 4—source data 1. [file elife-82283-fig4-data1.zip › Fig4-Source data/BAT_TotEif_raw.tif]

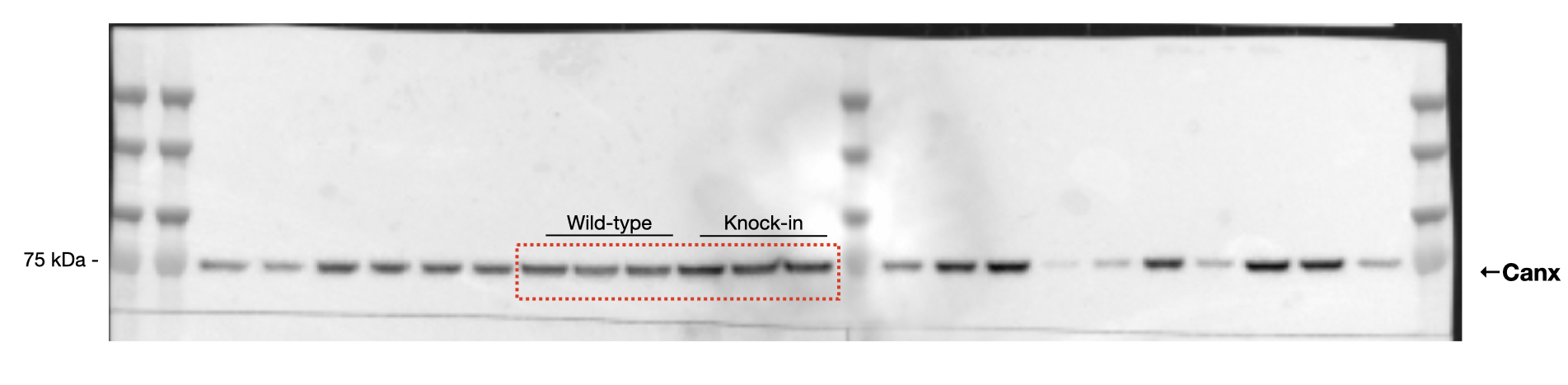

Supplement: Figure 4—source data 1. [file elife-82283-fig4-data1.zip › Fig4-Source data/BAT_Canx_annotated.jpeg]

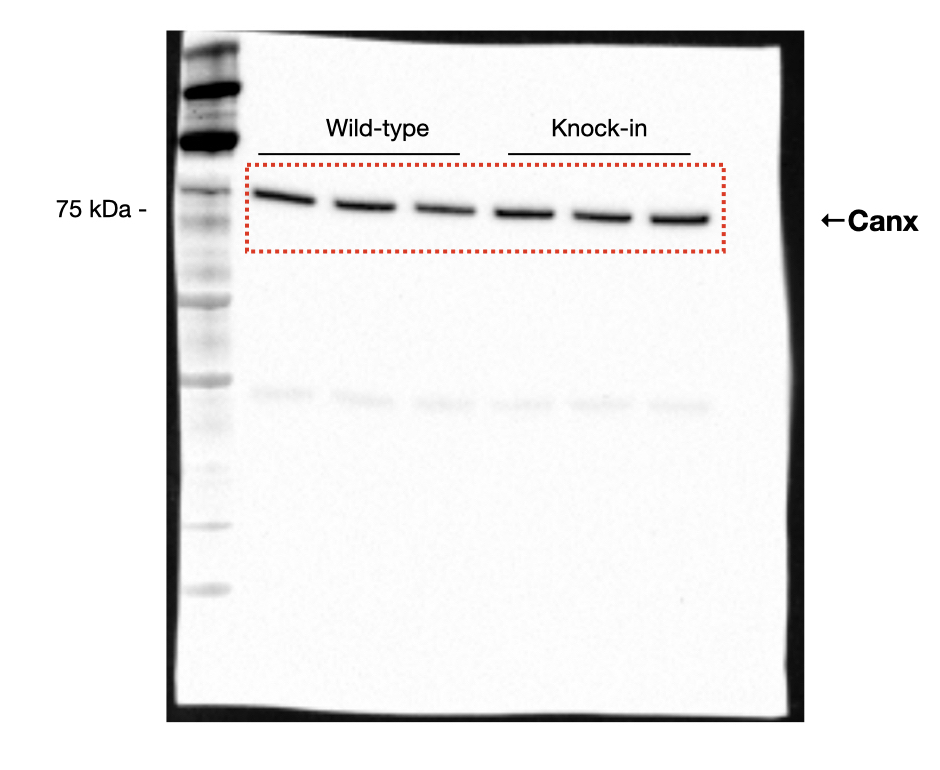

Supplement: Figure 4—source data 1. [file elife-82283-fig4-data1.zip › Fig4-Source data/EpiWAT_Canx_annotated.jpeg]

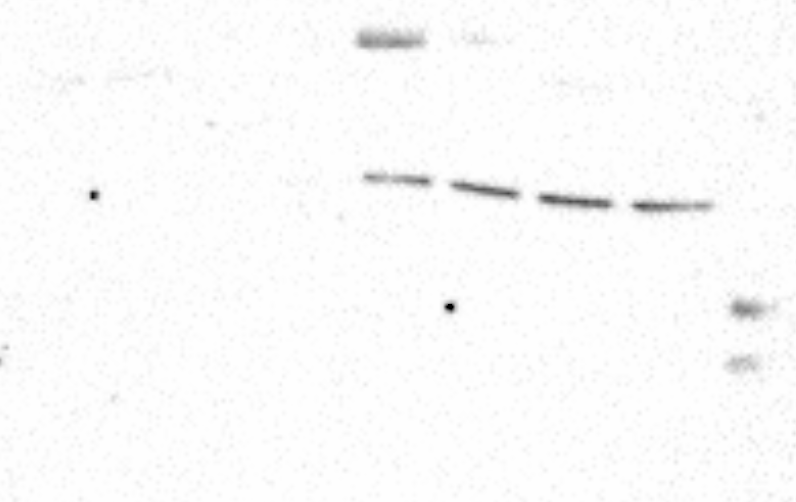

Supplement: Figure 4—source data 1. [file elife-82283-fig4-data1.zip › Fig4-Source data/EpiWAT_Mthfd2_raw.tif]

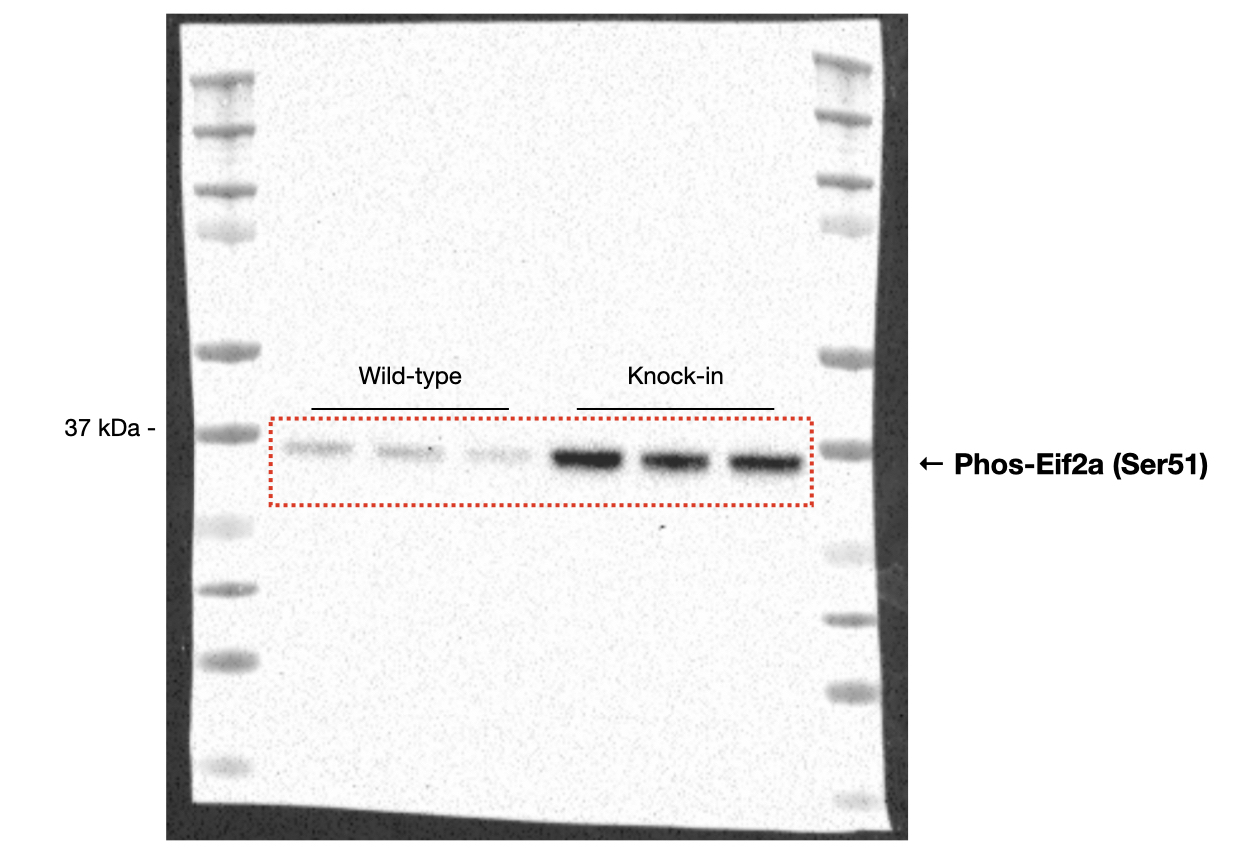

Supplement: Figure 4—source data 1. [file elife-82283-fig4-data1.zip › Fig4-Source data/EpiWAT_PhosEif_annotated.jpeg]

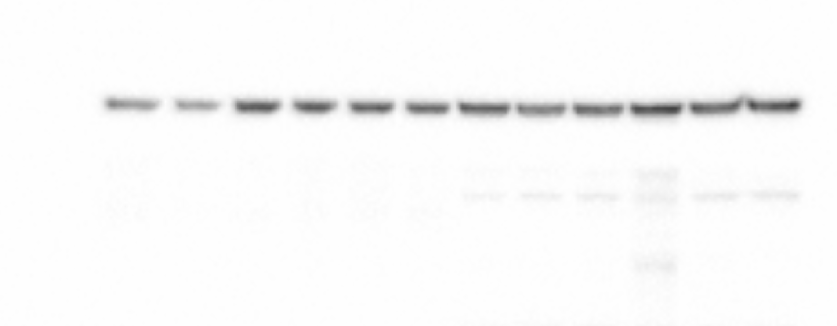

Supplement: Figure 4—source data 1. [file elife-82283-fig4-data1.zip › Fig4-Source data/BAT_Canx_raw.tif]

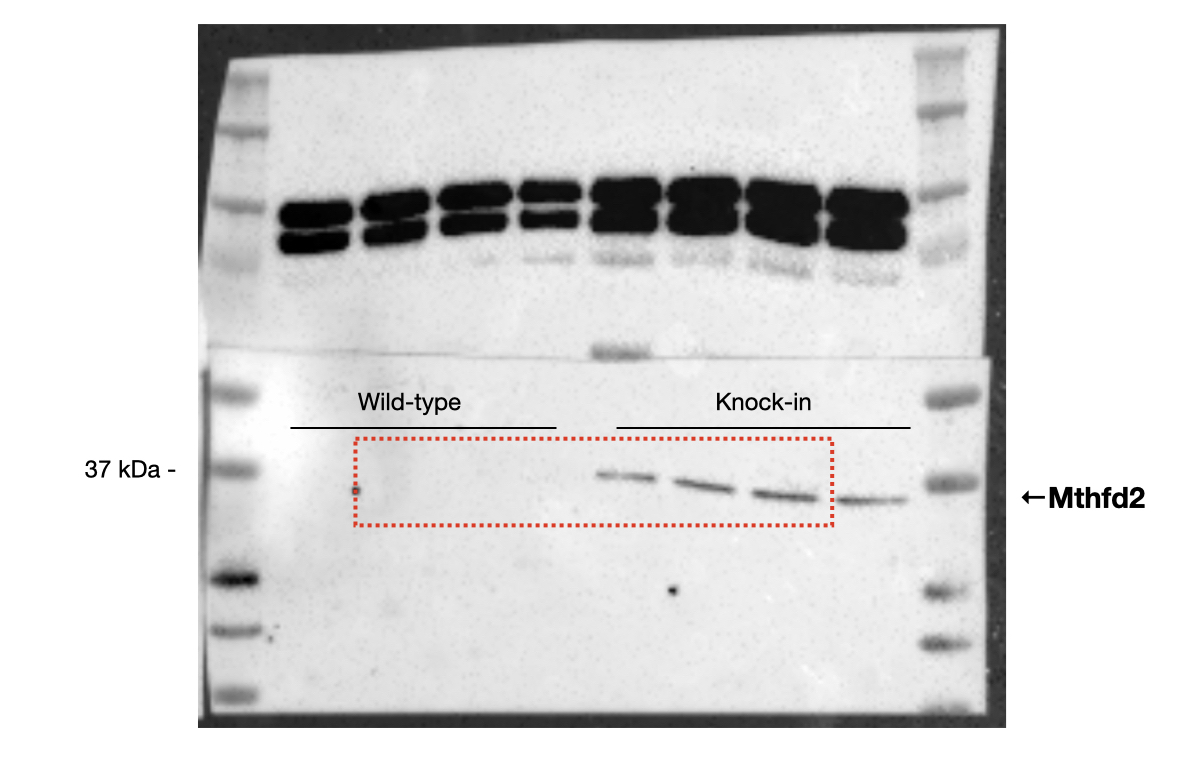

Supplement: Figure 4—source data 1. [file elife-82283-fig4-data1.zip › Fig4-Source data/EpiWAT_Mthfd2_annotated.jpeg]

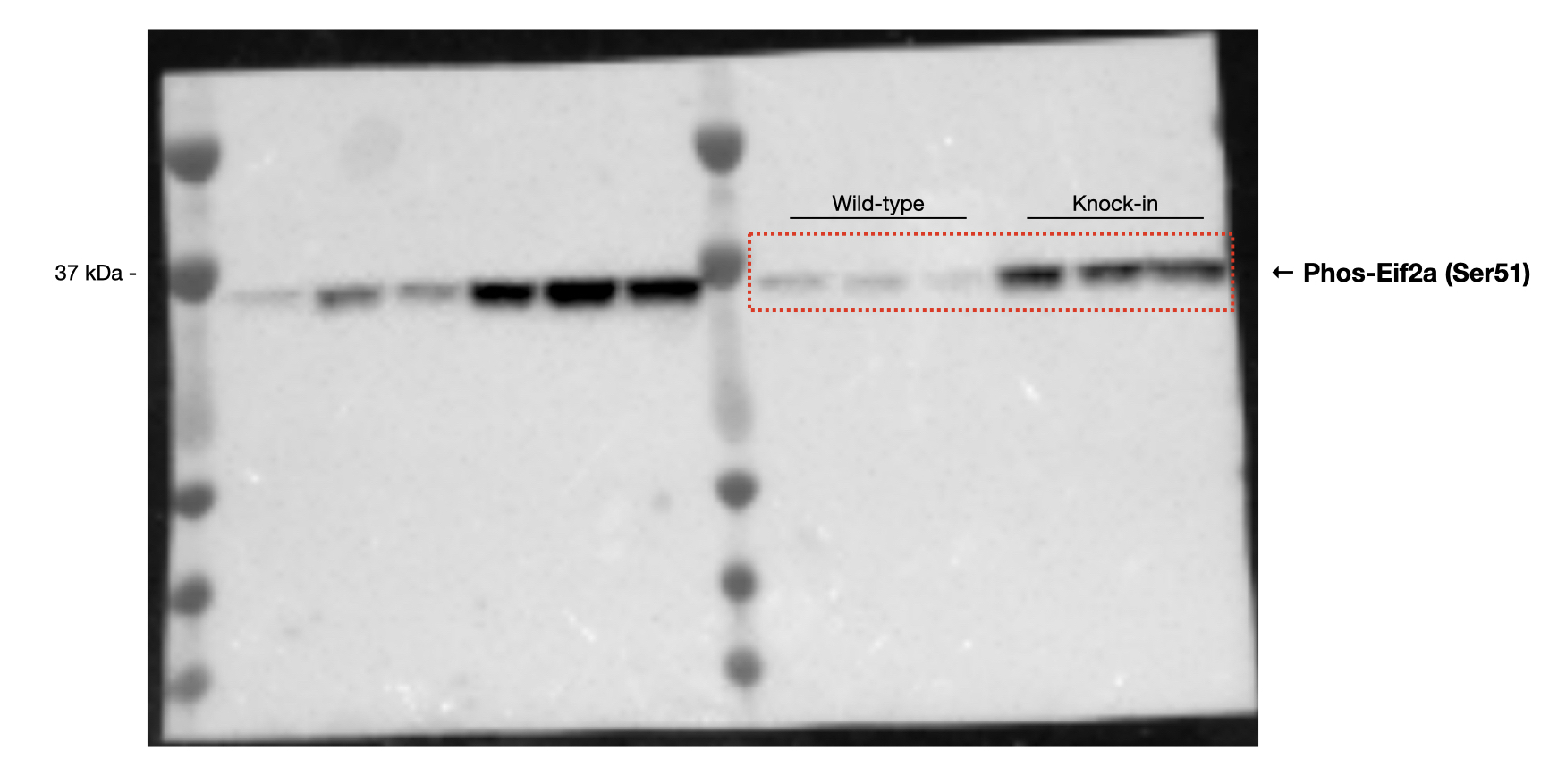

Supplement: Figure 4—source data 1. [file elife-82283-fig4-data1.zip › Fig4-Source data/BAT_PhosEif_annotated.jpeg]

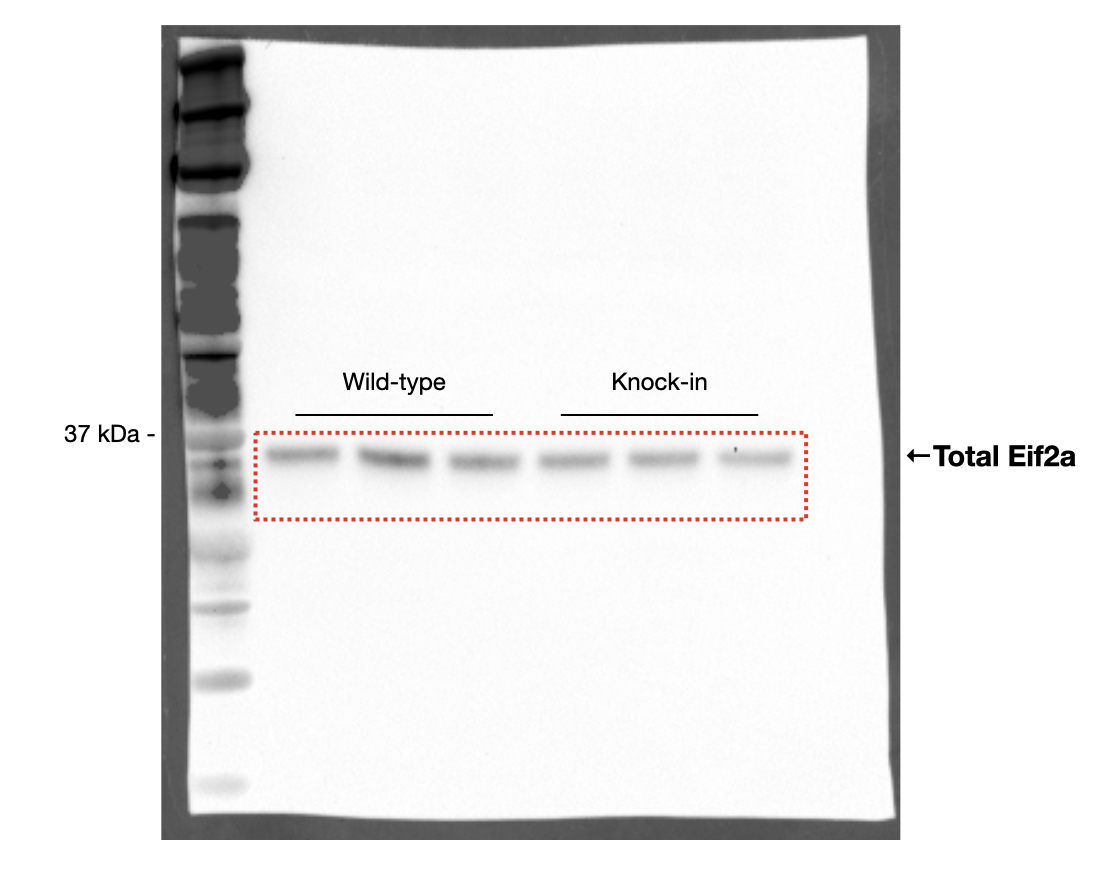

Supplement: Figure 4—source data 1. [file elife-82283-fig4-data1.zip › Fig4-Source data/EpiWAT_TotEif_annotated.jpeg]

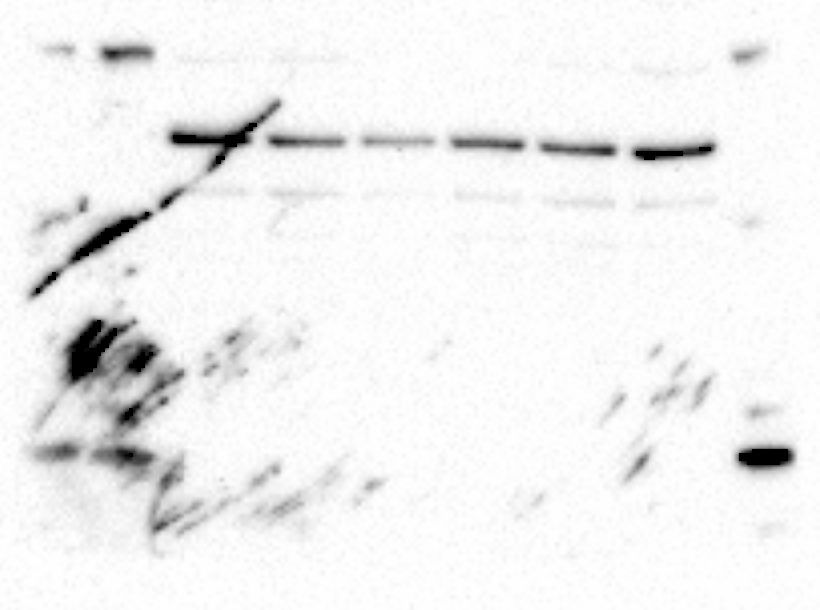

Supplement: Figure 4—source data 1. [file elife-82283-fig4-data1.zip › Fig4-Source data/Liver_Mthfd2_raw.tif]

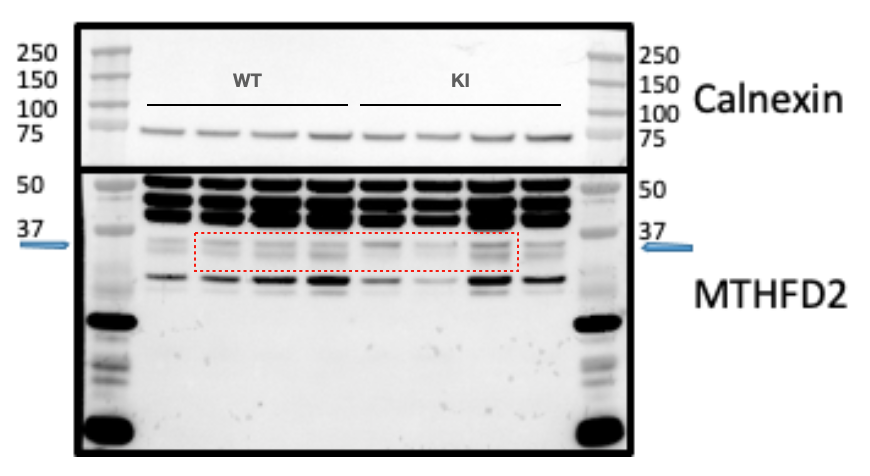

Supplement: Figure 4—figure supplement 1—source data 1. [file elife-82283-fig4-figsupp1-data1.zip › Fig4-SupFig1-Source data/Heart_Mthfd2_annotated.png]

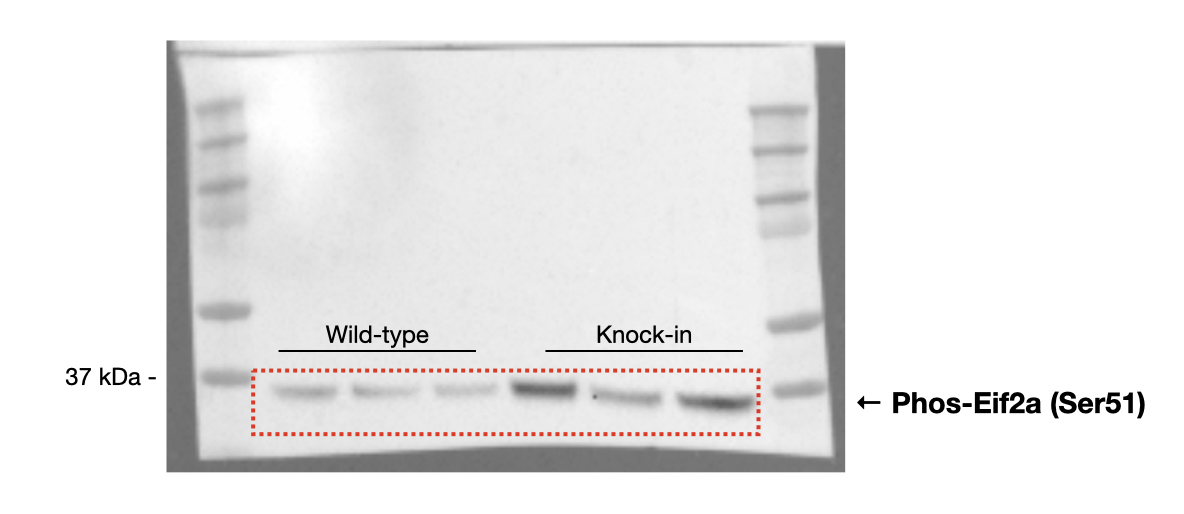

Supplement: Figure 4—figure supplement 1—source data 1. [file elife-82283-fig4-figsupp1-data1.zip › Fig4-SupFig1-Source data/IngWAT_PhosEif_annotated.jpeg]

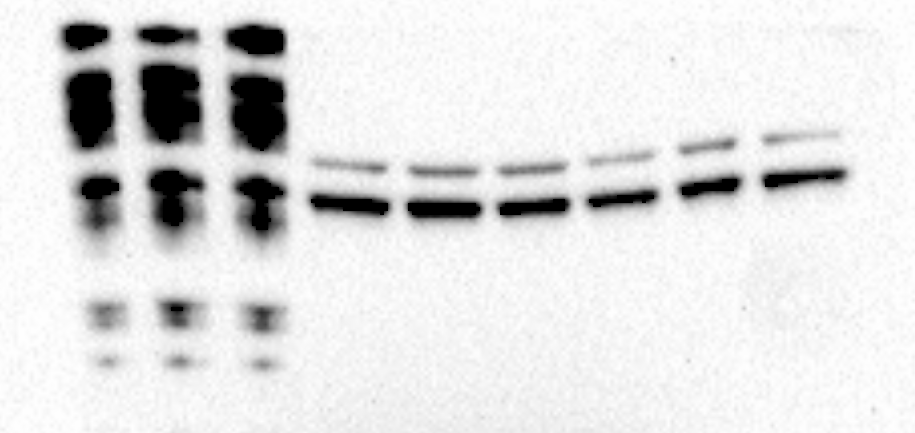

Supplement: Figure 4—figure supplement 1—source data 1. [file elife-82283-fig4-figsupp1-data1.zip › Fig4-SupFig1-Source data/SkelMusc_TotEif_raw.tif]

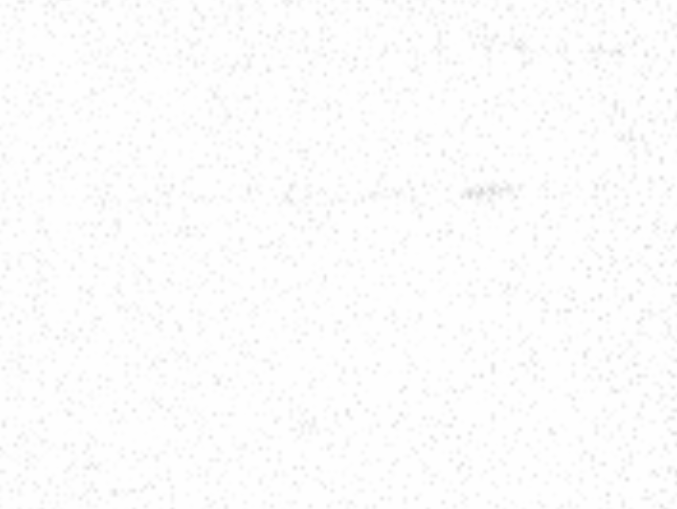

Supplement: Figure 4—figure supplement 1—source data 1. [file elife-82283-fig4-figsupp1-data1.zip › Fig4-SupFig1-Source data/SkelMusc_PhosEif_raw.tif]

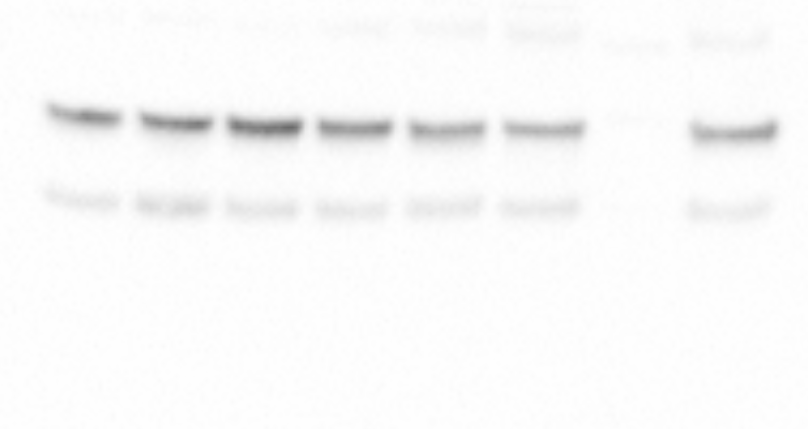

Supplement: Figure 4—figure supplement 1—source data 1. [file elife-82283-fig4-figsupp1-data1.zip › Fig4-SupFig1-Source data/IngWAT_TotEif_raw.tif]

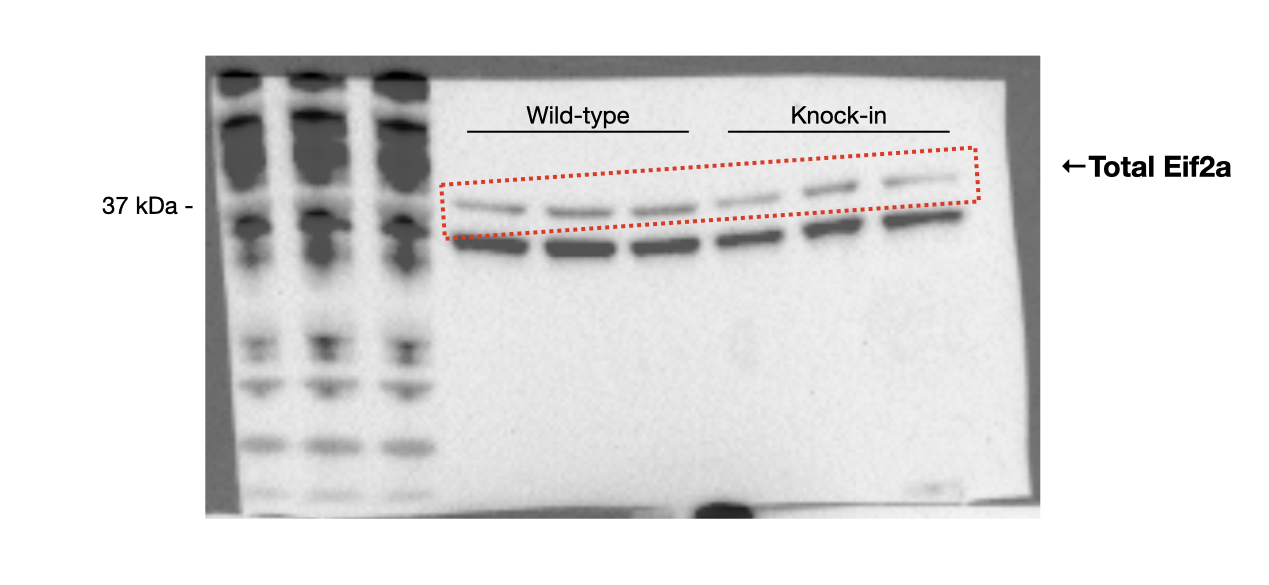

Supplement: Figure 4—figure supplement 1—source data 1. [file elife-82283-fig4-figsupp1-data1.zip › Fig4-SupFig1-Source data/SkelMusc_TotEif_annotated.jpeg]

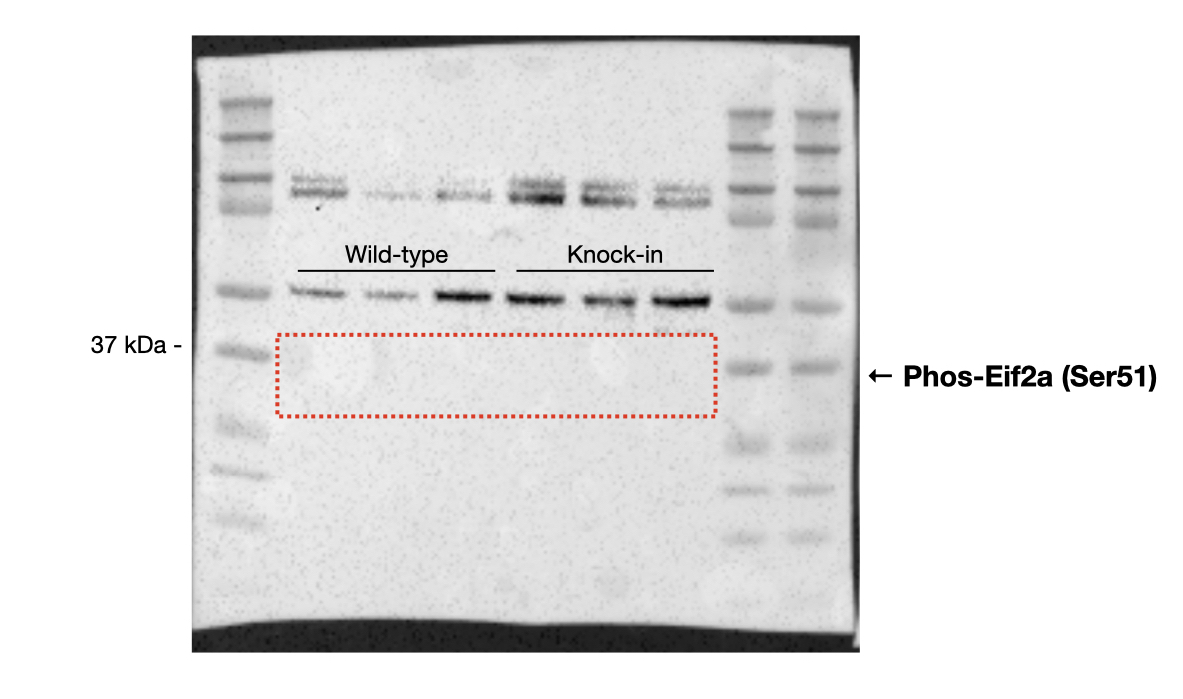

Supplement: Figure 4—figure supplement 1—source data 1. [file elife-82283-fig4-figsupp1-data1.zip › Fig4-SupFig1-Source data/Heart_PhosEif_annotated.jpeg]

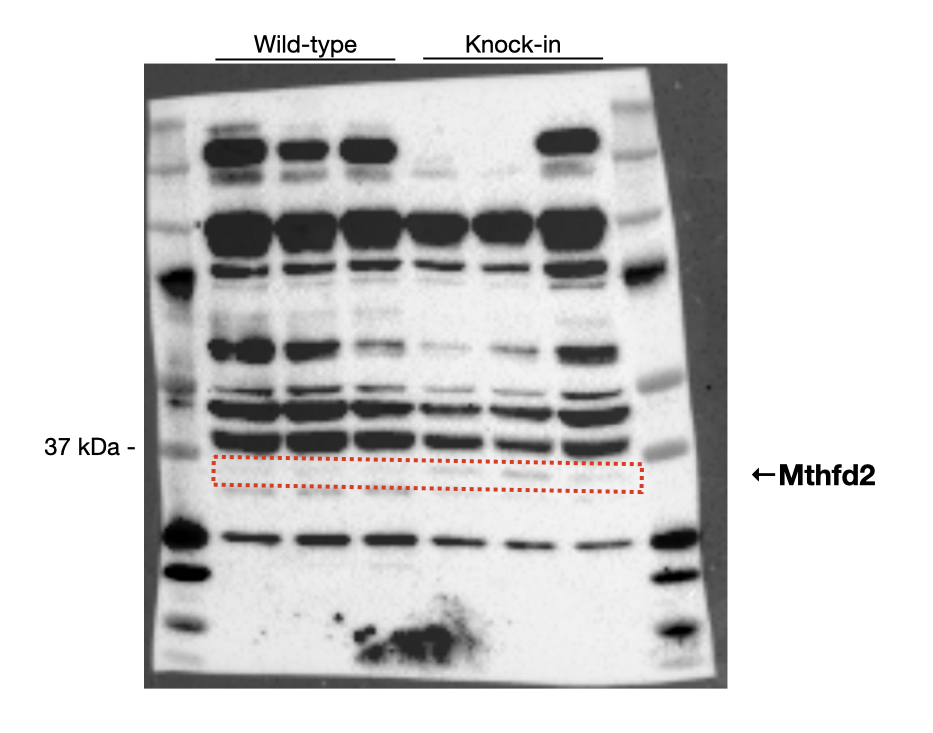

Supplement: Figure 4—figure supplement 1—source data 1. [file elife-82283-fig4-figsupp1-data1.zip › Fig4-SupFig1-Source data/SkelMusc_Mthfd2_annotated.jpeg]

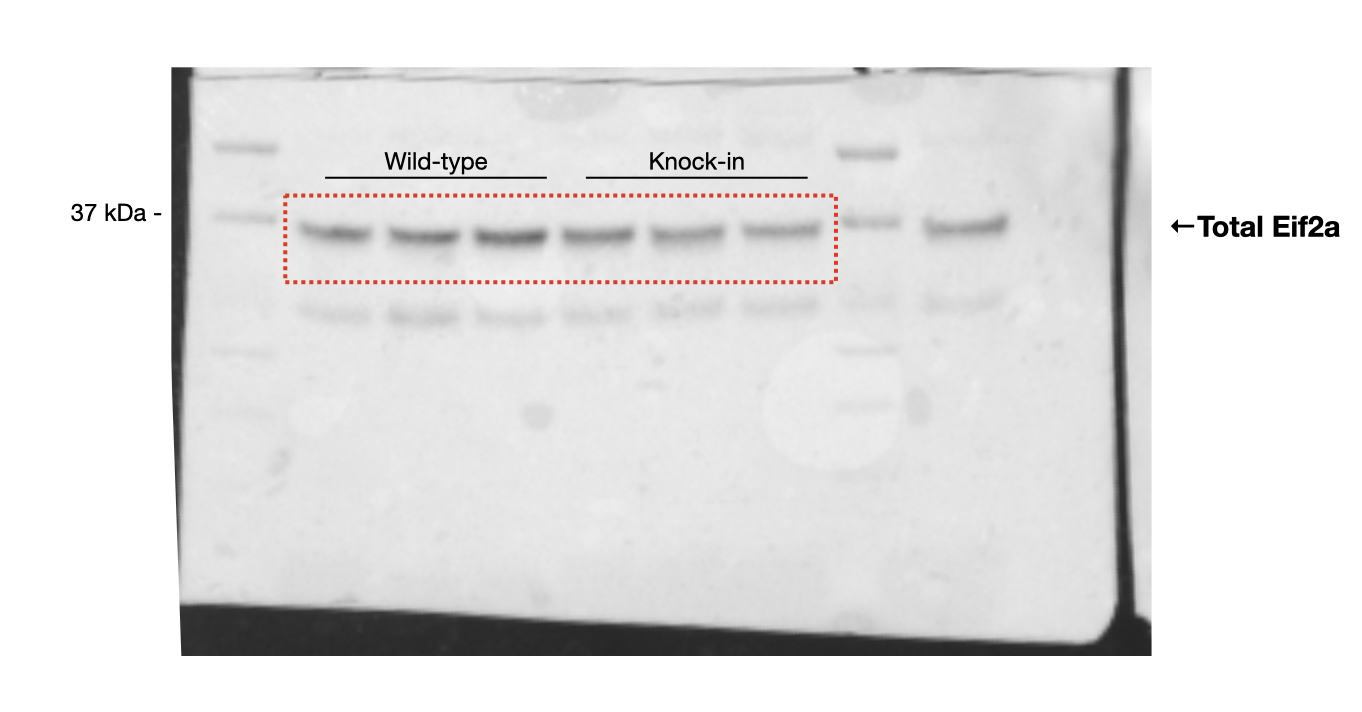

Supplement: Figure 4—figure supplement 1—source data 1. [file elife-82283-fig4-figsupp1-data1.zip › Fig4-SupFig1-Source data/IngWAT_TotEif_annotated.jpeg]

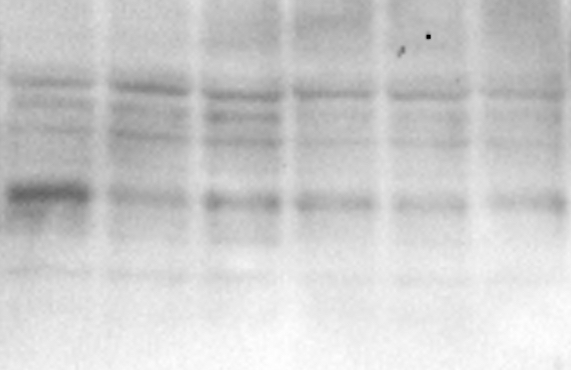

Supplement: Figure 4—figure supplement 1—source data 1. [file elife-82283-fig4-figsupp1-data1.zip › Fig4-SupFig1-Source data/Heart_TotEif_raw.jpg]

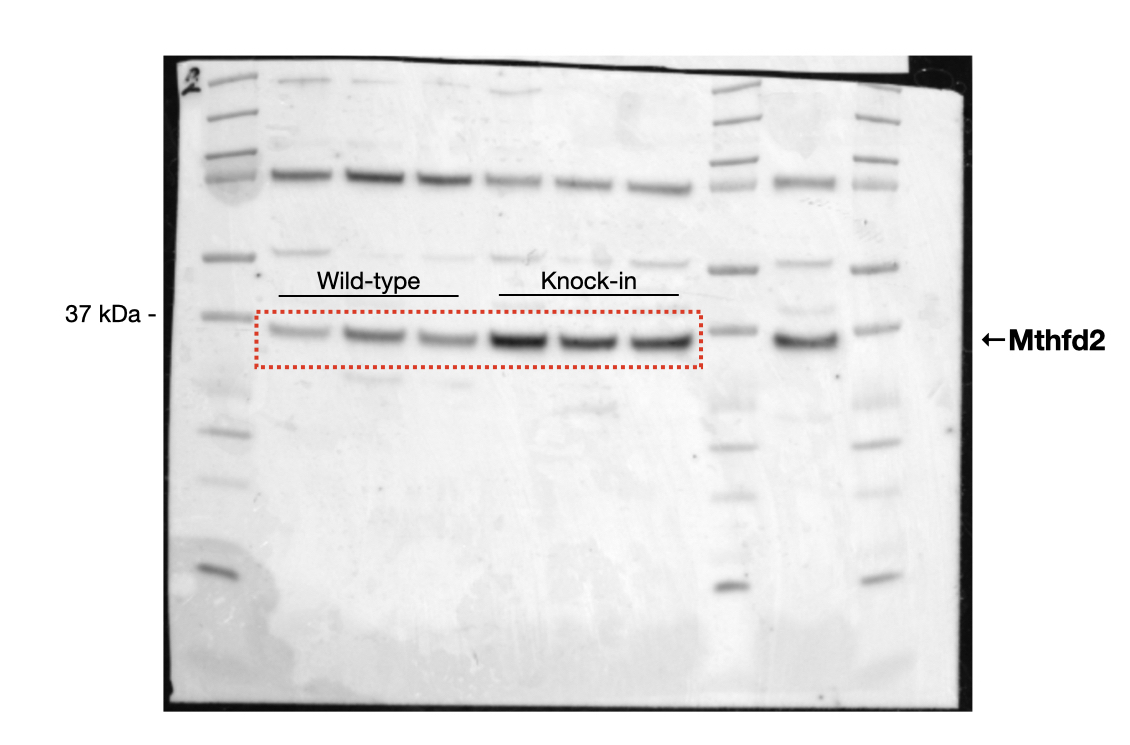

Supplement: Figure 4—figure supplement 1—source data 1. [file elife-82283-fig4-figsupp1-data1.zip › Fig4-SupFig1-Source data/IngWAT_Mthfd2_annotated.jpeg]

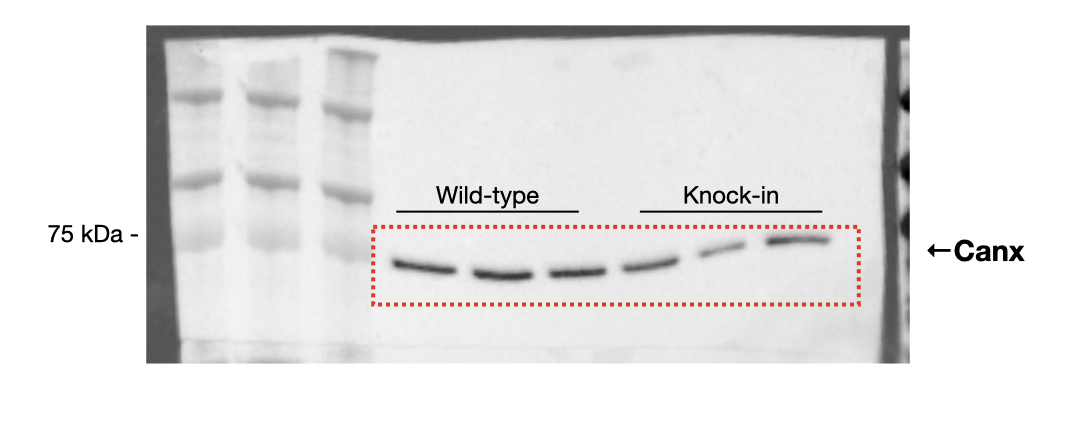

Supplement: Figure 4—figure supplement 1—source data 1. [file elife-82283-fig4-figsupp1-data1.zip › Fig4-SupFig1-Source data/SkelMusc_Canx_annotated.jpeg]

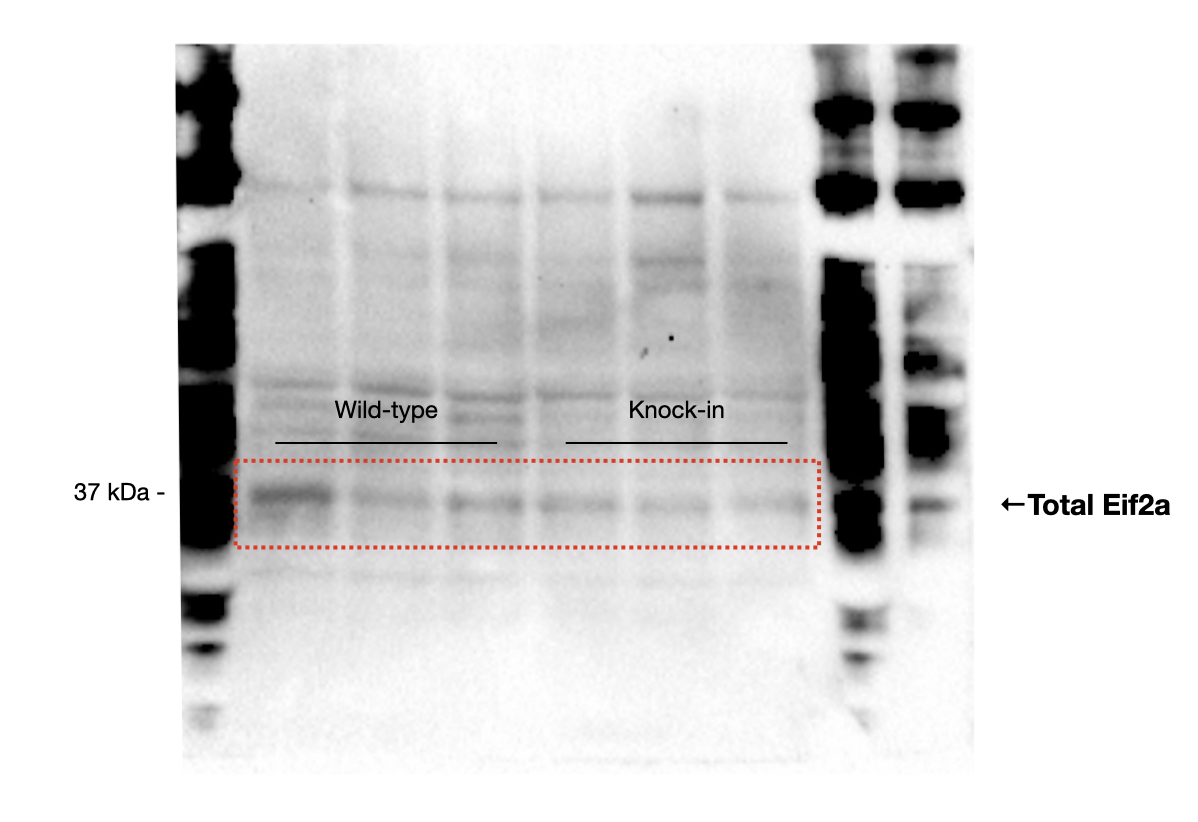

Supplement: Figure 4—figure supplement 1—source data 1. [file elife-82283-fig4-figsupp1-data1.zip › Fig4-SupFig1-Source data/Heart_TotEif_annotated.jpeg]

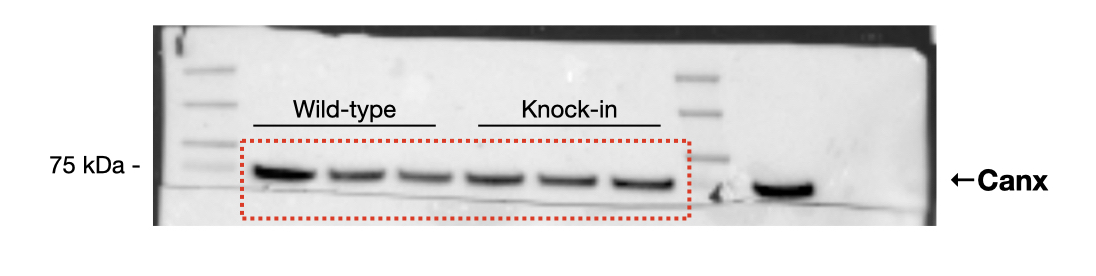

Supplement: Figure 4—figure supplement 1—source data 1. [file elife-82283-fig4-figsupp1-data1.zip › Fig4-SupFig1-Source data/IngWAT_Canx_annotated.jpeg]

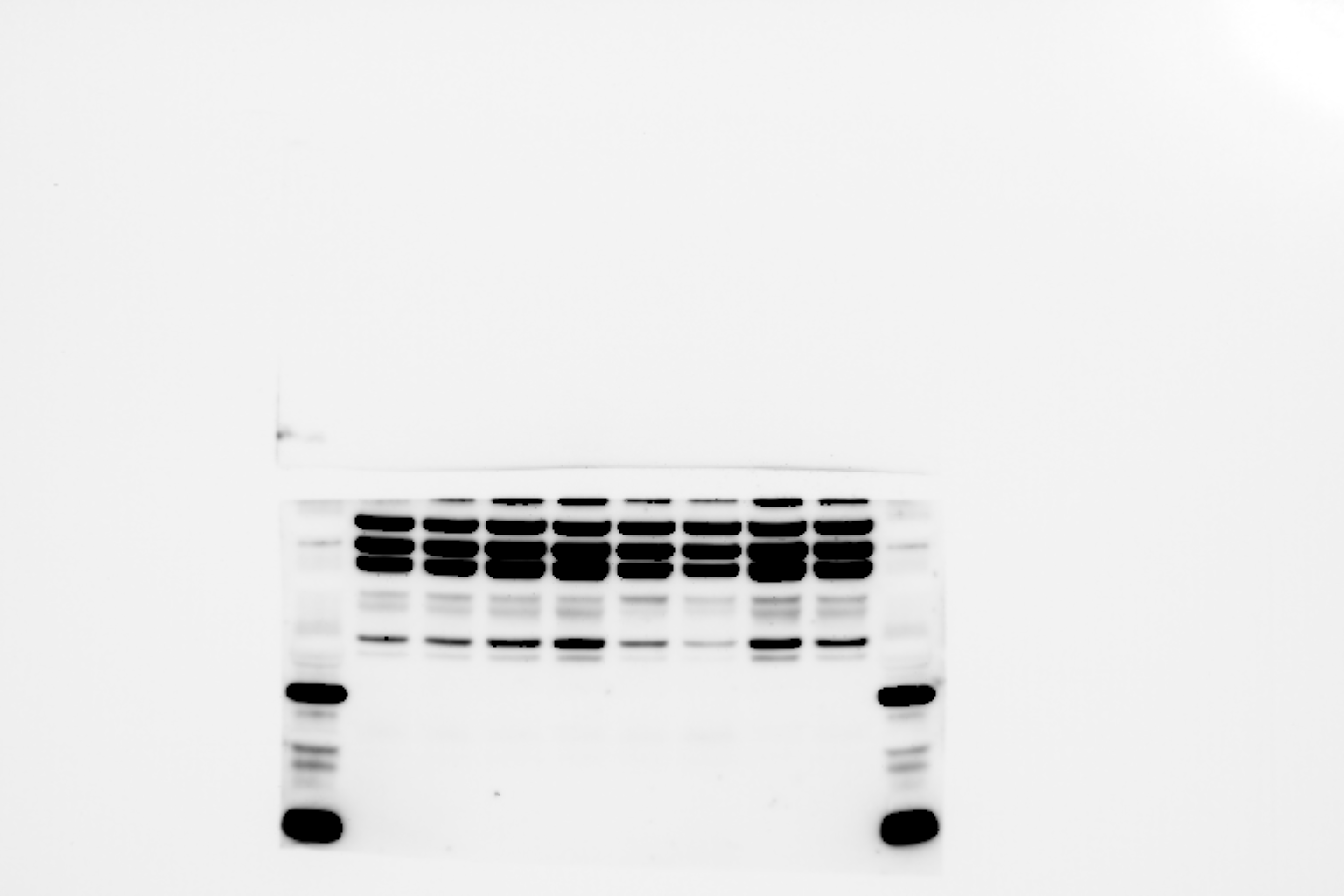

Supplement: Figure 4—figure supplement 1—source data 1. [file elife-82283-fig4-figsupp1-data1.zip › Fig4-SupFig1-Source data/Heart_Mthfd2_raw.tif]

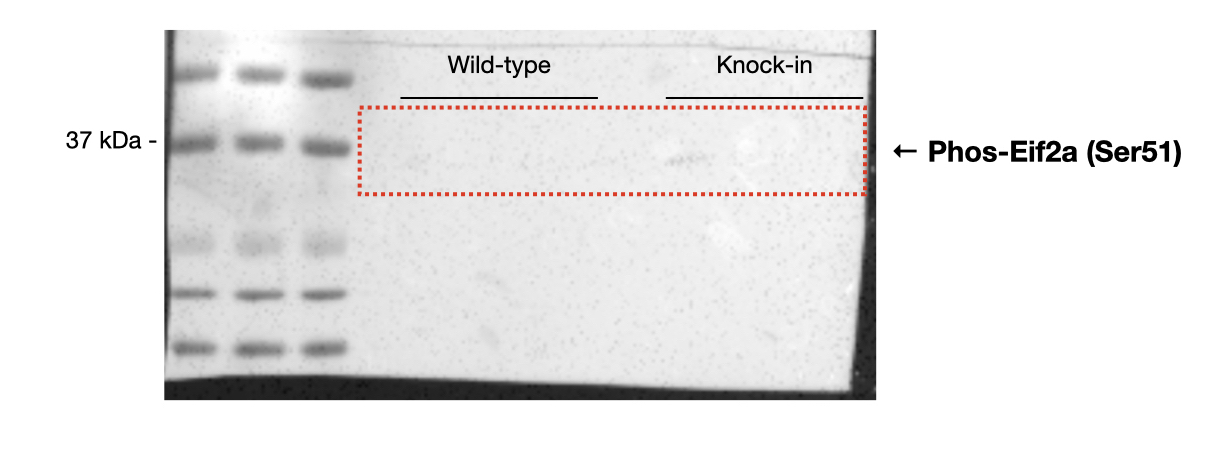

Supplement: Figure 4—figure supplement 1—source data 1. [file elife-82283-fig4-figsupp1-data1.zip › Fig4-SupFig1-Source data/SkelMusc_PhosEif_annotated.jpeg]

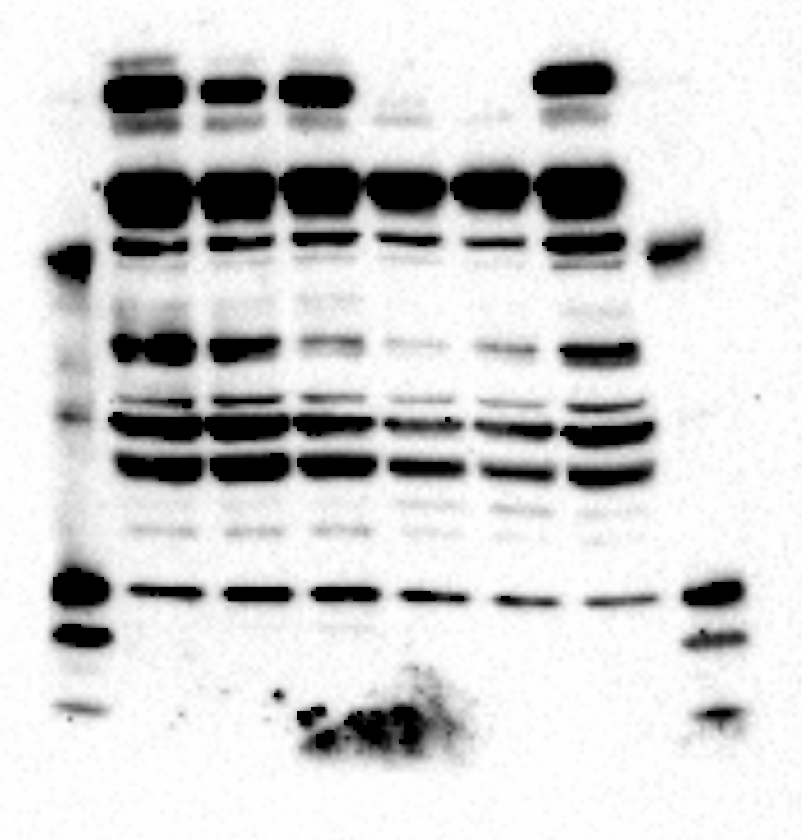

Supplement: Figure 4—figure supplement 1—source data 1. [file elife-82283-fig4-figsupp1-data1.zip › Fig4-SupFig1-Source data/SkelMusc_Mthfd2_raw.tif]

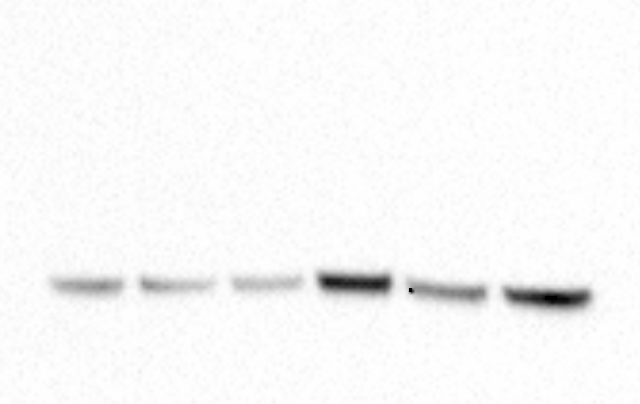

Supplement: Figure 4—figure supplement 1—source data 1. [file elife-82283-fig4-figsupp1-data1.zip › Fig4-SupFig1-Source data/IngWAT_PhosEif_raw.tif]

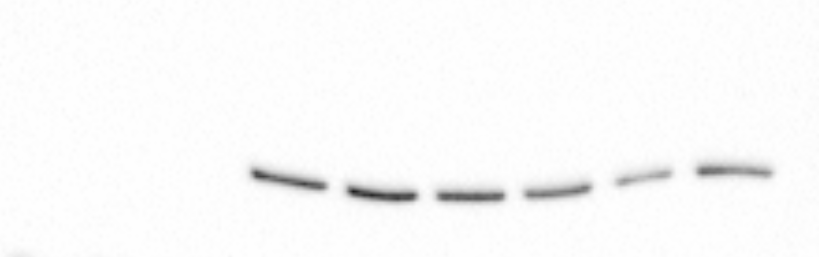

Supplement: Figure 4—figure supplement 1—source data 1. [file elife-82283-fig4-figsupp1-data1.zip › Fig4-SupFig1-Source data/SkelMusc_Canx_raw.tif]

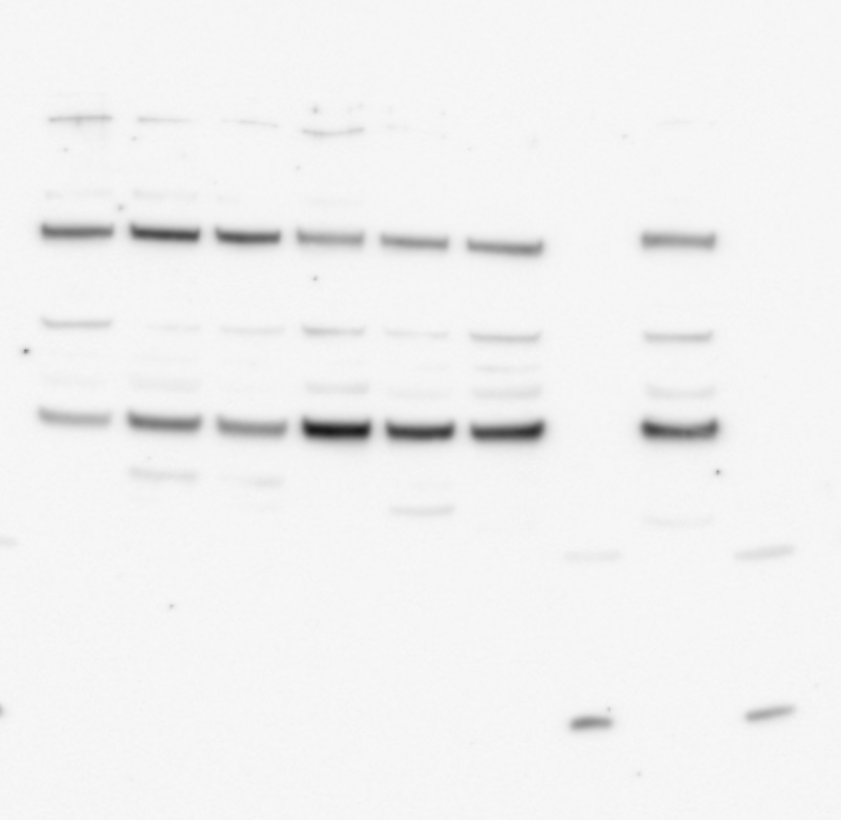

Supplement: Figure 4—figure supplement 1—source data 1. [file elife-82283-fig4-figsupp1-data1.zip › Fig4-SupFig1-Source data/IngWAT_Mthfd2_raw.tif]

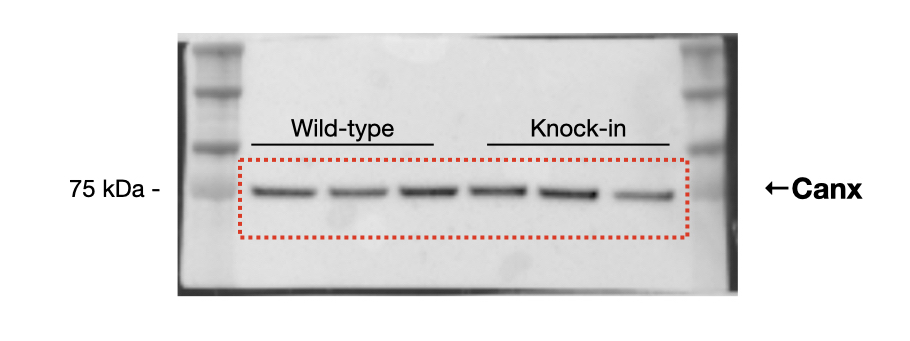

Supplement: Figure 4—figure supplement 1—source data 1. [file elife-82283-fig4-figsupp1-data1.zip › Fig4-SupFig1-Source data/Heart_Canx_annotated.jpeg]

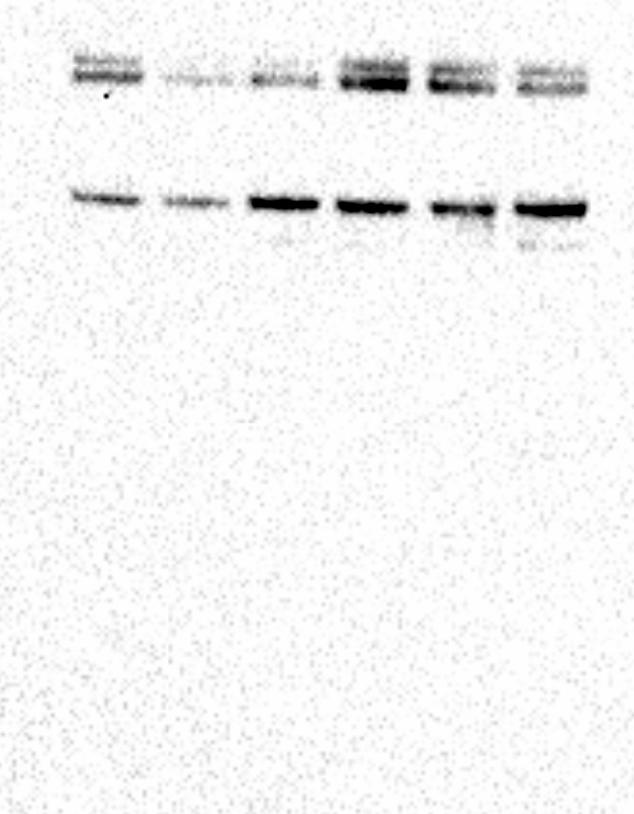

Supplement: Figure 4—figure supplement 1—source data 1. [file elife-82283-fig4-figsupp1-data1.zip › Fig4-SupFig1-Source data/Heart_PhosEif_raw.tif]

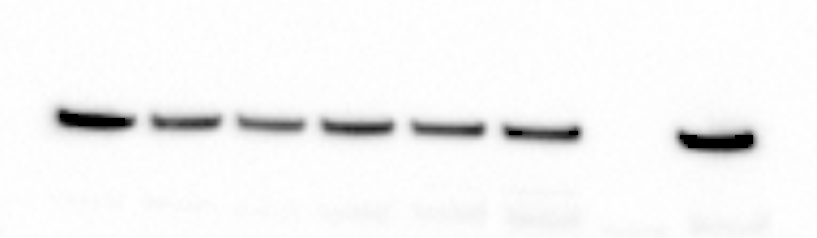

Supplement: Figure 4—figure supplement 1—source data 1. [file elife-82283-fig4-figsupp1-data1.zip › Fig4-SupFig1-Source data/IngWAT_Canx_raw.tif]

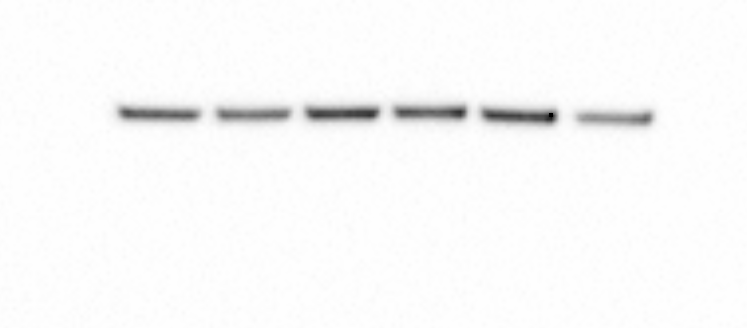

Supplement: Figure 4—figure supplement 1—source data 1. [file elife-82283-fig4-figsupp1-data1.zip › Fig4-SupFig1-Source data/Heart_Canx_raw.tif]

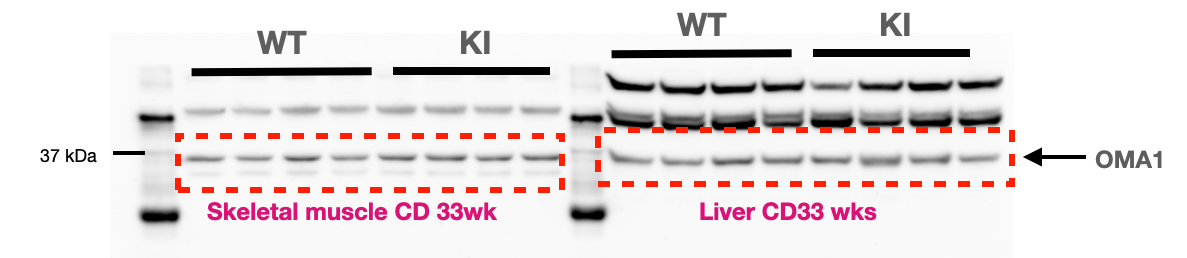

Supplement: Figure 4—figure supplement 3—source data 1. [file elife-82283-fig4-figsupp3-data1.zip › Fig4-SupFig3-Source data/F4S3_LiverMusc_Oma.png]

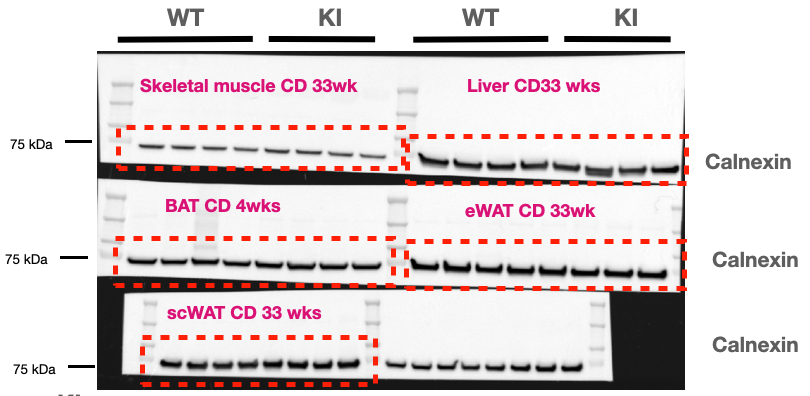

Supplement: Figure 4—figure supplement 3—source data 1. [file elife-82283-fig4-figsupp3-data1.zip › Fig4-SupFig3-Source data/F4S3_LiverMusc_Canx.png]

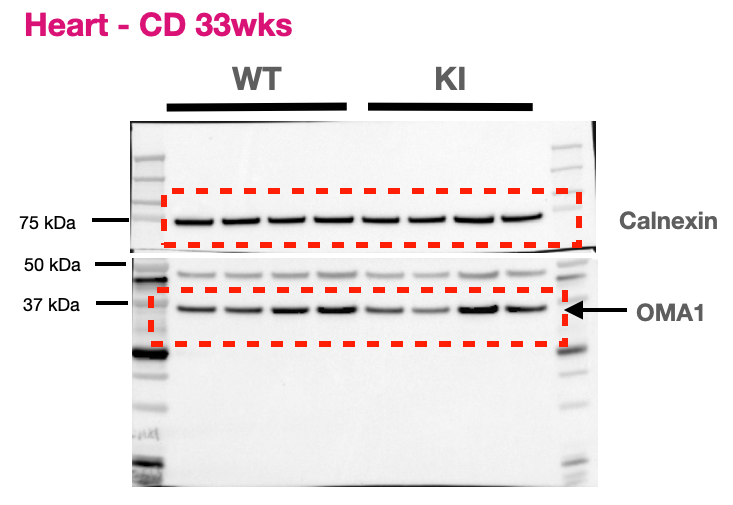

Supplement: Figure 4—figure supplement 3—source data 1. [file elife-82283-fig4-figsupp3-data1.zip › Fig4-SupFig3-Source data/F4S3_Heart_Oma-Canx.png]

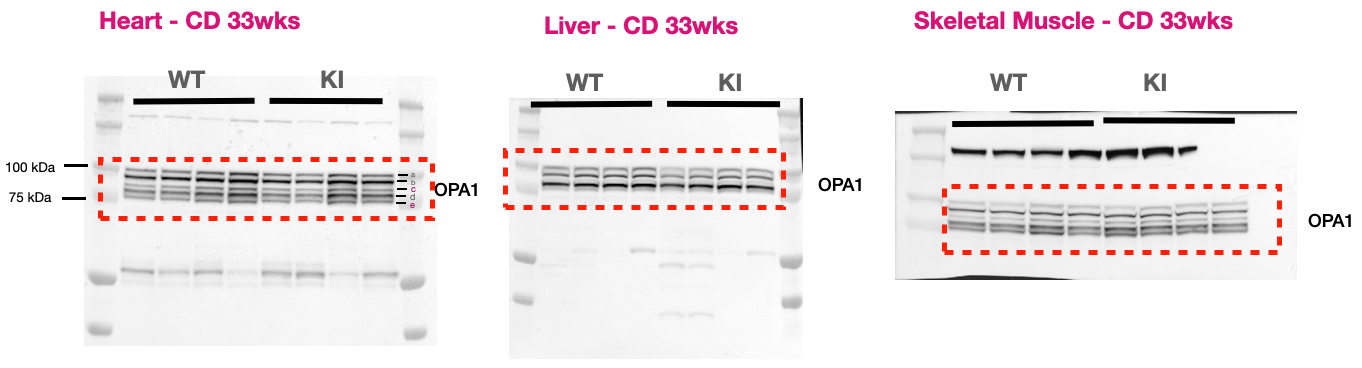

Supplement: Figure 4—figure supplement 3—source data 1. [file elife-82283-fig4-figsupp3-data1.zip › Fig4-SupFig3-Source data/F4S3_all_Opa.png]

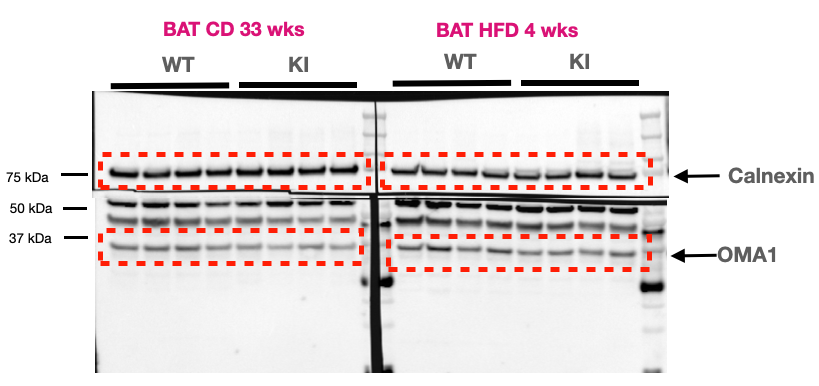

Supplement: Figure 4—figure supplement 4—source data 1. [file elife-82283-fig4-figsupp4-data1.zip › Fig4-SupFig4-Source data/F4S4_BAT33wk_OmaCanx.png]

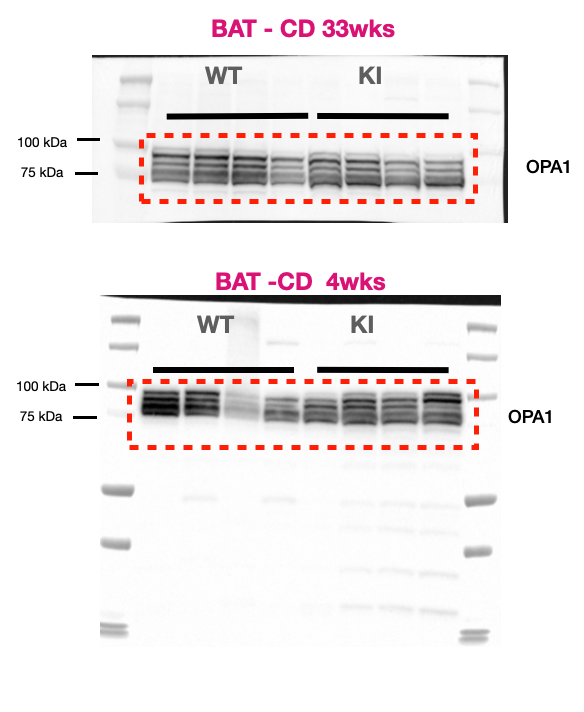

Supplement: Figure 4—figure supplement 4—source data 1. [file elife-82283-fig4-figsupp4-data1.zip › Fig4-SupFig4-Source data/F4S4_Bat_Opa.png]

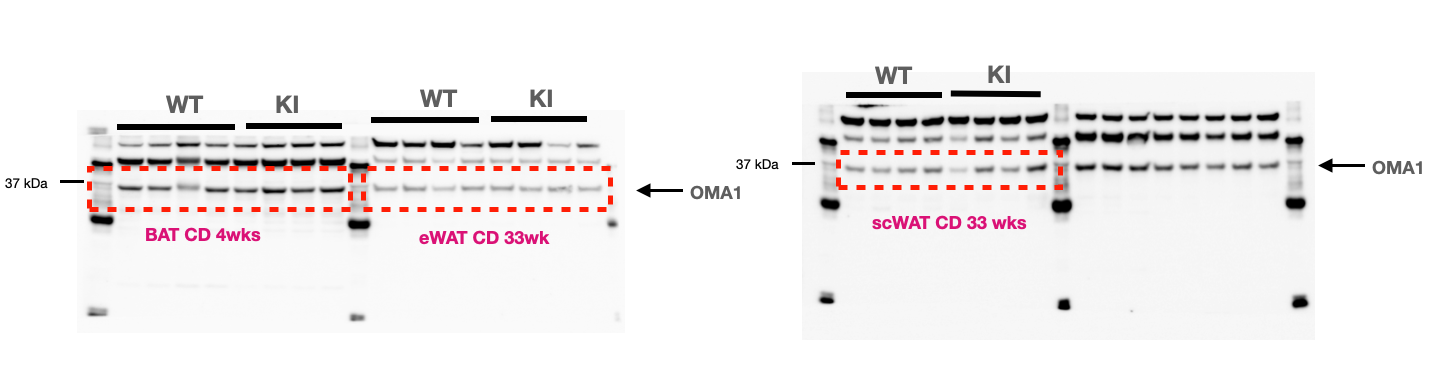

Supplement: Figure 4—figure supplement 4—source data 1. [file elife-82283-fig4-figsupp4-data1.zip › Fig4-SupFig4-Source data/F4S4_other_Oma1.png]

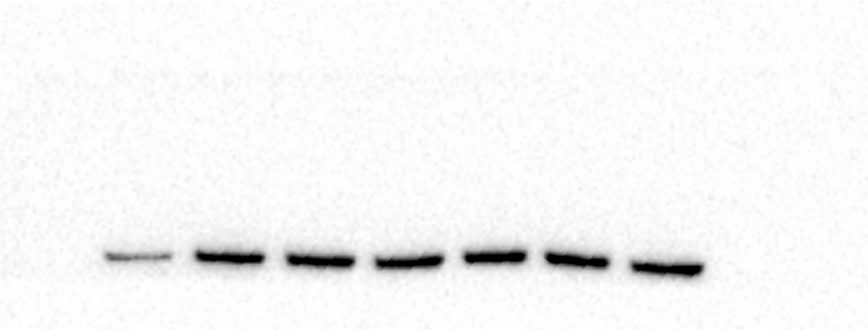

Supplement: Figure 5—source data 1. [file elife-82283-fig5-data1.zip › Fig5-Source data/Calnexin_raw.tif]

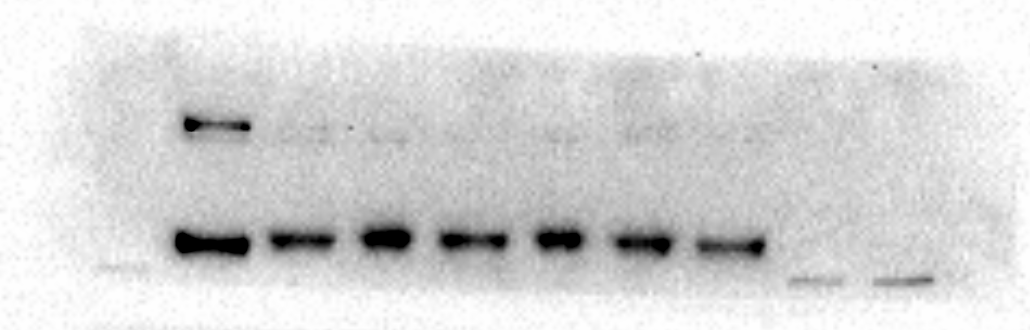

Supplement: Figure 5—source data 1. [file elife-82283-fig5-data1.zip › Fig5-Source data/InsRbeta_raw.tif]

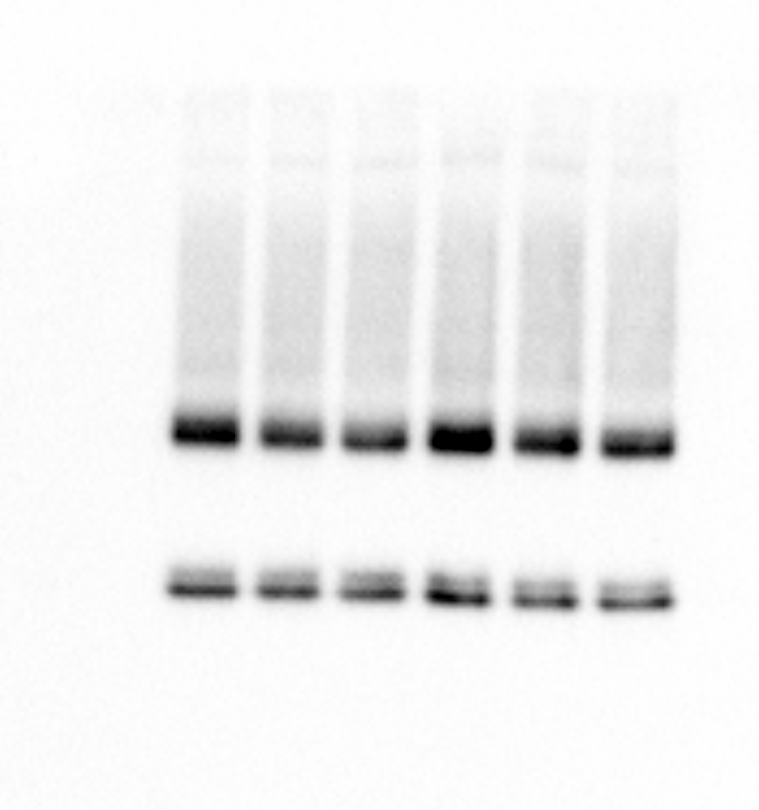

Supplement: Figure 5—source data 1. [file elife-82283-fig5-data1.zip › Fig5-Source data/Adiponectin_raw.tif]

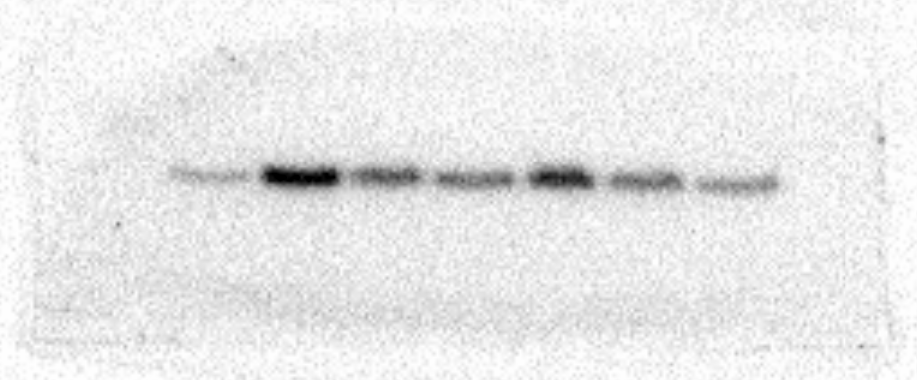

Supplement: Figure 5—source data 1. [file elife-82283-fig5-data1.zip › Fig5-Source data/Leptin_raw.tif]

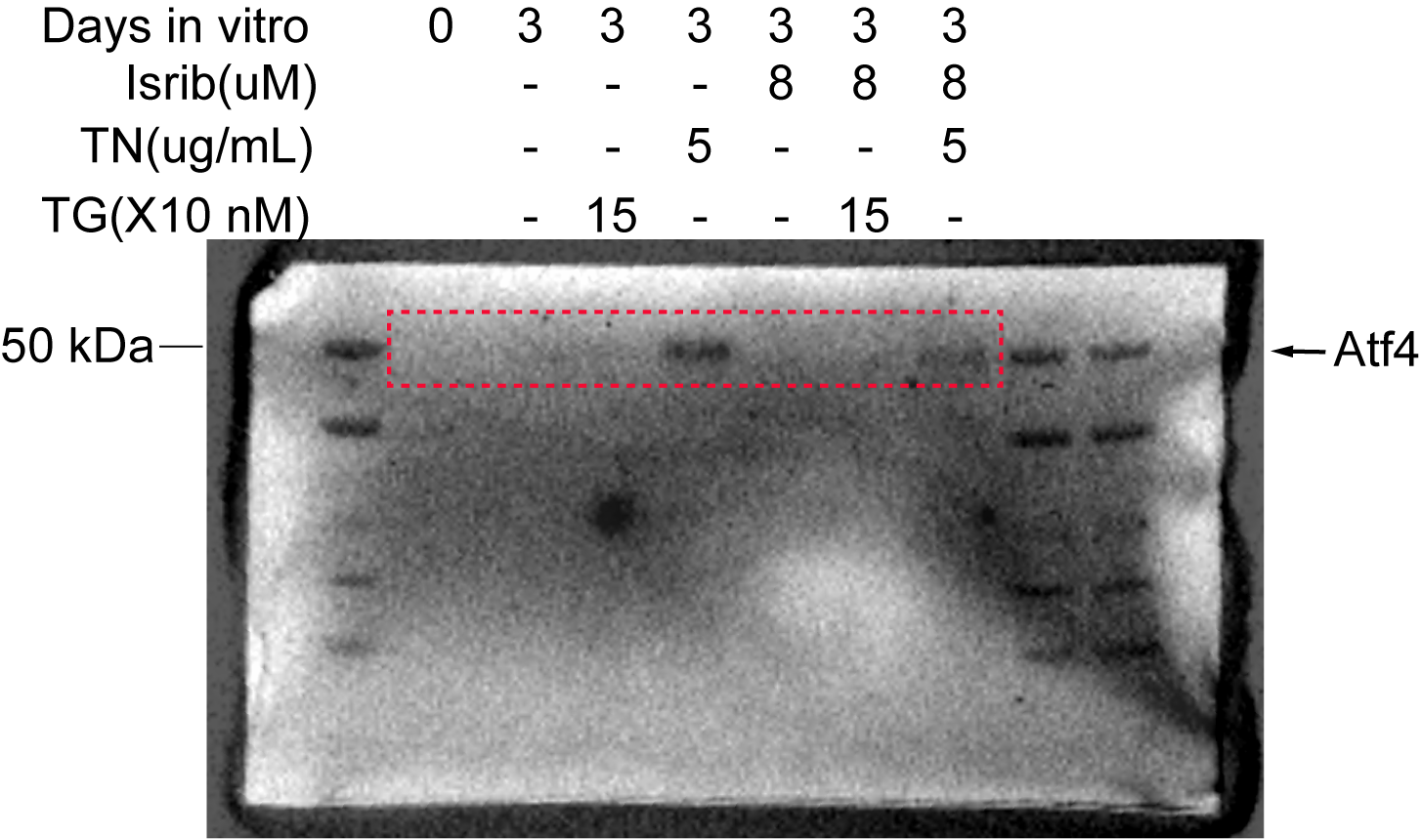

Supplement: Figure 5—source data 1. [file elife-82283-fig5-data1.zip › Fig5-Source data/Atf4_annotated.tif]

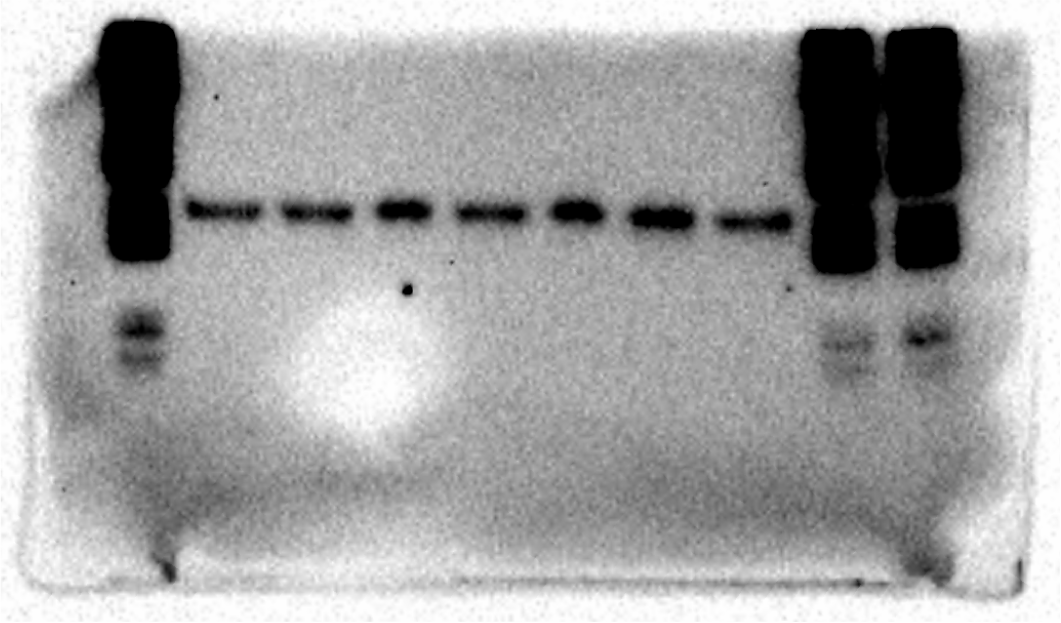

Supplement: Figure 5—source data 1. [file elife-82283-fig5-data1.zip › Fig5-Source data/Total Eif2╬▒_raw.tif]

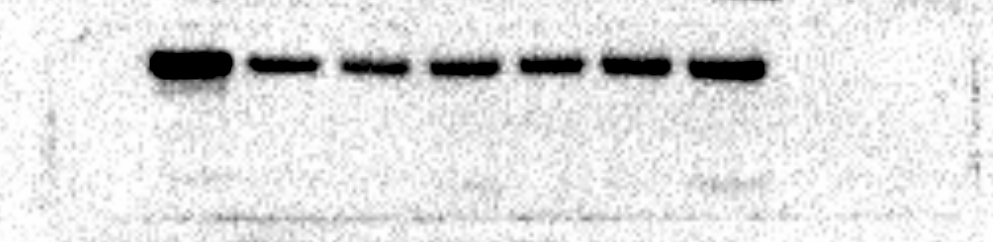

Supplement: Figure 5—source data 1. [file elife-82283-fig5-data1.zip › Fig5-Source data/Akt_raw.tif]

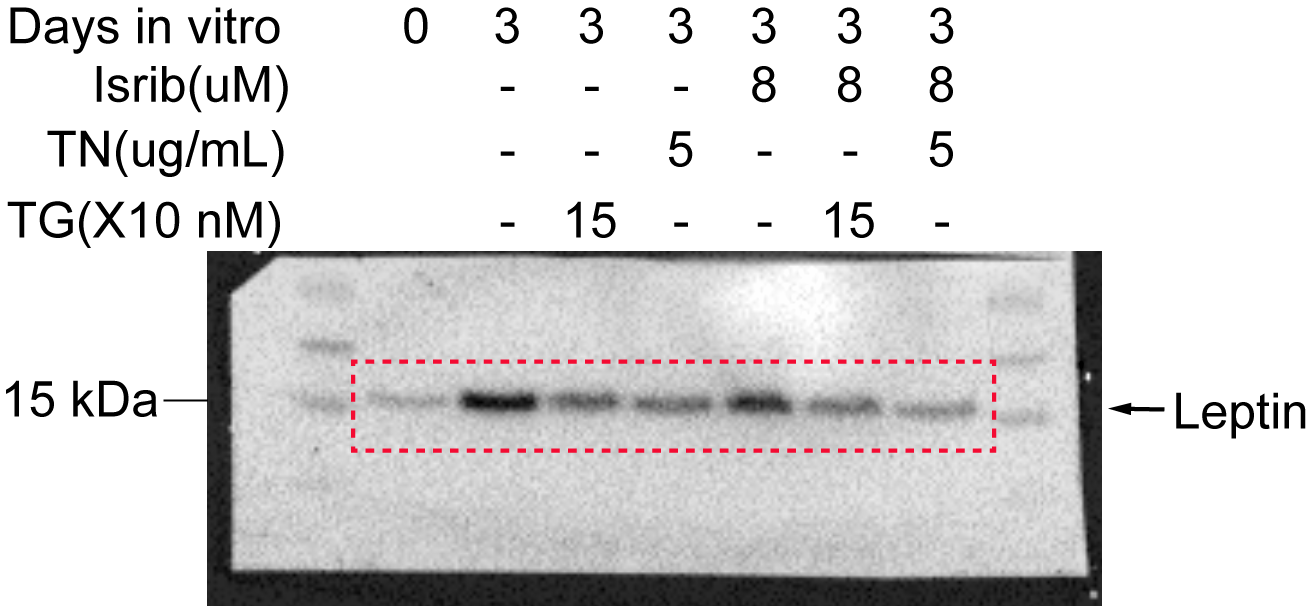

Supplement: Figure 5—source data 1. [file elife-82283-fig5-data1.zip › Fig5-Source data/Leptin_annotated.tif]

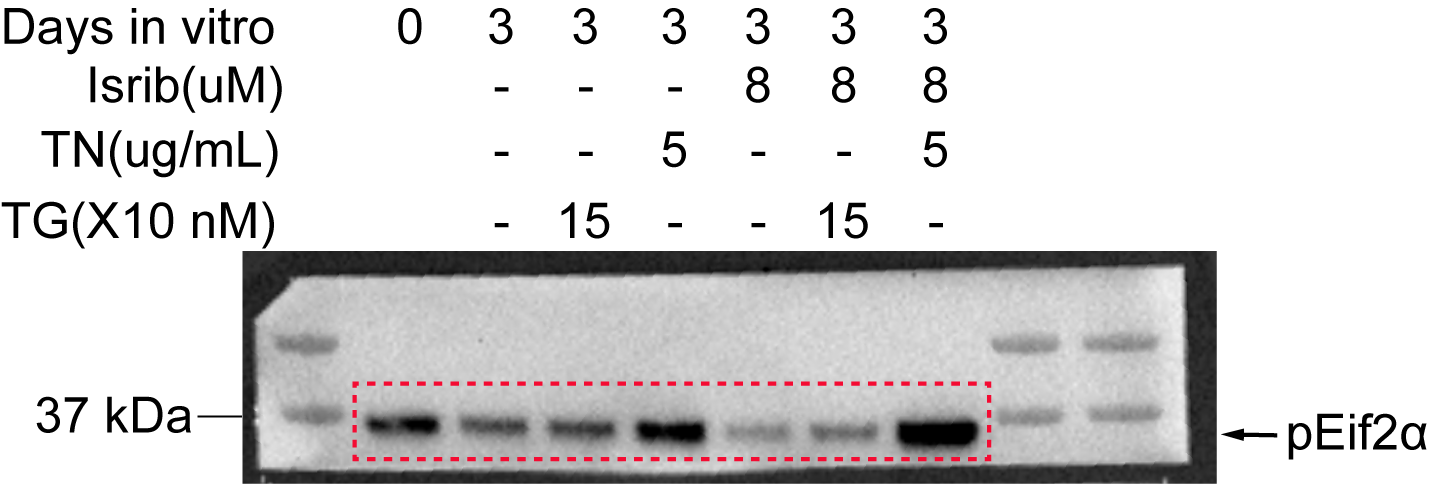

Supplement: Figure 5—source data 1. [file elife-82283-fig5-data1.zip › Fig5-Source data/pEif2a S51_annotated.tif]

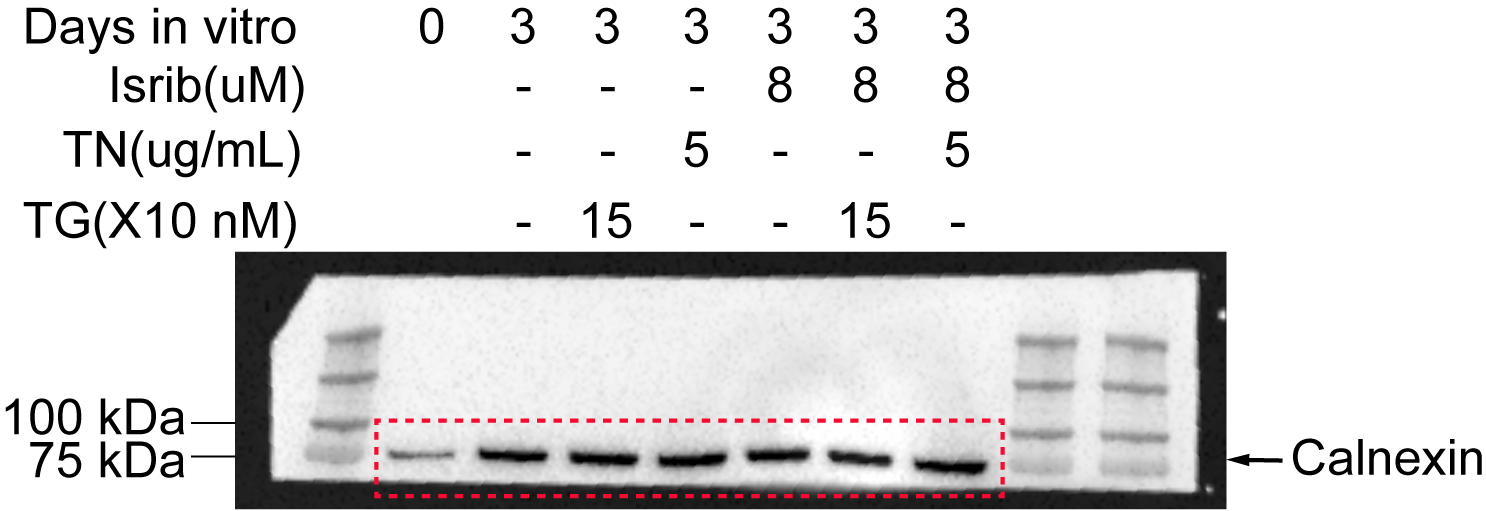

Supplement: Figure 5—source data 1. [file elife-82283-fig5-data1.zip › Fig5-Source data/Calnexin_annotated.tif]

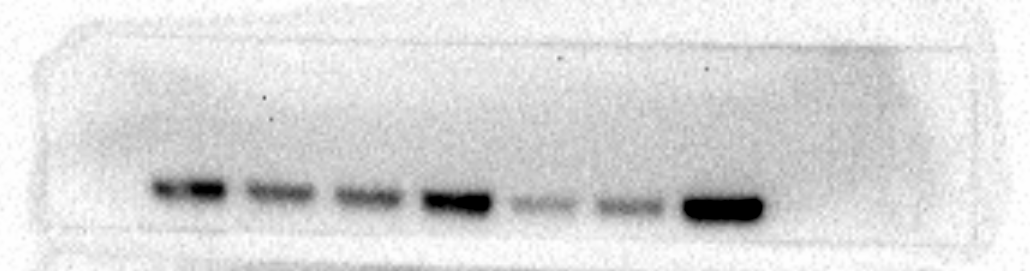

Supplement: Figure 5—source data 1. [file elife-82283-fig5-data1.zip › Fig5-Source data/pEif2╬▒ S51_raw.tif]

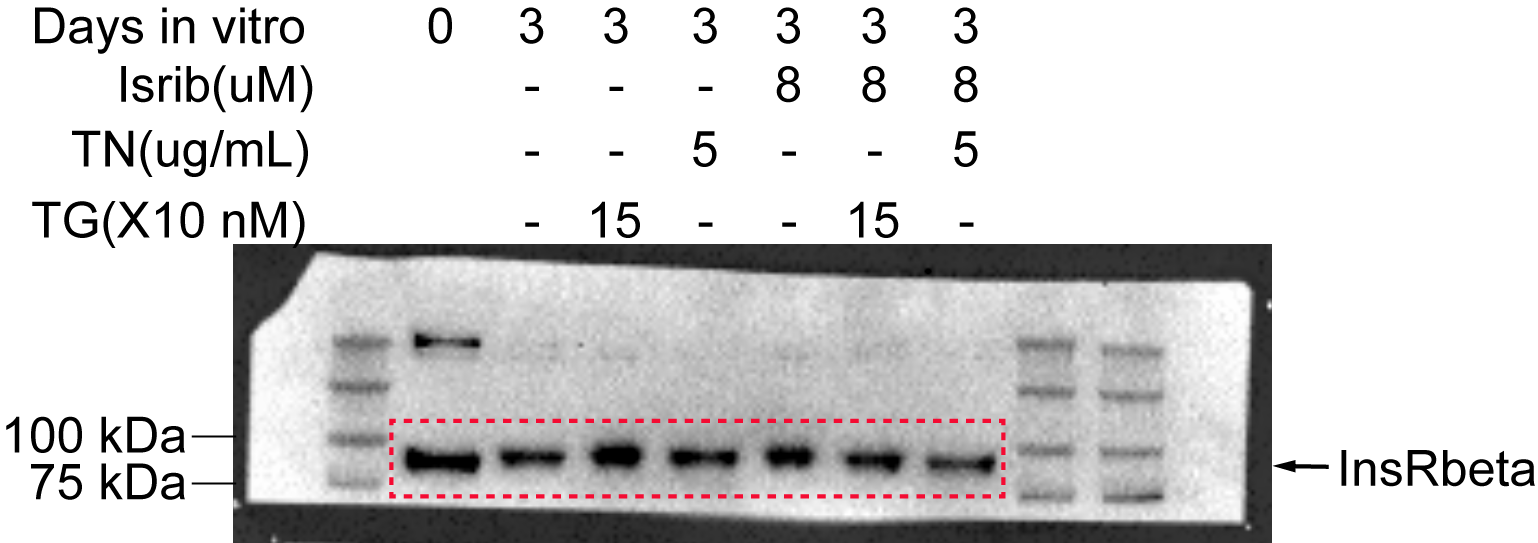

Supplement: Figure 5—source data 1. [file elife-82283-fig5-data1.zip › Fig5-Source data/InsRbeta_annotated.tif]

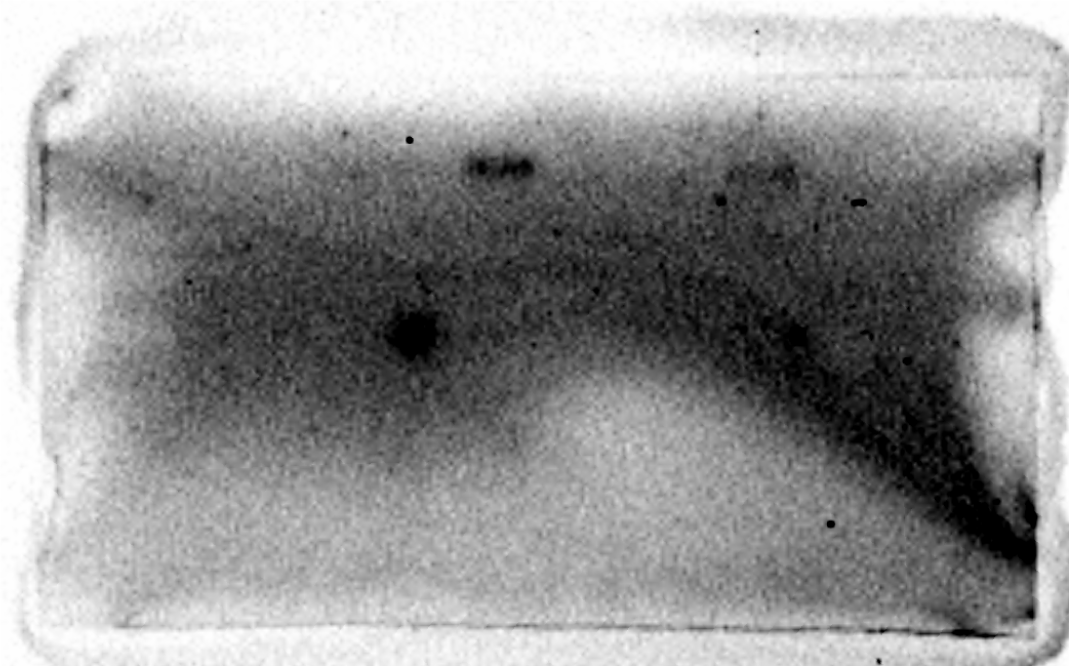

Supplement: Figure 5—source data 1. [file elife-82283-fig5-data1.zip › Fig5-Source data/Atf4_raw.tif]

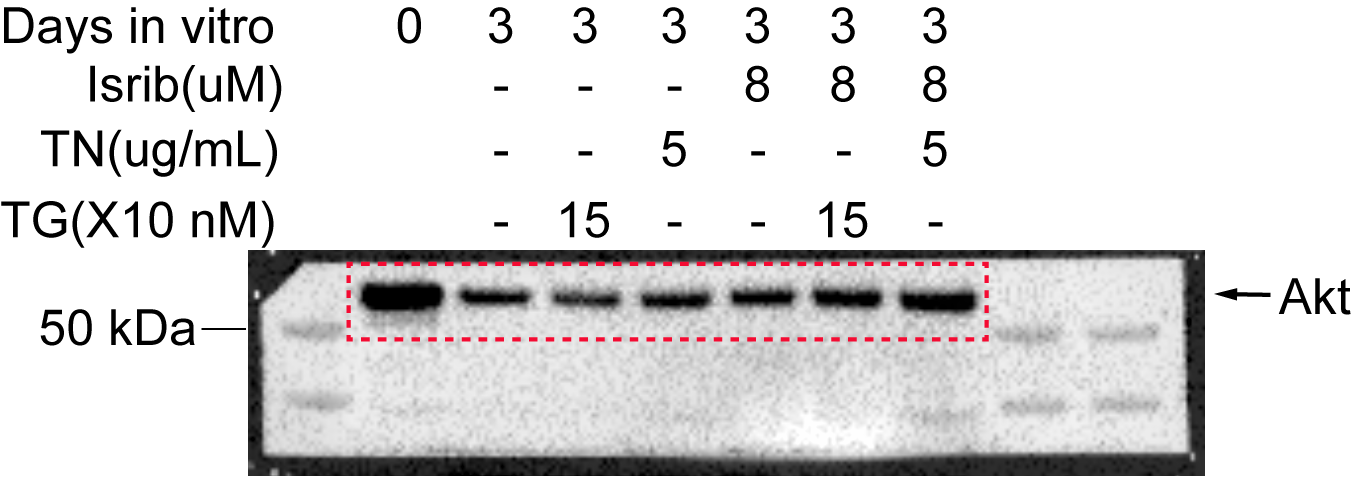

Supplement: Figure 5—source data 1. [file elife-82283-fig5-data1.zip › Fig5-Source data/Akt_annotated.tif]

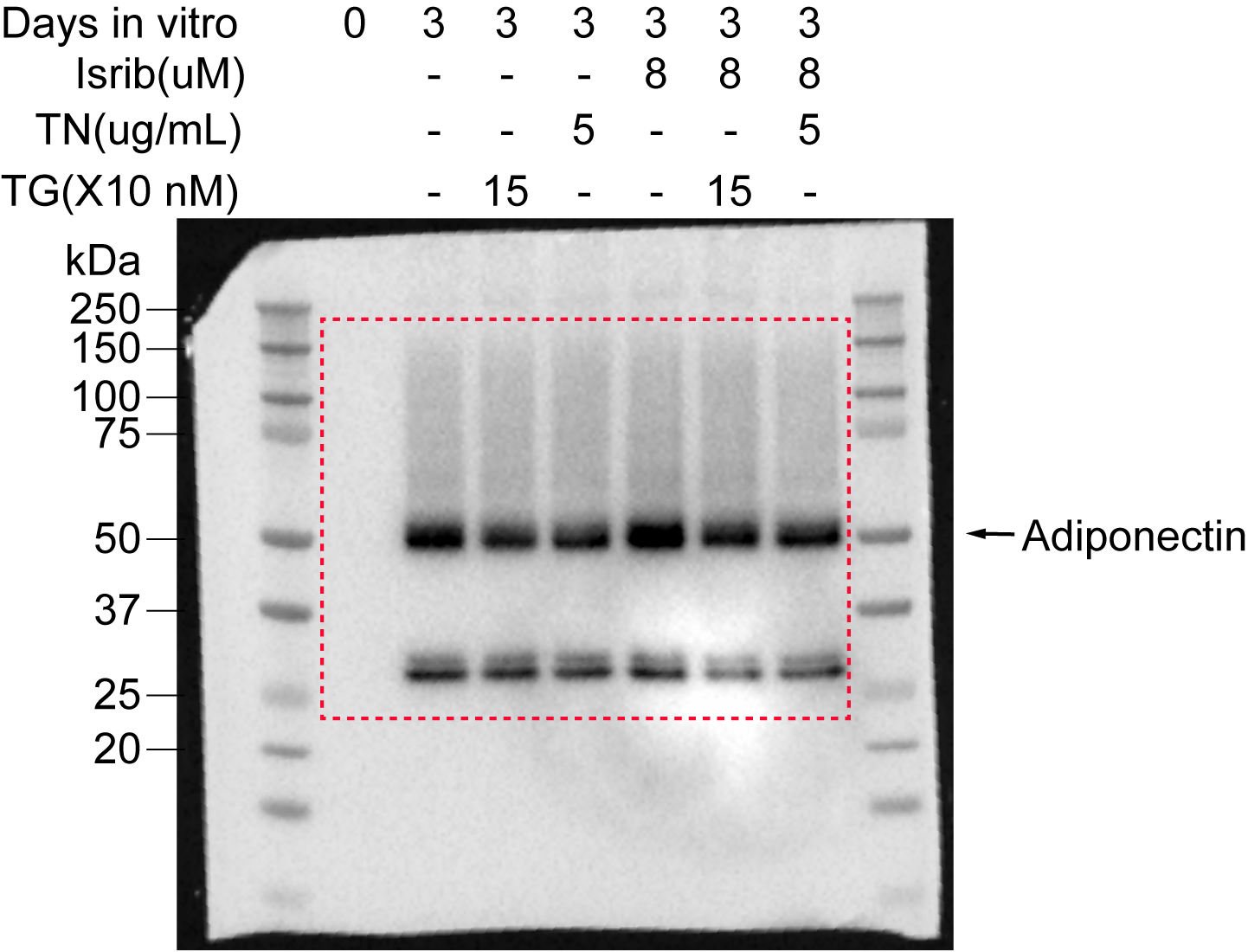

Supplement: Figure 5—source data 1. [file elife-82283-fig5-data1.zip › Fig5-Source data/Adiponectin_annotated.tif]

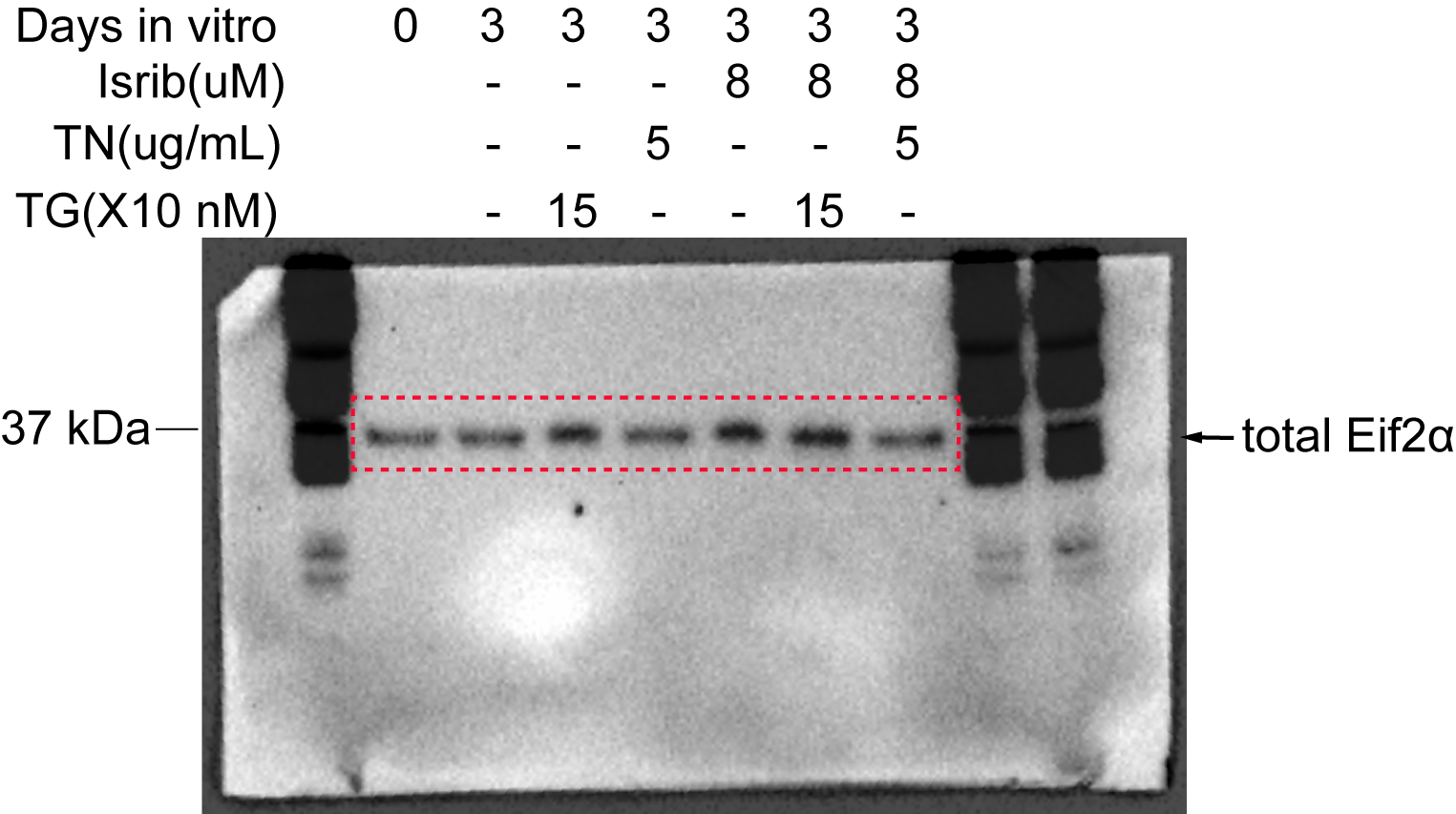

Supplement: Figure 5—source data 1. [file elife-82283-fig5-data1.zip › Fig5-Source data/Total Eif2a_annotated.tif]
