# Supplementary material for: A mouse model of human mitofusin-2-related lipodystrophy exhibits adipose-specific mitochondrial stress and reduced leptin secretion
Source: eLife. 2023 Feb 1;12:e82283. doi: 10.7554/eLife.82283 (PMC9937658; doi:10.7554/eLife.82283)
Supplement: Supplementary file 1. [file elife-82283-supp1.docx]

| **Primary** | **Supplier** | **CatID** | **Concentration** | **Diluent** | **2^ary^** |
| --- | --- | --- | --- | --- | --- |
| Adiponectin | Abcam | ab85827 | 1 in 1000 | 5% milk | Mouse |
| AKT | Cell signalling | 2920S | 1 in 1000 | 5% BSA | Mouse |
| Anti-mouse HRP | Cell signalling | 70765 | 1 in 5000 | 5% milk | N/A |
| Anti-rabbit HRP | Cell signalling | 70751 | 1 in 5000 | 5% milk | N/A |
| Atf4 | Cell signalling | 11815S | 1 in 1000 | 5% BSA | Rabbit |
| Beta-tubulin | Abcam | ab6046 | 1 in 1000 | 5% milk | Rabbit |
| Calnexin | Abcam | ab22595 | 1 in 5000 | 5% BSA | Rabbit |
| CitSynth | Abcam | Ab129095 | 1 in 1000 | 5% milk | Rabbit |
| Gapdh | GeneTex | GTX100118 | 1 in 5000 | 5% milk | Rabbit |
| Gapdh (for Opa1 blots) | Proteintech | 10494-1-AP | 1 in 1000 | 5% milk | Rabbit |
| InsR beta | Santa cruz | SC-57342 | 1 in 1000 | 5% BSA | Mouse |
| Leptin | Abcam | ab9749 | 1 in 1000 | 5% BSA | Rabbit |
| Mfn1 | Abcam | Ab126575 | 1 in 250 | 5% milk | Mouse |
| Mfn2 | Cell signalling | D2D10 | 1 in 1000 | 5% milk | Rabbit |
| Mthfd2 | Proteintech | 12270-1-AP | 1 in 1000 | 5% milk | Rabbit |
| Oma1 | Proteintech | 17116-1-AP | 1 in 1000 | 5% milk | Rabbit |
| Opa1 | BD Biosciences | 612606 | 1 in 1000 | 5% milk | Mouse |
| OXPHOS cocktail | Abcam | Ab110413 | 1 in 1000 | 5% milk | Mouse |
| Phos-Eif2a | Epitomics | 10901 | 1 in 1000 | 5% BSA | Rabbit |
| Tom20 | Abcam | Ab56783 | 1 in 500 | 5% milk | Mouse |
| Total Eif2a | D. Ron Lab | N/A | 1 in 3000 | 5% milk | Rabbit |

**Supplementary File 1**: Antibodies used in this study.
