# Supplementary material for: A mouse model of human mitofusin-2-related lipodystrophy exhibits adipose-specific mitochondrial stress and reduced leptin secretion
Source: eLife. 2023 Feb 1;12:e82283. doi: 10.7554/eLife.82283 (PMC9937658; doi:10.7554/eLife.82283)
Supplement: Supplementary file 2. — Fwd, forward primer; Rv, reverse primer. [file elife-82283-supp2.docx]

| **Primer** | **Sequence/TaqMan** |
| --- | --- |
| 36b4 Fwd | AGATGCAGCAGATCCGCAT |
| 36b4 Rv | GTTCTTGCCCATCAGCACC |
| AdipoQ Fwd | GTTGCAAGCTCTCCTGTTCC |
| AdipoQ Rv | ATCCAACCTGCACAAGTTCC |
| Atf4 Fwd | GGGTTCTGTCTTCCACTCCA |
| Atf4 Rv | AAGCAGCAGAGTCAGGCTTTC |
| Atf5 TaqMan | Cat# Mm04179654_m1 (ThermoFisher Scientific) |
| B2m Fwd | ACTGATACATACGCCTGCAGAGTT |
| B2m Rv | TCACATGTCTCGATCCCAGTAGA |
| Ddit3 (Chop) Fwd | CCACCACACCTGAAAGCAGAA |
| Ddit3 (Chop) Rv | AGGTGAAAGGCAGGGACTCA |
| Fgf21 Fwd | CTGGGGGTCTACCAAGCATA |
| Fgf21 Rv | CACCCAGGATTTGAATGACC |
| Gdf15 TaqMan | Cat# Mm00442228_m1 (ThermoFisher Scientific) |
| gDNA_HK2_Fwd | GCCAGCCTCTCCTGATTTTAGTGT |
| gDNA_HK2_Rev | GGGAACACAAAAGACCTCTTCTGG |
| gDNA_Mt-Rnr2_Fwd | AACTCGGCAAACAAGAACCC |
| gDNA_Mt-Rnr2_Rev | CCCTCGTTTAGCCGTTCATG |
| Hprt Fwd | AGCCTAAGATGAGCGCAAGT |
| Hprt Rv | GGCCACAGGACTAGAACACC |
| Lep3 Fwd | CCAGGAtgacaccaaaaccct |
| Lep3 Rv | GATACCGACTGCGTGTGTGA |
| Mfn2 genotyping Fwd | AGTCCCTTCCTTGTCACTTAGT |
| Mfn2 genotyping Rv | ATCTCACAAGAAAGCGAAATCC |

**Supplementary File 2**: Primer sequences used in this study. Fwd, forward primer; Rv, reverse primer.
